# Coordination isomerism in dioxophosphorane cyanides†

**DOI:** 10.1039/d4sc07636b

**Published:** 2025-03-13

**Authors:** Ayu Afiqah Nasrullah, Edgar Zander, Fabian Dankert, Andrey Petrov, Jonas Surkau, Eszter Baráth, Christian Hering-Junghans

**Affiliations:** a Leibniz Institut für Katalyse e.V. (LIKAT) A.-Einstein-Str. 29a 18059 Rostock Germany christian.hering-junghans@catalysis.de eszter.barath@catalysis.de; b Pusat Persediaan Sains dan Teknologi, Universiti Malaysia Sabah Jln UMS 88400 Kota Kinabalu Sabah Malaysia; c Universität Kassel, Institut für Chemie Heinrich-Plett-Straße 40 34132 Kassel Germany; d Institut für Chemie, Universität Rostock Albert-Einstein-Straße 3a 18059 Rostock Germany

## Abstract

The 1,3-phosphaazaallene ^Dipp^TerP = C=N*t*Bu (^Dipp^Ter = 2,6-(2,6-iPr_2_C_6_H_3_)_2_–C_6_H_3_) is thermally labile towards iso-butene elimination and formation of the corresponding cyanophosphine ^Dipp^TerP(H)CN (1). In previous work we have shown facile deprotonation of 1 with K[N(SiMe_3_)_2_ and formation of cyanophosphide [(^Dipp^TerPCN)K]. We now present the alkali metal tethered cyanophosphides [(^Dipp^TerPCN)M(crown)] (M = Na, K; crown = 15-c-5, 18-c-6) and their structural diversity in the solid state depending on the metal (M) and the crown ether. Facile oxidation of [^Dipp^TerPCN][M(crown)] with O_2_ yields the formal cyanide adducts of dioxophosphoranes [^Dipp^TerPO_2_(CN)]^−^. Surprisingly, [^Dipp^TerPO_2_(CN)]^−^ is obtained as a mixture of the cyanide and isocyanide isomers, indicating a coordination isomerism. This phenomenon is corroborated by experimental and theoretical studies revealing the cyanide isomer to be thermodynamically more stable. The oxidation with elemental sulphur gave the corresponding dithiophosphorane cyanide adduct [^Dipp^TerPS_2_(CN)]^−^, in which no isomerism was observed. This points to a crucial role of triplet oxygen in the isomerisation process. Monooxidation occurs when [^Dipp^TerPO_2_(CN)]^−^ salts were treated with N_2_O, giving formal anionic phoshinidene monoxide adducts.

## Abstract 2

Cyanophosphides are shown to react with oxygen to give dioxophosphorane cyanide adducts as a mixture of cyanide and isocyanide isomers.

## Graphical abstract

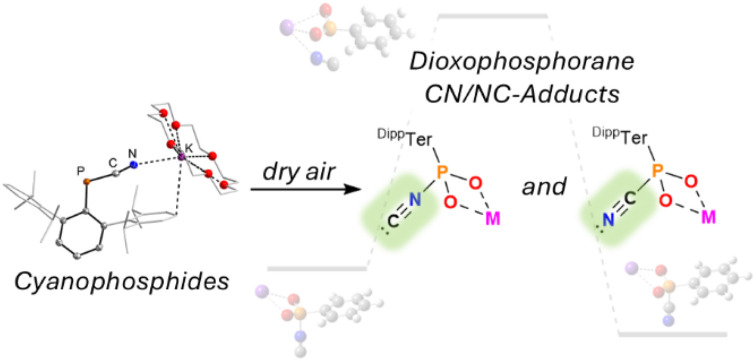

## Introduction

**Scheme 1:**
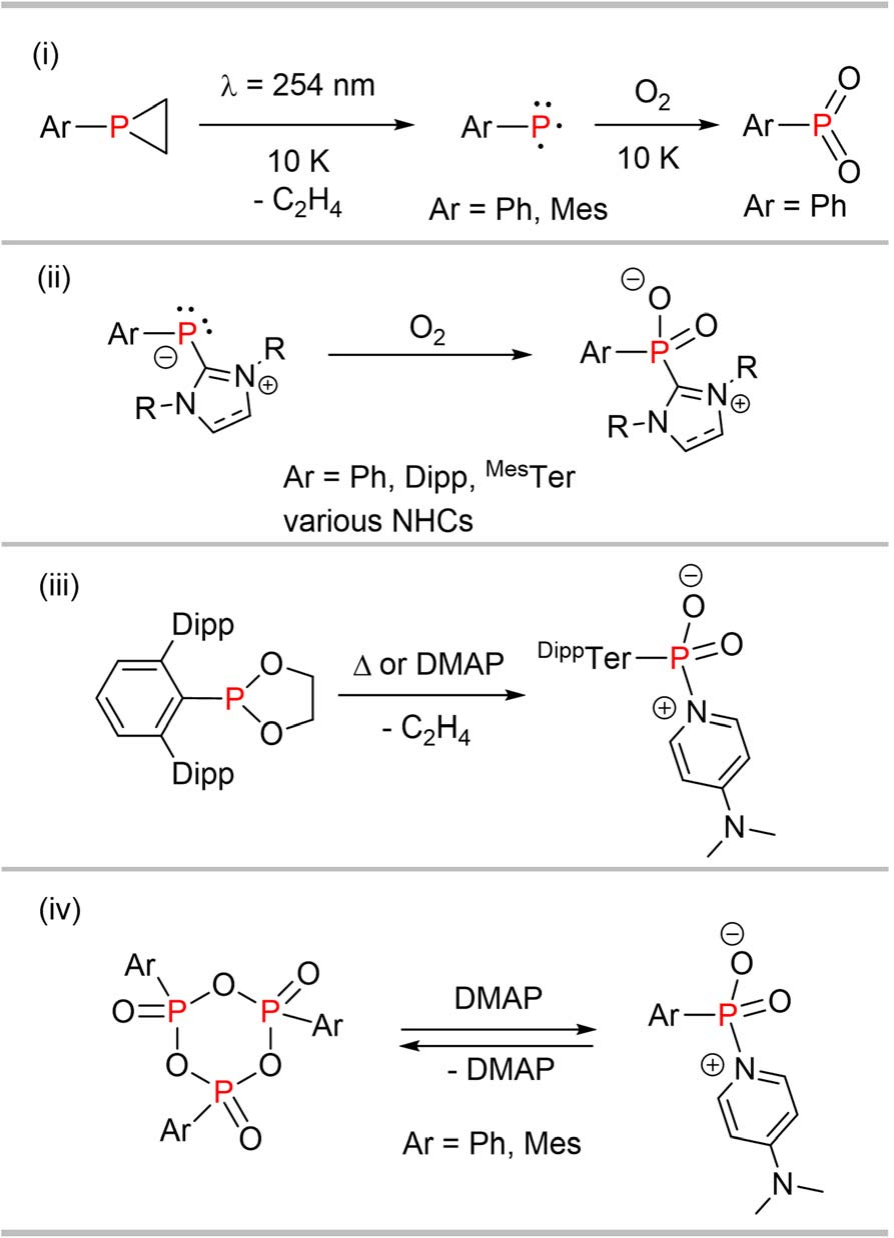
(i) Generation of phosphinidenes and dioxophosphoranes in Ar matrices. (ii) Synthesis of NHC dioxophosphorane adducts. (iii) DMAP-induced ethylene elimination to access ^Dipp^TerPO_2_(DMAP). (iv) Reversible DMAP coordination at dioxophosphoranes.

Aryl-substituted dioxophosphoranes (Ar–PO_2_) are the formal heavier analogues of nitroarenes, with a highly electrophilic phosphorus atom. Therefore, R–PO_2_ undergoes immediate self-aggregation to give cyclic dimers, trimers or linear oligomers.^1,2^ UV-irradiation (*λ* = 254 nm) of arylphosphiranes Ar–P(C_2_H_4_) (Ar = Ph,^3^ Mes^4^) in a solid argon matrix afforded the phosphinidenes Ar–P in their triplet ground-state (Scheme 1(i)). Subsequent reaction of Ar–P with molecular oxygen (^3^O_2_) at 10 K under irradiation gives phenyldioxophosphorane PhPO_2_. The formation of Ph–PO_2_ is expected to proceed through a triplet diradical/zwitterionic intermediate with a terminal Ph–P=O^(+)^–O^(−)^ unit, which then rearranges to give cyclic 3-phenyl-1,3,2-dioxophosphorane and eventually Ph–PO_2_.^3^ In this regard the mechanism may involve triplet–singlet curve crossings, which can only be addressed by sophisticated excited state computations. Considering the extreme electrophilicity of dioxophosphoranes, it is not surprising that mainly Lewis base adducts of R–PO_2_ have been reported.

Lewis-base stabilized species include the parent compound HPO_2_ which was isolated as its carbodiphosphorane adduct. The phosphinidene H–P is not an intermediate in this case, though.^5^ The exposure of NHC phosphinidene Ar–P(NHC) adducts (NHC = N-heterocyclic carbene) to dry air yields the corresponding dioxophosporane NHC adducts of the type R–PO_2_(NHC) (Scheme 1(ii)).^6–8^ A cyclic phosphine-stabilized amino-dioxophosphorane was synthesized in similar fashion.^9^ The OPMe_3_ adduct of ^Dipp^TerPO_2_ [^Dipp^TerPO_2_(OPMe_3_)] (^Dipp^Ter = 2,6-(2,6-iPr_2_C_6_H_2_)–C_6_H_3_) was recently reported, as an unexpected product in the reaction of ^Dipp^TerP(PMe_3_) with SO_2_.^10^ The dimer of ^Dipp^TerPO_2_, [^Dipp^TerPO_2_]_2_,^11^ was afforded by thermal C_2_H_4_ liberation from ^Dipp^TerP(OCH_2_)_2_ or by the addition of catalytic amounts of pyridine or DMAP (DMAP = 4-dimethylaminopyridine) as a Lewis base (Scheme 1(iii)). Cyclic triphosphonates (RPO_2_)_3_, the trimers of the corresponding dioxophosphoranes, give an equilibrium mixture with DMAP in CDCl_3_ containing the trimer and the RPO_2_(DMAP) adduct, substantiating ligand lability in R–PO_2_ base adducts (Scheme 1(iv)).^12^

**Scheme 2:**
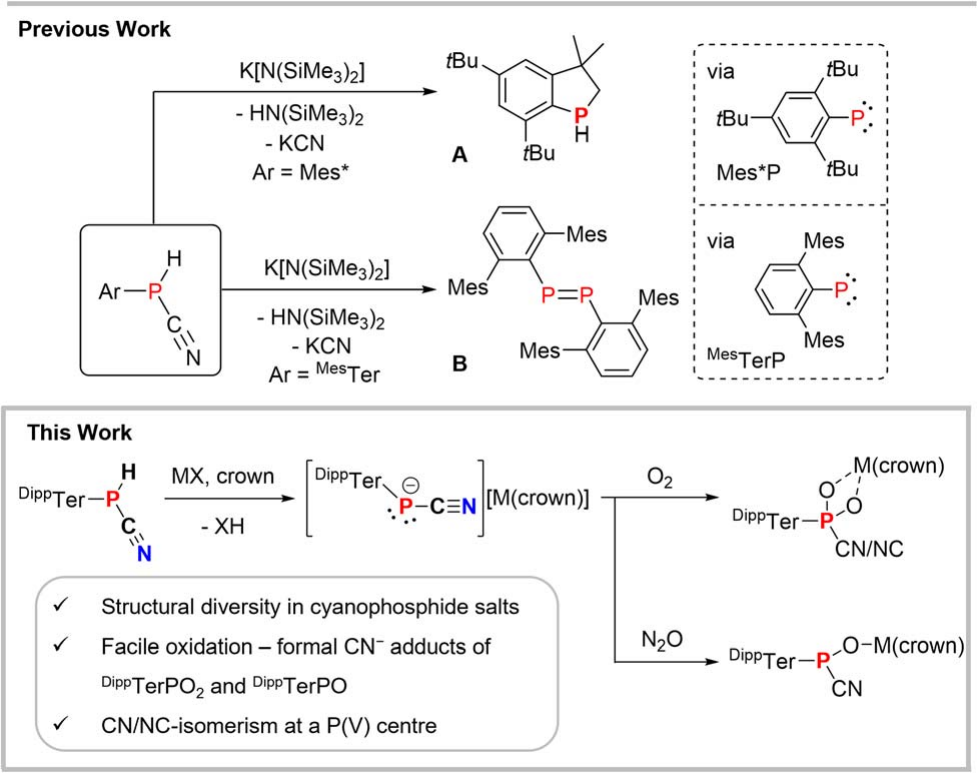
Cyanophosphines as sources of phosphinidenes and summary of this work.

Our group has recently shown that deprotonation of the cyanophosphines ArP(H)CN (Ar = Mes*, 2,4,6-*t*Bu_3_-C_6_H_2_; ^Mes^Ter, 2,6-(2,4,6-Me_3_C_6_H_2_)–C_6_H_3_; ^Dipp^Ter) with K[N(SiMe_3_)_2_] (KHMDS) afforded the cyanophosphides [(ArPCN)K].^13^ Even though [(Mes*PCN)K] (*δ*(^31^P) = −146.2 ppm) could be generated in solution, thermal KCN elimination occurred within 16 h at ambient temperature, to give a phosphaindane (A, Scheme 2) as the main product, a decomposition product of the free phosphinidene Mes*-P.^14^^Mes^TerP(H)CN reacted similarly when treated with 1 eq. of KHMDS, to give after KCN elimination the diphosphene (^Mes^TerP)_2_ (B, Scheme 2).^15^ By contrast [(^Dipp^TerPCN)K], with a characteristic ^31^P NMR signal at −142 ppm and [^Dipp^TerPCN][K(2.2.2-crypt)] were found to be stable towards KCN elimination. It can thus be concluded that [Ar–P(CN)]^−^ are formal cyanide adducts of phosphinidenes and that air oxidation of the stable [(^Dipp^TerPCN)K] might result in the formation of the corresponding dioxophosphorane cyanide adducts.

In this contribution we report on a series of alkali metal tethered cyanophosphide [(^Dipp^TerPCN)M(crown)] (M = Na, K; crown = 18-crown-6, 15-crown-5) salts and outline their structural diversity in the solid state. In addition, the selective double oxidation of the phosphorus atoms in the cyanophosphides with ^3^O_2_ is described, giving formal cyanide adducts of dioxophosphoranes. In the case of [^Dipp^TerP(O_2_)CN][M(crown)] a nitrile/isonitrile coordination isomerism is observed, a phenomenon that is rare in main group chemistry. By contrast when using S_8_ as an oxidant only the dithiophosphorane cyanide adduct is detected, while with N_2_O oxygen atom transfer results in the cyanide phosphinidene oxide adducts.

## Results & discussion

### Structural variety in cyanophosphide salts

**Scheme 3:**
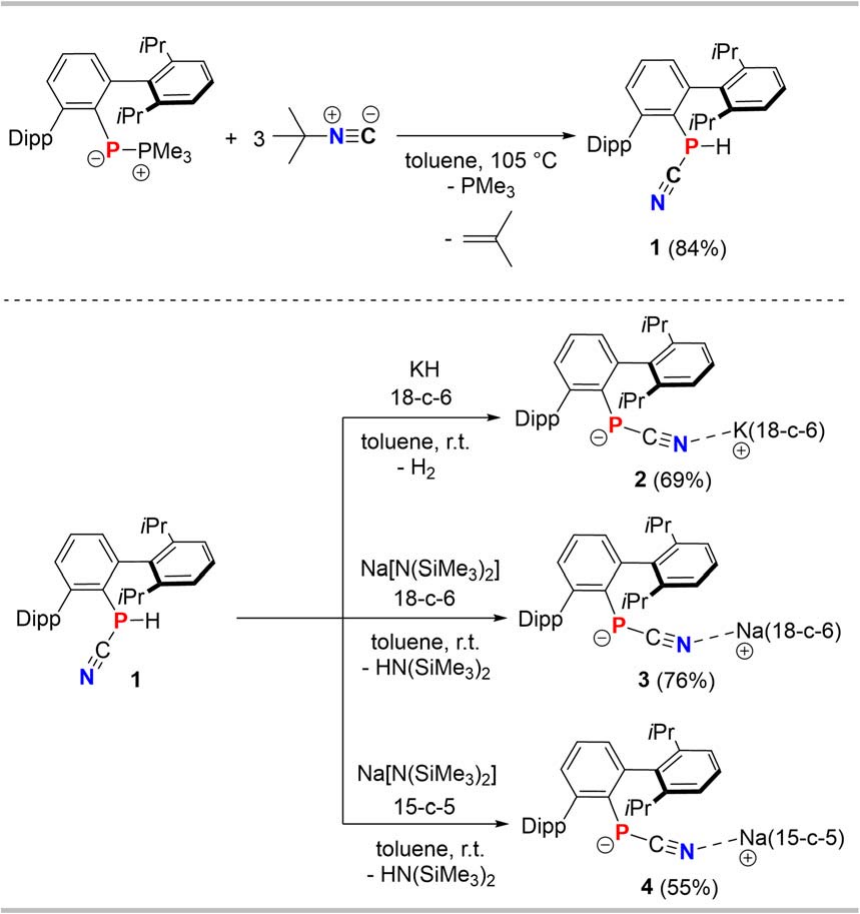
Optimized synthesis of 1 and syntheses of cyanophosphide salts 2–4.

First, the synthesis of the cyanophoshine ^Dipp^TerP(H)CN (1) was optimized and was performed on a gram-scale (Scheme 3, top).‡‡Caution! When working with PCN-compounds the release of hydrogen cyanide, especially when cleaning glassware, is possible. Therefore, additional safety precautions and a special waste treatment with hydrogen peroxide are recommended. The combination of ^Dipp^TerP(PMe_3_) with three equivalents *t*Bu-NC in toluene and heating to 105 °C for 90 h afforded 1 (*δ*(^31^P{^1^H}) = −120.4 ppm in C_6_D_6_) as an off-white powder in 84% yield (*cf.* ESI† p. S16 ff.). On a larger scale an excess of the isonitrile and pro-longed heating is required. We first drew our attention towards the stability of [^Dipp^TerPCN]^−^ with respect to the alkali metal ion and different crown ethers. Therefore, the cyanophosphide species [(^Dipp^TerPCN)M(L)] (M = K, Na; L = 18-c-6, 15-c-5) were synthesized and comprehensively characterized (Scheme 3, bottom). Deprotonation of 1 with KH, instead of KHMDS, in the presence of 18-c-6 in toluene was accompanied by gas evolution to give a yellow solution. Using KH can be viewed as an alternative base when HN(SiMe_3_)_2_ as a byproduct needs to be avoided. A similar color change to yellow is observed when Na[N(SiMe_3_)_2_] is used as a base, irrespective of whether 15-c-5 or 18-c-6 is utilized.

The formation of the cyanophosphide salts is indicated in the ^1^H NMR spectra in C_6_D_6_ after 2 h by the absence of a PH unit and minimally shielded ^31^P NMR signals at *ca.* −128 ppm (Table 1). This is indicative of the quantitative formation of the contact ion pairs [(^Dipp^TerPCN)M(crown)] (2, M = K, crown = 18-c-6; 3, M = Na, crown = 18-c-6; 4, M = Na, crown = 15-c-5). After recrystallization from toluene at −30 °C, yellow single crystals suitable for SC-XRD experiments of all species were afforded in moderate isolated yields.

**Table 1:** Selected bond lengths [Å], angles [°] of 2, 3 and 4 and ^31^P NMR shifts as well as characteristic IR band for the PCN unit. * Shown in Fig. 1

Compound	P–C_CN_	C_CN_–N	N–M	P–C_CN_–N	C_Ar_–P–C_CN_	*δ*(^31^P) [ppm]	*ν̃* (PCN) [cm^−1^]
2 (M = K)	1.770(1)	1.163(2)	2.837(1)	168.6(1)	104.17(5)	−127.2	2045
3^a^ (M = Na)	1.767(2)	1.164(3)	2.388(2)	166.4(2)	106.36(8)	−129.3	2049
	1.762(2)*	1.160(3)*	2.417(2)*	165.7(2)*	105.29(8)*		
4^a^ (M = Na)	1.752(2)*	1.159(2)*	2.321(2)*	165.5(1)*	106.85(7)*	−129.9	2059
	1.757(2)	1.157(2)	2.334(2)	169.0(2)	104.00(7)		

a Two independent molecules in the asymmetric unit.

**Fig. 1:**
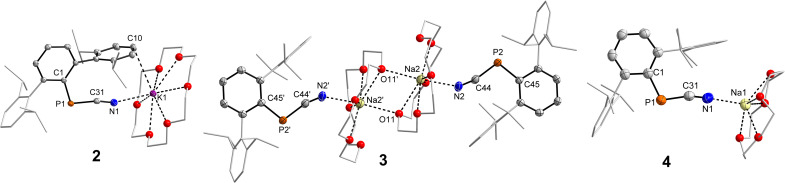
Molecular structures of 2, 3 (one of two ion pairs in the asymmetric unit (AsU), ′ symmetry generated: 1 − *x*, 1 − *y*, 1 − *z*) and 4 (one ion pair of two in the AsU). Hydrogen atoms omitted for clarity. Dipp groups shown with mixed wireframe/thermal ellipsoid representations. The iPr-groups and ethylene bridges in the crown ethers are rendered as wireframes. Oxygen atoms rendered as spheres with arbitrary radius. All thermal ellipsoids are drawn at the 50% probability level. Selected bond lengths and angles are shown in Table 1.

2, 3 and 4 crystallize in the triclinic space group *P*1̄ (Fig. 1, *cf.* Fig. S3 and S4†) with varying amounts of toluene in the unit cell. In the solid state there is a structural variety, which will be outlined in the following. In contrast to [^Dipp^TerPCN][K(2.2.2-crypt)],^13^ the N atom of the PCN unit in 2 (Fig. 1, left), 3 (Fig. 1, middle) and 4 (Fig. 1, right) shows a rather close contact to the alkali metal ion in the [M(crown)]^+^ fragment (2: *d*(N1–K1) = 2.837(1); 3: *d*(N–Na) = 2.387(2), 2.417(2); 4: *d*(N–Na) = 2.321(2), 2.334(2) Å; *cf.* Σ*r*_vdW_(K–N) = 4.3; (Na–N) = 3.82 Å). Additionally, there are M–C_arene_ contacts within the sum of the van-der-Waals radii to one of the flanking Dipp groups in 2 (*d*(K1–C10) = 3.464; Σ*r*_vdW_(K–C) = 4.45 Å) and in one of the independent ion pairs in 4 (*d*(Na2–C63) = 3.106(2) Å; Σ*r*_vdW_(Na–C) = 3.97 Å).^16^ The P–C_CN_ and C_CN_–N atomic distances (Table 1) correspond to contracted P–C single (*cf.* Σ*r*_cov_(P–C) = 1.86 Å), and slightly longer C≡N triple bonds (*cf.* Σ*r*_cov_(C–N) = 1.14 Å), respectively.^17^ The P–C_CN_–N (*ca.* 165°) and C_Ar_–P–C_CN_ angles (*ca.* 105°) are generally wider when compared to the previously reported [K(2.2.2-crypt]^+^ salt.^13^ Overall, the [^Dipp^TerPCN]^−^ unit closely resembles the isoelectronic ^Dipp^TerPCO (d(P–C_CO_) = 1.683(1), *d*(C–O) = 1.156(1) Å),^18^ and the CN stretching frequency of *ca.* 2050 cm^−1^ (Table 1) indicates substantial π-electron delocalization in the cyanophosphide unit. Interestingly, in 3 beside a monomeric [(^Dipp^TerPCN)Na(18-c-6)] ion pair, a dimeric [(^Dipp^TerPCN)Na(18-c-6)]_2_ unit was found. The centrosymmetric dimer (Fig. 1, middle), in which the 18-c-6 molecule occupies the equatorial plane of a distorted hexagonal bipyramid around the sodium ions, is formed through coordination of one of the 18-c-6 oxygen atoms to the second sodium, while the second axial position of each sodium in the dimer is occupied by the cyanide N atoms (*d*(N2–Na2 = 2.4172(18) Å; *d*(O11–Na2) = 2.609(3) Å). There are only few examples of dimeric [Na(18-c-6)]_2_^2+^ ion pairs,^19,20^ and the coordination mode is reminiscent to the one found in [(18-c-6)_2_Na_2_(H_2_O)_2_]_0.5_[Cd(SCN)_3_].^21^

It can thus be concluded that the alkali metal ion and the crown ether marginally influence the structure in solution, as indicated by similar NMR data. In the solid state though, significant differences are observed, which can be attributed to packing in the crystal and weak interactions of the alkali metal ion and the flanking ^Dipp^Ter groups.

### Dioxophosphorane cyanide adducts

An initial attempt to crystallize 4 from toluene in the freezer afforded besides yellow microcrystalline material, colorless blocks suitable for SC-XRD experiments. These were identified as the oxidation product [^Dipp^TerPO_2_(CN)][Na(15-c-5)] (4·O_2_).

**Fig. 2:**
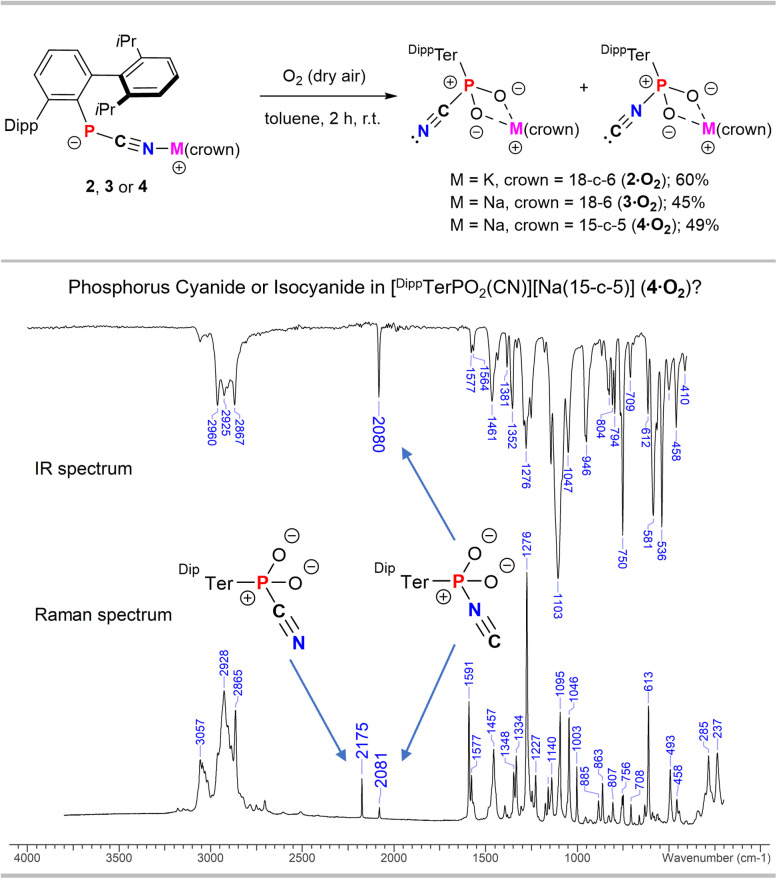
Synthesis of 2·O_2_, 3·O_2_ and 4·O_2_ (top) and depiction of the IR- and Raman spectra of 4·O_2_.

^31^P NMR data recorded from the few isolated crystals of 4·O_2_ showed a signal at −8.3 ppm, in line with those reported for other ArPO_2_(LB) species (*cf.* PhPO_2_(dmap) *δ*(^31^P) = 9.4 ppm).^12^ Concluding that traces of O_2_ affected the formation of 4·O_2_, akin to previous reports on NHC phosphinidene adducts,^6^ mixtures of 1, base and the respective crown ethers were properly degassed and reacted with 1 atm of dry air (Fig. 2, top).

After an initial color change to deep purple in all cases, the solution turned pale-yellow and ^31^P NMR spectroscopy of the reaction mixtures revealed the quantitative formation of 2·O_2_, 3·O_2_ and 4·O_2_. After recrystallization from toluene the compounds were isolated as colorless crystalline solids in moderate yields (Fig. 2, top). In the IR spectrum of isolated 2·O_2_, 3·O_2_ and 4·O_2_ the CN stretching frequency at *ca.* 2080 cm^−1^ raised the question whether these compounds are the isocyanide adducts with a P–N≡C, rather than with a P–C≡N connectivity. Geometry optimizations and frequency analyses at the PBE0-D3/def2-SVP DFT level of theory, suggested that the mode at *ca.* 2080 cm^−1^ corresponds to the isocyanide-form (*ν̃*_PNC,calc._ ≈ 2100 cm^−1^). Moreover, the calculated IR spectra of the P–C≡N isomers showed that the C–N stretching modes (*ν̃*_PCN,calc._ ≈ 2210 cm^−1^) are of low intensity and should not be detected in the IR spectrum (*cf.* IR spectrum of 4·O_2_Fig. 2, bottom), in line with the experimental findings. Consequently, the isolated crystals of 2·O_2_, 3·O_2_ and 4·O_2_ were also investigated by Raman spectroscopy using a 633 nm red laser. This clearly showed a low intensity mode at *ca.* 2080 cm^−1^ for the P–NC and a medium intensity mode for the P–CN unit at 2175 cm^−1^ (*cf.*4·O_2_, Fig. 2, bottom), in line with the theoretically predicted Raman spectra of [^Dipp^TerPO_2_(NC)][M(crown)] and [^Dipp^TerPO_2_(CN)][M(crown)], respectively. In the ^31^P NMR spectrum of isolated crystals of 2·O_2_ and 3·O_2_ in C_6_D_6_ two marginally separated singlets in a ratio of *ca.* 1 : 1 are detected at −9.0 and −9.3 ppm (2·O_2_) (*cf.* Fig. S24†) or −8.5 and −9.3 ppm (3·O_2_) (*cf.* Fig. S40†), respectively. The presence of both isomers is further indicated by a splitting of the signals for the ^Dipp^Ter-moiety in the ^1^H and ^13^C NMR spectra, which despite only one ^31^P NMR signal is also observed in 4·O_2_. Variable temperature ^31^P{^1^H} NMR experiments of 4·O_2_ in toluene-d_8_ between −30 and 80 °C clearly show the presence of two species at −30 °C at −8.84 and −8.96 ppm in a nearly 1 : 1 ratio. Coalescence of the two signals is observed at 30 °C accompanied with a gradual downfield-shift of the signal to −8.25 ppm at 80 °C (Fig. S50†). This combined analytical evidence indicates a coordination isomerism at a dioxophosphorane in 2·O_2_, 3·O_2_ and 4·O_2_. We thus conclude that upon oxidation of the P atom the CN^−^ substituent (coordinated to [M(crown)]^+^) can undergo a CN/NC isomerism giving 2·O_2_, 3·O_2_ and 4·O_2_ as a mixture of isomers.

**Fig. 3:**
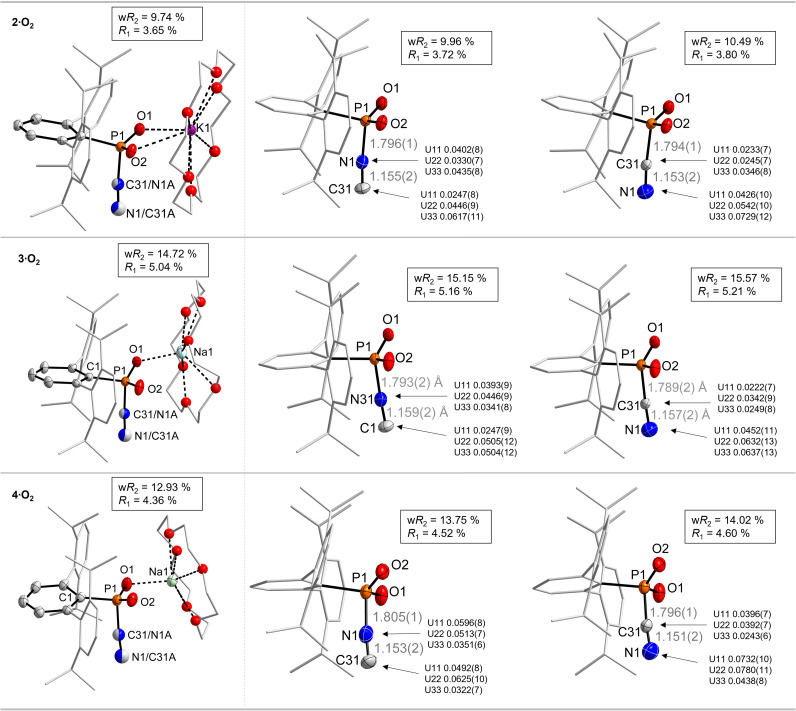
Molecular structure of 2·O_2_, 3·O_2_ and 4·O_2_ (left), with the disorder represented in the form of cake-diagram-type spheres. ORTEP representations (50% probability) of the respective NC- and CN-isomers of 2·O_2_, 3·O_2_ and 4·O_2_ with refinement and anisotropic displacement parameters for each isomer. For crystallographic details also see the ESI.†

2·O_2_, 3·O_2_ and 4·O_2_ all crystallize in the triclinic spacegroup *P*1̄, as their toluene solvates. In all molecular structures the [^Dipp^TerPO_2_(CN)]^−^ anion interacts strongly with the [K(18-c-6)]^+^ counter-cation through two close K⋯O_P_ contacts in 2·O_2_, whereas in 3·O_2_ and 4·O_2_ only one close Na⋯O_P_ contact is detected (Fig. 3). It is difficult to distinguish between the P–CN and P–NC coordination mode in the solid state. Therefore, two structural models were tested in all three cases: (a) with a P−NC unit; (b) with a P–CN unit. The quality of each model was judged by the *R*_1_ and w*R*_2_ values (*cf.* ESI† p. S11), as well as by inspection of the anisotropic displacement parameters (Fig. 3). In both cases the best *R*-values are obtained for the P–NC isomer, however with large displacement parameters for the N atom, which indicates an underlying disorder. This disorder was modelled and revealed approximate 63 : 37 (2·O_2_), 55 : 45 (3·O_2_) and 51 : 49 (4·O_2_) ratios between the P−NC and P–CN form, respectively. This is in line with the observations by ^31^P NMR and vibrational spectroscopy, *vide supra*.

Next, isolated crystals of 2·O_2_ and 3·O_2_, with an apparent CN/NC isomer mixture, were re-dissolved in C_6_D_6_ and heated to 80 °C overnight and the ^31^P NMR spectra after cooling to room temperature clearly showed only one singlet resonance at −9.3 ppm in both cases, while only one set of signals for the Dipp groups were observed in the ^1^H and ^13^C NMR spectra (*cf.* Section 4.4.1 and 4.6.1 of the ESI†). After recrystallization from toluene, Raman spectroscopy only revealed a medium intensity mode at 2175 (2·O_2_, Fig. S28†) or 2176 cm^−1^ (3·O_2_, Fig. S45†), respectively, while no modes were detected in the IR spectra, which is in line with the P–CN isomers.

It can therefore be concluded that the PO_2_(CN) isomer corresponds to the species with the slightly more shielded ^31^P NMR signal in the isomer mixture. When a solid sample of 2·O_2_ is heated to 110 °C for 16 h and is re-dissolved afterwards in C_6_D_6_, only the P–CN isomer with an ^31^P NMR shift of −9.3 ppm is detected (*cf.* Fig. S27†), further corroborated by a ^13^C NMR signal for the P–CN unit at 124.1 ppm (^1^*J*_CP_ = 91 Hz).

Based on experimental evidence this pointed to the P–CN isomer being thermodynamically favored over the P–NC isomer.

The observed isomerization and crystallization of both isomers in the same crystalline matrix raised the question whether 2 could be oxidized in the solid state when exposed to dry O_2_ and if isomerization may occur in the crystalline matrix of 2. Large accessible voids in the solid-state structure should allow diffusion of O_2_ into the crystal lattice. When yellow single crystals of 2 were exposed to O_2_ while being agitated with a stir bar, a color change to light purple was observed in the first 10 minutes.

The purple color then quickly faded and the solid turned into an off-white powder, which, when re-dissolved in C_6_D_6_, clearly showed the presence of an isomer mixture of 2·O_2_ (*cf.* ESI† Section 4.4.3).

**Fig. 4:**
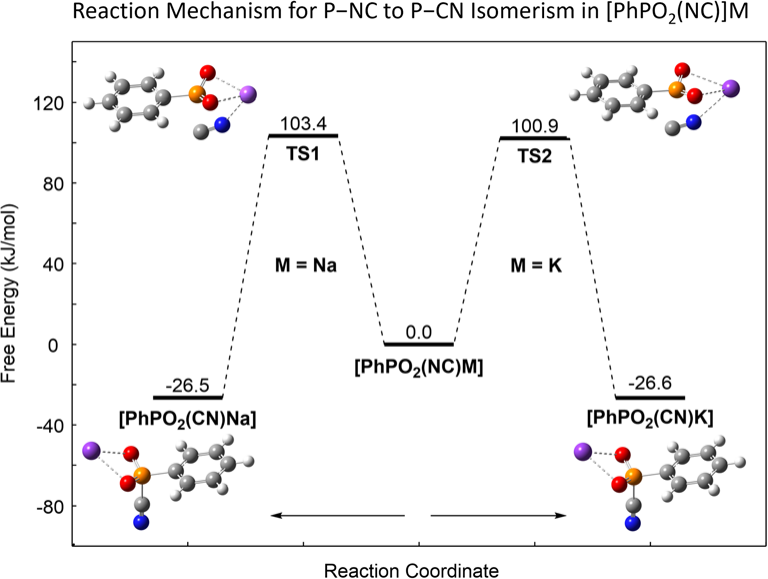
Computed thermal reaction pathway for the NC/CN isomerism in model compounds [PhPO_2_(NC)]M (M = Na, K) (DLPNO-CCSD(T)/def2-TZVP//PBE0-D3/def2TZVP, *c*° = 1 mol L^−1^).

Considering the size of 2·O_2_, 3·O_2_ and 4·O_2_ and overall similar structures, truncated models, namely [PhPO_2_(CN/NC)]M (M = Na, K), were chosen for further theoretical studies with the geometry derived from the molecular structures of 2·O_2_ and 4·O_2_ in the crystal, respectively. These studies at the DLPNO-CCSD(T)/def2-TZVP level of theory,^22,23^ using the PBE0 (ref. 24–26)-D3 (ref. 27 and 28)/def2-TZVP^29^ optimized geometries for obtaining the corrections to the free enthalpy (notation: DLPNO-CCSD(T)/def2-TZVP//PBE0-D3/def2-TZVP; *cf.* ESI† p. S76 ff.), revealed that in both cases the [PhPO_2_(CN)]M isomer is thermodynamically more stable than the isocyanide form by *ca.* 26.5 kJ mol^−1^, in line with the experimentally observed presence of only the P–CN isomer after heating 2 O_2_ and 3·O_2_ in C_6_D_6_ solution, *vide supra*. A transition state that shows N⋯M interactions was found in both cases, clearly pointing to metal ion involvement in the thermal isomerization process. In both cases the barrier is *ca.* 100 kJ mol^−1^ (M = Na, 103.4; M = K, 100.9 kJ mol^−1^) in line with the facile thermal rearrangement at 80 °C in solution and at 110 °C in the solid state (Fig. 4).

As the PO_2_–CN isomer was found to be thermodynamically more stable, the isomerization must occur during the initial oxidation step.

Therefore, the electrochemistry of 3 was exemplarily investigated. CV studies in THF showed an irreversible oxidation event with a peak potential of −0.6 V *vs.* Fc/Fc+ (0.1 M [*n*Bu_4_N]PF_6_), accompanied by the aforementioned color change (Fig. S17†). The oxidation at rather negative potential is in line with reports on the related phosphaethynolate salt Na[PCO] (*E*^ox^ = −0.31 V *vs.* Fc/Fc^+^ in THF).^30^ We therefore propose initial oxidation of the cyanophosphides by ^3^O_2_ to give a neutral P-radical species, which then reacts further with the superoxide anion, to presumably give a linear P–O–O(CN) intermediate. In the final isomerization step the CN-group becomes labile and can bind to the metal-crown cation, and eventually rebound to the P center either as the nitrile or isonitrile.

**Fig. 5:**
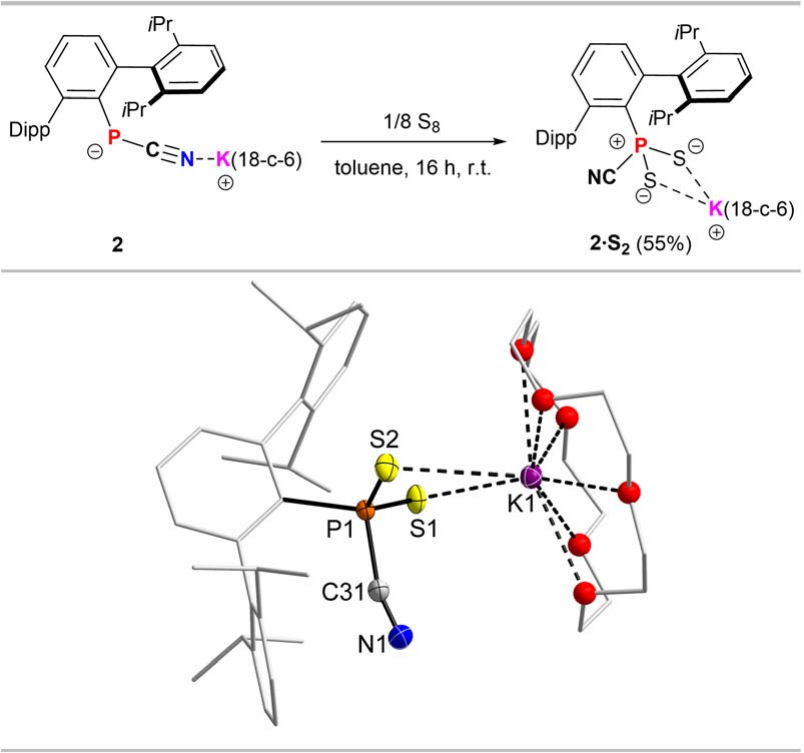
Synthesis of dithiophosphorane cyanide adduct 2·S_2_ (top). Molecular structure of 2·S_2_ (bottom, one of two molecules in the asymmetric unit). Hydrogen atoms omitted and Dipp groups, ethylene bridges in 18-c-6 rendered as wireframe and O atoms rendered as spheres with arbitrary radius for clarity. Thermal ellipsoids are drawn at the 50% probability level. Selected bond lengths (Å) and angles (°): P1–S1 1.9560(5), P1–S2 1.9663(5), P1–C31 1.822(2), N1–C31 1.149(2), S1–K1 3.3707(5), S2–K1 3.3577(5); S1–P1–S2 120.19(2), C1–P1–S1 114.96(4), C1–P1–S2 107.13(4), N1–C31–P1 164.09(14).

The involvement of a radical intermediate in the CN/NC isomerism is further supported by the reaction of 2 with elemental sulphur, which after stirring overnight in toluene afforded [^Dipp^TerPS_2_(CN)][K(18-c-6)] (2·S_2_), with no intermediate color change to violet being observed (Fig. 5, top).

2·S_2_ shows a ^31^P NMR signal in C_6_D_6_ at 32.3 ppm, significantly deshielded when compared to 2·O_2_. A similar deshielding is observed upon oxygen for sulphur exchange from PhPO_2_(IMe_4_) (*δ*(^31^P, CDCl_3_) = 0.8 ppm) to PhPS_2_(IMe_4_) (*δ*(^31^P, CDCl_3_) = 52.9 ppm).^6^ After recrystallization from toluene 2·S_2_ was afforded as a toluene solvate, with no indication of an CN/NC isomer mixture in the solid state (*cf.* ESI† p. S13). This is further supported by only one stretching mode in the Raman spectrum at 2154 cm^−1^ (Fig. S37†), while no mode is observed between 2000–2300 cm^−1^ in the IR spectrum. In the solid state (Fig. 5, bottom) the P–C_CN_ distance in 2·S_2_ (1.822(2) Å) is longer compared to the dioxophosphorane adducts 2·O_2_, 3·O_2_ and 4·O_2_, while the C–N distance is shorter (1.149(2) Å) and in line with a triple bond (*cf.* Σ*r*_cov_(C≡N) = 1.14 Å),^17^ with a P–C–N angle (164.1(1)°) that significantly deviates from linearity, while in 2·O_2_, 3·O_2_ and 4·O_2_ the angles are closer to 180°. Therefore, we assume that the interaction of the cyanophosphides with ^3^O_2_ in the first step facilitates the observed isomerisation.

**Scheme 4:**
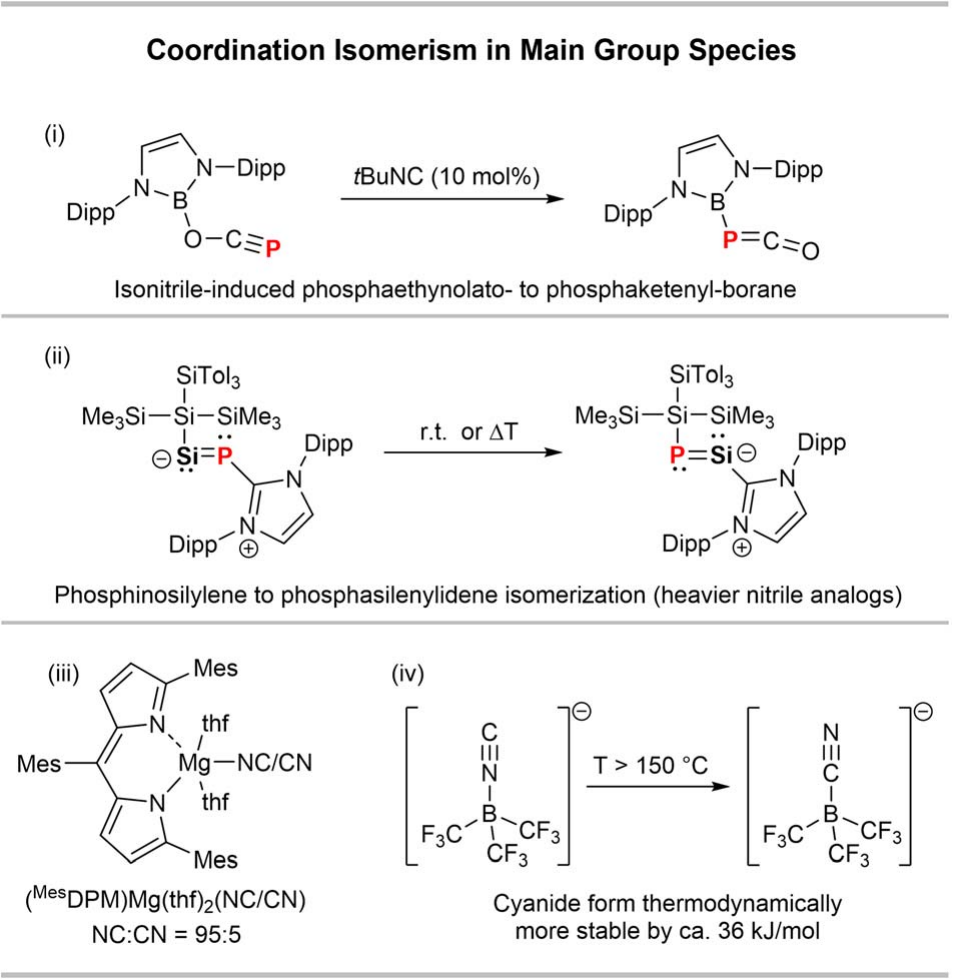
Examples of (i) [B]-PCO/OCP,^31^ (ii) SiP/PSi,^32^ (iii) Mg(CN/NC)^33^ and (iv) B(CN/NC) isomerisms.^34^

Coordination isomerism in main group species is generally rare. Goicoechea and co-workers have lately described a [B]–OCP to [B]–PCO ([B] = (HCNDipp)_2_B) isomerism catalysed by *t*Bu−NC (Scheme 4(i)).^31^ Inoue and co-workers revealed a thermally induced SiP/PSi-isomerism in the heavier nitrile derivative {(SiTol_3_)(SiMe_3_)_2_Si}SiP(IPr) (IPr = (HCNDipp)_2_C) (Scheme 4(ii)).^32^ CN/NC isomerisations in main group species have been observed in magnesium and boron compounds. A MgCN complex with a dipyrromethene ligand (^Mes^DPM, Scheme 4(iii)) was found to mainly exist as the isocyanide MgNC isomer in the solid state with only minor contribution of the MgCN isomer (95 : 5 ratio).^33^ Moreover, it was shown that the borate anion [(F_3_C)_3_B–CN]^−^ is thermodynamically more stable than its [(F_3_C)_3_B–NC]^−^ isomer by 35.2 kJ mol^−1^ (Scheme 4(iv)), as shown experimentally by DSC measurements.^34^

A potential coordination isomerism in the related species PO_2_(CN)(DABCO) (DABCO = 1,4-diazabicyclo(2.2.2)octane) is indicated by ^31^P NMR spectroscopy, while this phenomenon was not further investigated by the authors.^35^ In general, it was shown that the X–CN/X–NC ratio is significantly influenced by the group electronegativity of X, with the X–CN form being favored for more electronegative moieties X.^36–38^ These findings are in line with natural resonance theory (NRT) studies carried out on the model complexes [PhPO_2_(CN/NC)]^−^ (*cf.* ESI† p. S78 ff.), which show that there is a higher covalency in the P–CN isomer with the covalent form being predominant (41.8%) and only minor contribution of a non-bonding ionic form (10.4%). For the P−NC isomer the contribution of the ionic form is considerably higher (27.8%) (Fig. S61,† middle), which is balanced by a lower covalent character (28.8%). This is also reflected in a larger CN to PhPO_2_ charge transfer in the [PhPO_2_(CN)]^−^ form of 0.51*e*^−^ compared to only 0.37*e*^−^ for the isocyanide isomer, with a higher Wiberg bond index (WBI) for the P–CN isomer (0.72) compared to the P−NC isomer (0.58). The P–C_CN_ bond in the cyanide isomer is also considerably less polarized (P: 31.0%; C: 69.0%) compared to the P–N_NC_ bond (P: 21.6%; N: 78.4%), in line with a smaller ionic character in the PCN isomer. Considering the high group electronegativity of the ^Dipp^TerPO_2_ the fact that the cyanide form is thermodynamically stable is in line with previous studies.

### Phosphinidene monoxide cyanide adducts

**Scheme 5:**
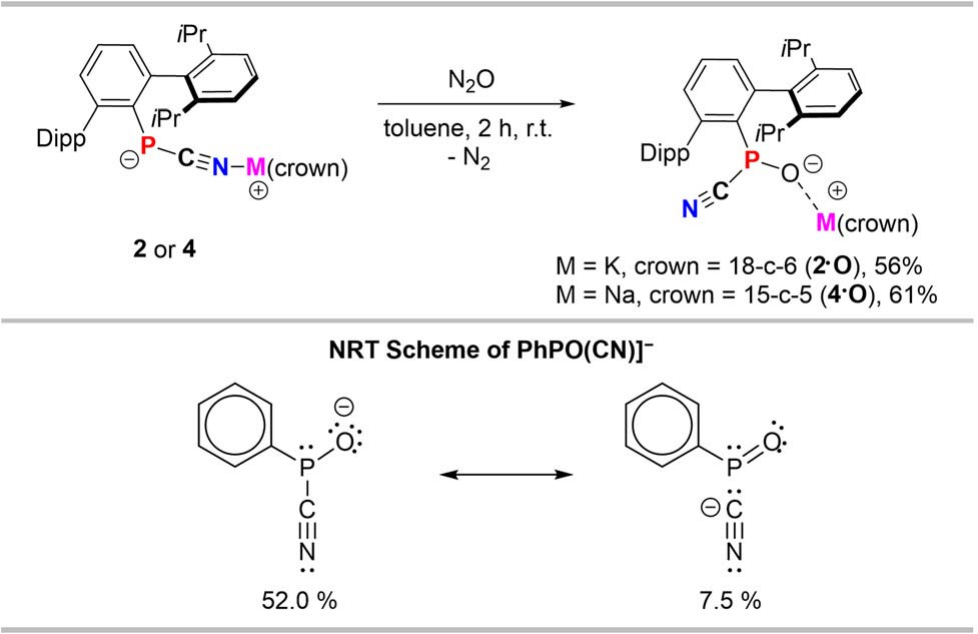
Synthesis of the phosphinidene monoxide species 2·O and 4·O (left) and NRT scheme of the truncated model anion [PhPO(CN)]^−^.

Using N_2_O as an oxidant with isolated 2 or preparing 2*in situ* from 1 and KH in toluene solution, a different product with a significantly deshielded ^31^P NMR signal at 81.1 ppm was obtained (Scheme 5). By comparison with a recent report on the NHC-phosphinidene oxide adduct ^Mes^TerPO(IMe_4_) (*δ*(^31^P) = 89.6 ppm),^7^ this was assigned to the formal anionic phosphinidene oxide cyanide adduct [^Dipp^TerPO(CN)][K(18-c-6)] (2·O), which is further corroborated by the ^31^P NMR shift in the gas phase (*δ*_calc._(^31^P) = 72.8 ppm) obtained at the PBE0-D3/def2-TZVP//PBE0-D3-def2-SVP level of theory (Table S6†). Interestingly, the formation of 2·O is not accompanied by an intermediate color change to purple, further substantiating that the coordination isomerism is related to a radical intermediate.

**Fig. 6:**
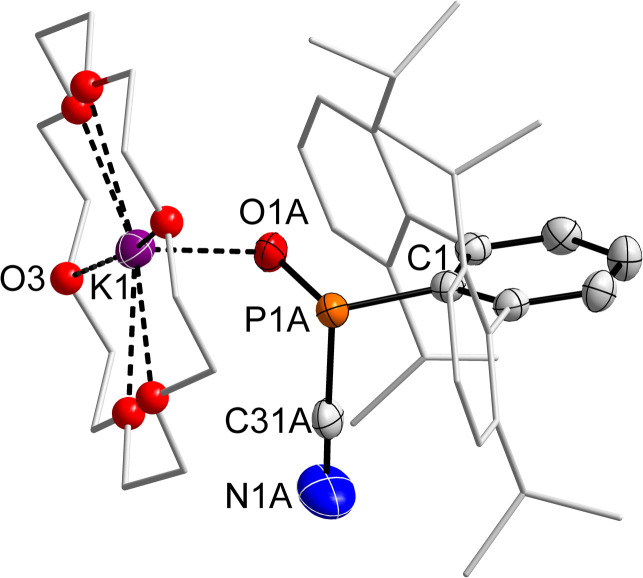
Molecular structure of 2·O. Hydrogen atoms omitted and Dipp groups, ethylene bridges in 18-c-6 rendered as wireframe and O atoms rendered as spheres with arbitrary radius for clarity. Thermal ellipsoids are drawn at the 50% probability level. Selected bond lengths (Å) and angles (°) [values of minor part B]: P1A-C1 1.886(2) [1.860(2)], P1A–C31A 1.882(7) [1.877(9)], C31A–N1A 1.151(8) [1.150(9)], O1A-P1A 1.515(2) [1.528(2)], O1A-K1 2.671(2) [2.632(2)]; P1A–C31A–N1A 166(1) [157(1)], O1A–P1A–C31A–104.2(3) [104.6(8)], O1A–P1A–C1 108.04(10) [106.3(1)], C31A–P1A–C1 93.3(5) [89.9(6)].

To the best of our knowledge 2·O is the only example of an anionic phosphinidene monoxide adduct. Only recently the first free phosphinidene oxide Ar(Bz)N–PO (Ar = 2,6-(3,5-Tipp_2_-C_6_H_3_)_2_–C_6_H_3_; Bz = benzyl) with a deshielded ^31^P NMR signal at *ca.* 285 ppm was isolated using an exceedingly bulky aryl-group.^39^ An unstable neutral phosphine-stabilized cyclic aminophosphinidene oxide was recently reported by Nikonov *et al.*, which showed a similar ^31^P NMR shift (89.6 ppm) compared to 2·O.^9^ SC-XRD quality crystals of 2·O were obtained from a saturated toluene solution (Fig. 6), which revealed the proposed connectivity with a disordered P(O)CN unit, in a nearly 50 : 50 ratio (*cf.* Fig. S5†), which is in line with a stereochemically active lone pair of electrons on the P atom and both enantiomers of 2·O being present in the crystal. Despite the disorder, the P–C_CN_ distance (P1A–C31A 1.882(7), P1B–C31B 1.877(9) Å) is clearly elongated compared to 2 (*cf.* 1.770(1) Å), in line with the description of 2·O as a cyanide adduct of ^Dipp^TerPO. The P–O distance (P1A–O1A 1.515(2), P1B–O1B 1.528(2) Å) is rather long, when compared to the recently reported free phosphinidene oxide R(Bz)N–PO (*cf.* d(P–O) = 1.447(6) Å), but in line with that in the NHC adduct ^Mes^TerPO(IMe_4_) (*cf.* d(P–O) = 1.522(4) Å). The sum of angles at the P atom (*Σ*<(P) = 302.7(4) °) indicates a trigonal pyramidal coordination environment with a short O_P_⋯K contact (2.615(3) Å), rendering this a contact ion pair similar to 2·O_2_ and 4·O_2_. Likewise, 4·O was prepared which showed a minimally deshielded ^31^P NMR signal at 82.9 ppm compared to 2·O. Noteworthy in this case there is a significant broadening and pseudo-doublet form of the 15-c-5 signal in the ^1^H NMR spectrum (*cf.* Fig. S55†). In 2·O and 4·O two sets of signals for the Dipp-substituents of the ^Dipp^Ter-group on P are detected in the ^1^H and ^13^C NMR spectra, respectively, which is similar to starting material 1, and indicates a P center with three different substituents. The ^13^C NMR signal for the P*C*N unit could not be detected, while there is also no mode in the IR spectrum for the CN-group. This is in line with the theoretically predicted spectrum, with no apparent CN/NC isomerism in this case.

Natural resonance theory (NRT) on the truncated model [PhPO(CN)]^−^ showed the zwitterionic form with just single bonds on the three-coordinate phosphorus atom to be the major form (Scheme 4). While minor weight is given to a non-bonding form with a Ph–P=O fragment and a cyanide anion, highlighting the polar character of the P–O bond, in line with a Wiberg bond index (WBI) for the P–CN bond of 0.82 and a significant charge transfer from the CN^−^ fragment to the PhPO-unit of 0.48*e*^−^.

## Conclusions

We have shown that different alkali metal bases (KH, KHMDS, NaHMDS) can be used for the preparation of salts containing the cyanophosphide anion [^Dipp^TerPCN]^−^.

Using chelating crown ethers resulted in structural diversity, showing interactions of the encapsulated cations with the flanking Dipp groups of the ^Dipp^Ter moiety in some cases, while in the case of 3 a rare example of a dimeric [Na_2_(18-c-6)_2_]^2+^ cation was found to bridge two cyanophosphides in a centrosymmetric dimer. Independent of whether 2, 3 or 4 are employed, facile oxidation with dry O_2_ afforded the formal cyanide adducts of the dioxophosphorane ^Dipp^TerPO_2_, as mixtures of cynanide and isocyanide isomers. The coordination isomerism was verified by NMR experiments as well as by vibrational spectroscopy. The cyanide isomer with a PO_2_–CN unit was found to be thermodynamically more stable based on experimental data, supplemented by calculations. These calculations also showed involvement of the alkali metal ion in the isomerisation process. With S_8_ as an oxidant no indication for a coordination isomerism was found, giving only the cyanide adduct of the dithiophosphorane in 2·S_2_. With N_2_O as an oxidant, the anionic phosphinidene oxide adducts 2·O and 4·O were obtained, which show a ^31^P NMR shift in the range expected for this rarely documented class of compounds. The trend previously observed for the stepwise oxidation of P^I^ to P^III^ and eventually P^V^ is observed in this study as well and shows rather shielded P atoms in the ^31^P NMR spectra for anionic P^I^ compound 2 (*δ*(^31^P) = 128.6 ppm), a significant deshielding for 2·O (*δ*(^31^P) = 89.6 ppm), while 2·O_2_ (*δ*(^31^P) = −9.3 ppm) is observed in an intermediate range of the ^31^P NMR chemical shift scale.

Future studies will focus on exploiting the concept of coordination isomerism in P^V^ compounds for the design of new P^V^ based catalyst systems.

## Data availability

The data supporting this article have been included as part of the ESI.† This includes: synthesis and characterization of compounds, NMR spectra, crystallographic, and computational details. Additionally, a cif-file containing the isomer refinements of 2·O_2_, 3·O_2_ and 4·O_2_ (not uploaded to the CCDC) and a multi-structure *xyz*-file containing all optimized geometries have been uploaded. Crystallographic data for 2 (2401210), 3 (2401211), 4 (2401212), 2·O_2_ (2401213), 3·O_2_ (2427596), 4·O_2_ (2401214), 2·S_2_ (2427595), and 2·O (2401215) have been deposited at the CCDC and can be obtained from https://www.ccdc.cam.ac.uk.

## Author contributions

A. A. N. carried out the experimental work and wrote the ESI.† E. Z. carried out parts of the experimental work. F. D. and C. H.-J. were responsible for solving and refining the SCXRD structures. C. H.-J. carried out the quantum chemical calculations. A. P. carried out CV experiments and analysed the data. J. S. carried out the Raman experiments, analysed and visualized the data. C. H.-J. was responsible for the conceptualization, supervision of the experimental investigations, and wrote the initial draft of the manuscript. C. H.-J., F. D. and E. B. finalized the manuscript. All authors agreed to the submitted content.

## Conflicts of interest

There are no conflicts to declare.

## Supplementary Material

SC-016-D4SC07636B-s001

SC-016-D4SC07636B-s002

SC-016-D4SC07636B-s003
