# Supplementary material for: Coordination isomerism in dioxophosphorane cyanides
Source: Chem Sci. 2025 Mar 13;16(16):6909–17. doi: 10.1039/d4sc07636b (PMC11924112; doi:10.1039/d4sc07636b)
Supplement: SC-016-D4SC07636B-s002 [file SC-016-D4SC07636B-s002.pdf]

Supplementary Information (SI) for Chemical Science.
This journal is © The Royal Society of Chemistry 2025

SUPPORTING INFORMATION

Coordination Isomerism in Dioxophosphorane Cyanides

M.Sc. Ayu Afiqah Nasrullah, Dr. Edgar Zander, Dr. Fabian Dankert, Dr. Andrey Petrov, M.Sc. Jonas Surkau, Prof. Dr. Eszter Baráth, Dr. habil. Christian Hering-Junghans

This file includes:

1 Experimental 2
2 Structure elucidation 4
3 Syntheses of starting materials 16
4 Syntheses of compounds 19
5 Computational details 73
6 References 84

1 Experimental

General Information. If not stated otherwise, all manipulations were carried out under oxygen- and moisture-free conditions under an inert atmosphere of argon using standard Schlenk or Drybox techniques. All glassware was heated three times *in vacuo* using a heat gun and cooled under argon atmosphere. Solvents were transferred using syringes, which were purged three times with argon prior to use. Solvents and reactants were either obtained from commercial sources or synthesized as detailed in Table S1.
Caution! When working with PCN-compounds the release of hydrogen cyanide, especially when cleaning glassware, is possible. Therefore, additional safety precautions and a special waste treatment with hydrogen peroxide are recommended.

Table S1: Origin and purification of solvents and reactants.

Substance	Origin	Purification
Toluene	local trade	purified with the Grubbs-type column system "Pure Solv MD-5" and stored under argon over molecular sieves (4 Å)
n-pentane, *n*-hexane	local trade	purified with the Grubbs-type column system "Pure Solv MD-5" and stored under argon over molecular sieves (4 Å)
C_6D_6	Sigma-Aldrich	dried over Na<br>freshly distilled prior to use
Toluene-d_8	Deutero	dried over Na/benzophenone<br>freshly distilled prior to use
KH	ABCR, 30% w/w in mineral oil	Washed 3 times with *n*-hexane prior to use
$Na[N(SiMe_3)_2]$	Sigma Aldrich, 95%	used as received, stored inside a Glovebox
$K[N(SiMe_3)_2]$	Sigma Aldrich, 95%	used as received, stored inside a Glovebox
18-crown-6	EGA	used as received, stored inside a Glovebox
15-crown-5	EGA	sublimation
N_2O	local trade	Passed through a column filled with Sicapent
O_2 (dry air)	local trade	Passed through a column filled with Sicapent
$^{Dip}TerP(PMe_3)$	synthesized	purified according to literature procedure [1]
Sulphur (S_8)	Merck, for synthesis	Used as received, stored inside a Glovebox

NMR spectra were recorded on Bruker spectrometers (AVANCE 300, AVANCE 400 or Fourier 300) and were referenced internally to the deuterated solvent (^{13}C: C_6D_6 δ_{ref} = 128.06 ppm, C_7D_8 δ_{ref} = 20.40 ppm), to protic impurities in the deuterated solvent (^{1}H: C_6HD_5 δ_{ref} = 7.16 ppm, C_7D_8 δ_{ref} = 2.09 ppm) or externally (^{31}P: 85% H_3PO_4 δ_{ref} = 0 ppm). All measurements were carried out at ambient temperature unless denoted otherwise. NMR signals were assigned using experimental data (e.g. chemical shifts, coupling constants, integrals where applicable).

IR spectra of crystalline samples were recorded on a Bruker Alpha II FT-IR spectrometer equipped with an ATR unit at ambient temperature under an argon atmosphere. Relative intensities are reported according to the following intervals: very weak (vw, 0–10%), weak (w, 10–30%), medium (m, 30–60%), strong (s, 60–90%), very strong (vs, 90–100%).

Raman spectra of crystalline sample of **2·O₂**, **2·S₂**, **3·O₂**, **4·O₂**, and **2·O₂**, **3·O₂** (after heating in solution at 80 °C) were recorded using a LabRAM HR 800 Horiba Jobin YVON Raman spectrometer equipped with an Olympus BX41 microscope with variable lenses. The samples were excited by a red laser (633 nm, 17 mW, air-cooled HeNe laser). Relative intensities are reported according to a numerical scale from 1−10.

Elemental analyses were obtained using a Leco TruSpec Micro CHNS Elemental Analysator.

Mass spectra were recorded on a Thermo Electron MAT 95-XP sector field mass spectrometer using crystalline samples in CI mode. HRMS data were obtained on an Agilent 1260/6130 Quadrupol LC-MS mass spectrometer with ESI ionization method.

Cyclic voltammetry: Recorded using a Metrohm Autolab PGSTAT204 potentiostat. The gas-tight glass CV cell was equipped with a glassy carbon disk working electrode, a platinum disk as counter electrode and a leak-free Ag/AgCl reference electrode. CVs were recorded at room temperature in 0.1 M [nBu$_4$N]PF$_6$ solution in THF (10 mL) under an argon atmosphere. Internal standard: Ferrocene. Scan rate: 100 mV/s.

2 Structure elucidation

X-ray Structure Determination: X-ray quality crystals were selected in Fomblin YR-1800 perfluoroether (Alfa Aesar) at ambient temperature. The samples were cooled to 110(2) K (**2**) or 150(2) K during measurement. The data were collected on a STOE IPDS II diffractometer or on a Bruker Kappa Apex II diffractometer using either Mo K_α (λ = 0.71073 Å) or Cu K_α (λ = 1.54184 Å) (**3**, **2·O₂**, **2·S₂**). The structures were solved by iterative methods (SHELXT)[2] and refined by full matrix least squares procedures (SHELXL).[3] Semi-empirical absorption corrections (multiscan and additional spherical absorption correction) were applied to the diffraction data recorded with the STOE device using the LANA application within the STOE X-AREA platform.[4] Semi-empirical absorption corrections (multi-scan and additional spherical absorption correction) were applied to the diffraction data collected with the Bruker device using the SADABS application within the APEX II platform.[5] All non-hydrogen atoms were refined anisotropically, hydrogen atoms were included in the refinement at calculated positions using a riding model.

3: The compound crystallizes with two crystallographically independent molecular entities in the asymmetric unit. A monomeric structural motif as well as a dimeric motif are observed.

The *monomeric* part shows disorder around the crown-ether due to different orientations of the ethylene bridges and oxygen donors. To resolve the disorder, several SADI, ISOR and DELU restraints were employed. The split positions were allowed to refine freely using the FVAR instruction. According to FVAR3 the occupancies for the split positions refine to 0.55 and 0.45. The *dimeric* part shows severe disorder of the crown-ether moiety through rotation around the sodium ion. To fix the disorder, an initial position of the crown ether was refined and then used as a template to model a split position using the OLEX2 implemented SPLIT SAME

module.[6] Split positions for the crown ether were subsequently refined employing several SADI, ISOR and DELU restraints and one EADP (C79 > C79A) constraint. The split positions were allowed to refine freely using the FVAR instruction. According to FVAR2 the occupancies for the split positions refine to 0.54 and 0.46. The C60-62 *i*Pr-group showed disorder through tilting. Accordingly, it was split in two parts employing SADI, ISOR and DELU restraints. The occupancy of each part was allowed to refine freely using the FVAR instruction. According to FVAR4 the occupancies for the split positions refine to 0.74 and 0.26. Co-crystalline solvent (toluene) was treated with a solvent mask (PLATON/SQUEEZE).

4: The C13-15 *i*Pr-group was found to be disordered and was split in two parts, the occupancy of each part was allowed to refine freely. Part of one of the 15-c-5 molecules was found to be disordered and was split in two parts and the occupancy of each part was allowed to refine freely.

2·O_2: The C44-C50 toluene was found to be disordered and were split in two parts, the occupancy of each part was allowed to refine freely. The geometries of both of each part were restrained to be equal using the SAME command. A potential NC/CN-disorder was not resolved and solutions for both isomers are provided as additional cif-file (not uploaded to the CCDC).

Due to the ambiguity of both refinement models RPCN / RPNC, the CN/NC moiety was described with an EXYZ constraint. As such, C and N share the same position in the crystal and have been treated with an EADP command to add equivalent ADPs for the C/N atoms sharing one site. The two positions refined with EXYZ were allowed to refine freely using the FVAR instruction. Improved R-values were obtained with this approach (R_1 = 3.65 %; wR_2 = 9.74 %).

2·S$_2$: The compound crystallizes with two independent molecules in the asymmetric unit as its toluene solvate. Three of four independently to refine toluene molecules are disordered. Whereas it was sufficient to employ a single split position for two of the disordered toluene entities and allowing the respective atoms to refine freely (FVAR2/3 final occupancies 0.60/0.40//0.61/0.39), the third one has several possible split positions. Three possible positions have been refined using the SUMP instruction. According to the FVAR4/5/6, occupancies for these positions refined freely to 0.55/0.24/0.21. For all disordered toluene entities, several restraints including SADI, ISOR and DELU were used.

We note again, that no coordination isomerism can be observed.

3·O$_2$: The compound crystallizes with two independent molecules in the asymmetric unit as its toluene solvate. One isopropyl group of one of the ^{Dip}Ter ligands is slightly disordered and was thus refined employing split positions. Allowing to refine the respective atoms (C71-73; C109-111) freely according to FVAR6 gives occupancies of 0.8 and 0.2, respectively. Two of four independently to refine toluene molecules are disordered. Whereas it was sufficient to employ a single split position for one of the disordered toluene entities (C130–136; C137–143) and allowing the respective atoms to refine freely (FVAR2; final occupancies 0.45/0.55), the second one has several possible split positions. Three possible positions have been refined using the SUMP instruction decreasing significantly the surrounding residual electron density. According to the FVAR3/4/5, occupancies for these positions refined freely to 0.63/0.15/0.22. For all disordered toluene as well as the mentioned isopropyl group, several restraints including SADI, ISOR and DELU were used.

A potential NC/CN-disorder (both independent molecules) was not resolved and solutions for both isomers are provided as additional cif-file (not uploaded to the CCDC).

Due to the ambiguity of both refinement models RPCN / RPNC (both independent molecules), the CN/NC moiety was described with an EXYZ constraint each. As such, C

and N share the same position in the crystal and have been treated with an EADP command to add equivalent ADPs for the C/N atoms sharing one site. All positions refined with EXYZ were allowed to refine freely using the FVAR7/8 instruction (final occupancies: 0.45(CN)/0.55(NC)//0.43(CN)/0.57(NC)). Improved *R*-values were obtained with this approach (R_1 = 5.04 %; wR_2 = 14.72 %).

4·O_2: One of the toluene molecules was found to be disordered and was split in two parts, the occupancy of each part was allowed to refine freely. The 15-c-5 molecules was found to be disordered and was split in two parts and the occupancy of each part was allowed to refine freely. A potential NC/CN-disorder was not resolved and solutions for both isomers are provided as additional cif-file (not uploaded to the CCDC).

Due to the ambiguity of both refinement models RPCN / RPNC, the CN/NC moiety was described with an EXYZ constraint. As such, C and N share the same position in the crystal and have been treated with an EADP command to add equivalent ADPs for the C/N atoms sharing one site. The two positions refined with EXYZ were allowed to refine freely using the FVAR instruction. Improved R-values were obtained with this approach (R_1 = 4.36 %; wR_2 = 12.93 %).

2·O: The OPCN-unit was found to be disordered and was split in two parts, the occupancy of each part was allowed to refine freely. The asymmetric unit contains two disordered toluene molecules, which split in two or three parts, respectively, and the occupations of each part were allowed to refine freely.

Table S2: Crystallographic details of **2**, **3** and **4**.

Compound	**2**	**3**	**4**
Chem. Formula	$C_{43}H_{61}KNO_6P \cdot 2\ C_7H_8$	$2 \cdot C_{43}H_{61}NaNO_6P$	$C_{41}H_{57}NNaO_5 \cdot 0.5\ C_7H_8$
Formula weight [g/mol]	942.26	1482.76	743.90
Colour	yellow	yellow	yellow
Crystal system	triclinic	triclinic	triclinic
Radiation	$Mo_{K\alpha}$, 0.71073	$Cu_{K\alpha}$, 1.54184	$Mo_{K\alpha}$, 0.71073
Space group	$P\bar{1}$	$P\bar{1}$	$P\bar{1}$
a [Å]	13.0425(11)	14.5215(4)	14.468(3)
b [Å]	14.2978(12)	18.3696(5)	17.705(4)
c [Å]	15.5953(13)	20.0346(6)	18.868(4)
α [°]	95.495(2)	82.9090(10)	70.73(3)
β [°]	99.469(2)	83.6580(10)	77.25(3)
γ [°]	108.484(2)	77.7760(10)	75.49(3)
V [Å^3]	2686.4(4)	5163.6(3)	4366.4(19)
Z	2	2	4
$\rho_{calcd.}$ [g/cm^3]	1.165	0.951	1.132
μ [mm^{-1}]	0.177	0.846	0.115
T [K]	110(2)	150(2)	150(2)
θ range for collection	1.341 – 29.999	2.231 – 66.690°	1.728 – 29.323°
Measured reflections	181181	68325	50613
Independent reflections	15673	18231	23409
Reflections with $I > 2\sigma(I)$	12735	15856	13837
R_{int}	0.0396	0.0319	0.0354
F(000)	1016	1600	1604
$R_1(R[F^2>2\sigma(F^2)])$	0.0398	0.0565	0.0527
$wR_2(F^2)$	0.1103	0.1684	0.1581
GooF	1.015	1.040	0.971
No. of Parameters	605	1195	1078
CCDC #	2401210	2401211	2401212

Table S3: Crystallographic details of **2·O₂**, **2·S₂** and **3·O₂**.

Compound	**2·O_2**	**2·S_2**	**3·O_2**
Chem. Formula	$C_{43}H_{61}KNO_8P \cdot 2\ C_7H_8$	$C_{43}H_{61}KNO_6S_2P \cdot 2\ C_7H_8$	$C_{43}H_{61}NaNO_8P \cdot 2\ C_7H_8$
Formula weight [g/mol]	974.26	1006.46	958.15
Colour	colourless	colourless	colourless
Crystal system	triclinic	monoclinic	monoclinic
Radiation	$Cu_{K\alpha}$, 1.54184	$Cu_{K\alpha}$, 1.54184	$Mo_{K\alpha}$, 0.71073
Space group	$P\bar{1}$	$P2_1/n$	$P\bar{1}$
a [Å]	10.4129(4)	12.4061(7)	10.4814(6)
b [Å]	14.6924(6)	40.667(2)	20.0122(12)
c [Å]	19.2865(8)	22.5868(13)	26.3567(15)
α [°]	98.680(2)	90	91.519(2)
β [°]	90.422(2)	91.183(3)	99.269(2)
γ [°]	110.288(2)	90	99.707(2)
V [Å^3]	2730.22(19)	11393.0(11)	5370.0(5)
Z	2	8	4
$\rho_{calcd.}$ [g/cm^3]	1.185	1.174	1.185
μ [mm^{-1}]	1.543	2.134	0.112
T [K]	150(2)	150(2)	150(2)
θ range for collection	2.322 – 66.630	2.173 – 66.840	1.034 – 28.758
Measured reflections	55387	118615	278986
Independent reflections	9646	20120	27880
Reflections with $I > 2\sigma(I)$	8242	18092	19778
R_{int}	0.0482	0.0433	0.0514
F(000)	1048	4320	2064
$R_1(R[F^2>2\sigma(F^2)])$	0.0365	0.0328	0.0504
$wR_2(F^2)$	0.0974	0.0912	0.1472
GooF	1.022	1.041	1.026
No. of Parameters	683	1422	1565
CCDC #	2401213	2427595	2427596

Table S4: Crystallographic details of **4·O$_2$** and **2·O**.

Compound	**4·O$_2$**	**2·O**
Chem. Formula	$C_{41}H_{57}NaNO_8P \cdot 2\ C_7H_8$	$C_{43}H_{61}NaNO_7P \cdot 2\ C_7H_8$
Formula weight [g/mol]	914.10	773.99
Colour	colourless	yellow
Crystal system	triclinic	monoclinic
Radiation	$Mo_{K\alpha}$, 0.71073	$Mo_{K\alpha}$, 0.71073
Space group	$P\bar{1}$	$P2_1/c$
a [Å]	12.760(3)	10.4542(4)
b [Å]	12.763(3)	20.5805(5)
c [Å]	16.898(3)	25.3528(9)
α [°]	92.40(3)	90
β [°]	107.37(3)	92.771(3)
γ [°]	91.81(3)	90
V [Å^3]	2621.4(10)	5448.3(3)
Z	2	4
$\rho_{calcd.}$ [g/cm^3]	1.158	1.168
μ [mm^{-1}]	0.111	0.177
T [K]	150(2)	150(2)
θ range for collection	1.599 – 29.388	1.608 – 29.241°
Measured reflections	43845	104995
Independent reflections	14197	14748
Reflections with $I > 2\sigma(I)$	9792	9319
R_{int}	0.0236	0.0519
F(000)	984	2064
$R_1(R[F^2>2\sigma(F^2)])$	0.0436	0.0407
w$R_2(F^2)$	0.1293	0.1115
GooF	1.082	0.943
No. of Parameters	795	805
CCDC #	2401214	2401215

Refinement of **2·O_2**, **3·O_2** and **4·O_2** was first attempted with solely a P–CN or P–NC moiety, respectively. The following table shows the differences in the refinement parameters and clearly shows that the refinement that takes the positional CN/NC-disorder into account shows the best fit. This is further illustrated in Figure S1, in which the thermal extinction parameters of the ellipsoids in each of the isomers are compared.

	**2·O_2 (disorder)**	**2·O_2 (P–CN)**	**2·O_2 (P–NC)**
$R_1(R[F^2>2\sigma(F^2)])$	0.0365	0.0380	0.0372
$wR_2(F^2)$	0.0974	0.1049	0.0996
GooF	1.022	1.015	1.022
No. of Parameters	683	682	683
	**3·O_2 (disorder)**	**3·O_2 (P–CN)**	**3·O_2 (P–NC)**
$R_1(R[F^2>2\sigma(F^2)])$	0.0504	0.0521	0.516
$wR_2(F^2)$	0.1472	0.1557	0.1515
GooF	1.026	1.031	1.027
No. of Parameters	1565	1563	1563
	**4·O_2 (disorder)**	**4·O_2 (P–CN)**	**4·O_2 (P–NC)**
$R_1(R[F^2>2\sigma(F^2)])$	0.0436	0.0460	0.0452
$wR_2(F^2)$	0.1293	0.1402	0.1375
GooF	1.082	1.072	1.073
No. of Parameters	795	794	795

Figure S1: Molecular structures of **2·O₂** (top), **3·O₂** (middle) and **4·O₂** (bottom). ORTEP representations (50 % probability) of the final refinement using a disorder model (left) or refined as the respective NC-(middle) or CN-isomers (right) with refinement and anisotropic displacement parameters for each isomer.

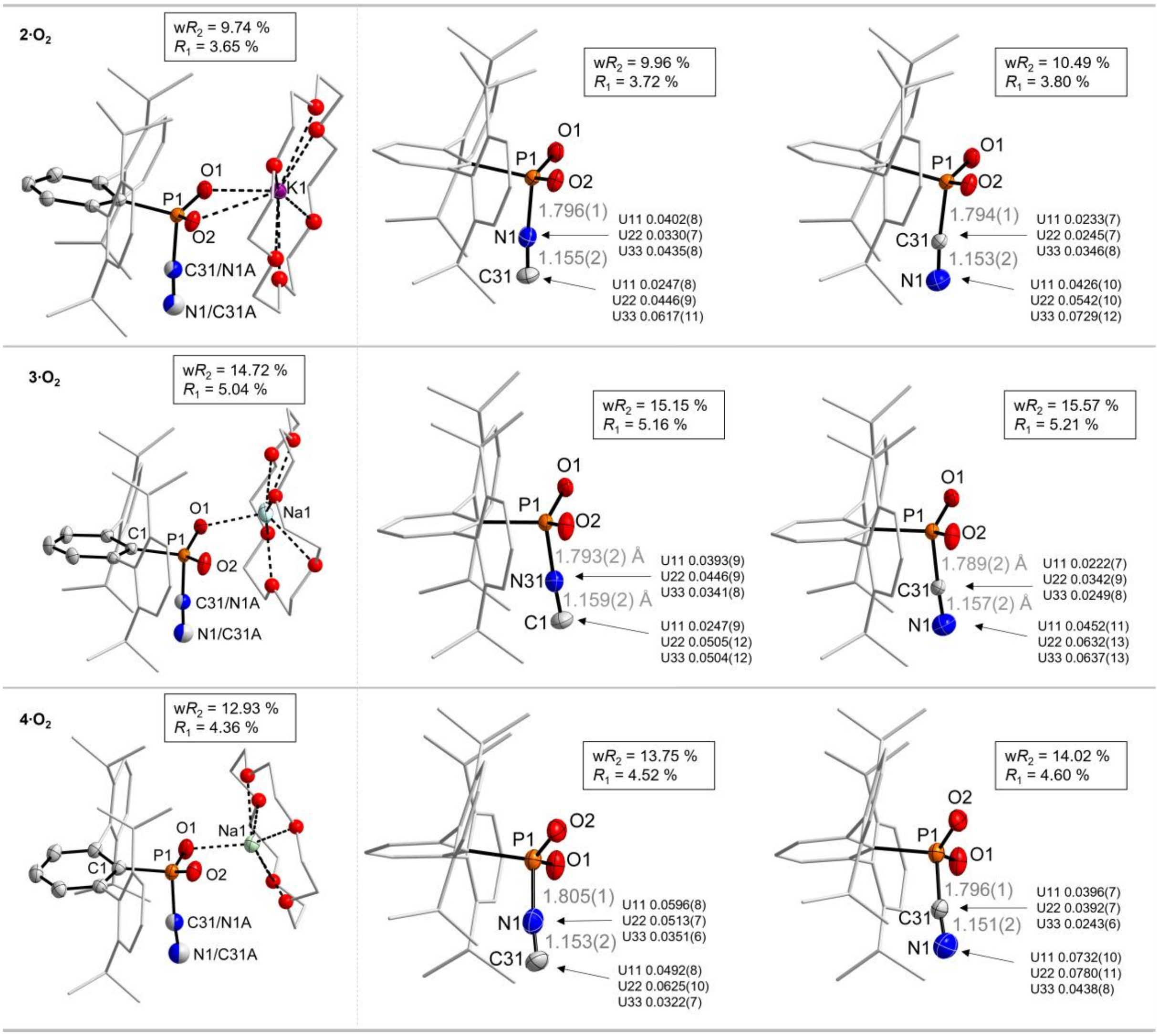

Refinement of **2·S₂** was first attempted with solely a P−CN or P−NC moiety, respectively, clearly showing that the P−CN isomer gives considerably better refinement parameters. The following table shows the differences in the refinement parameters and clearly shows that the P−CN refinement shows the best fit. This is further illustrated in Figure S1, in which the thermal extinction parameters of the ellipsoids in each of the isomers are compared.

	**2·S$_2$ (P−CN)**	**2·S$_2$ (P−NC)**
$R_1(R[F^2>2\sigma(F^2)])$	0.0328	0.0368
w$R_2(F^2)$	0.0912	0.1047
GooF	1.034	1.041
No. of Parameters	1422	1422

Figure S2: Molecular structuree of **2·S$_2$**. ORTEP representations (50 % probability) of the final refinement as the respective CN- (left) or NC-isomer (right) with refinement and anisotropic displacement parameters for each isomer. Counter-cation [K(18-crown-6]$^+$ omitted for clarity.

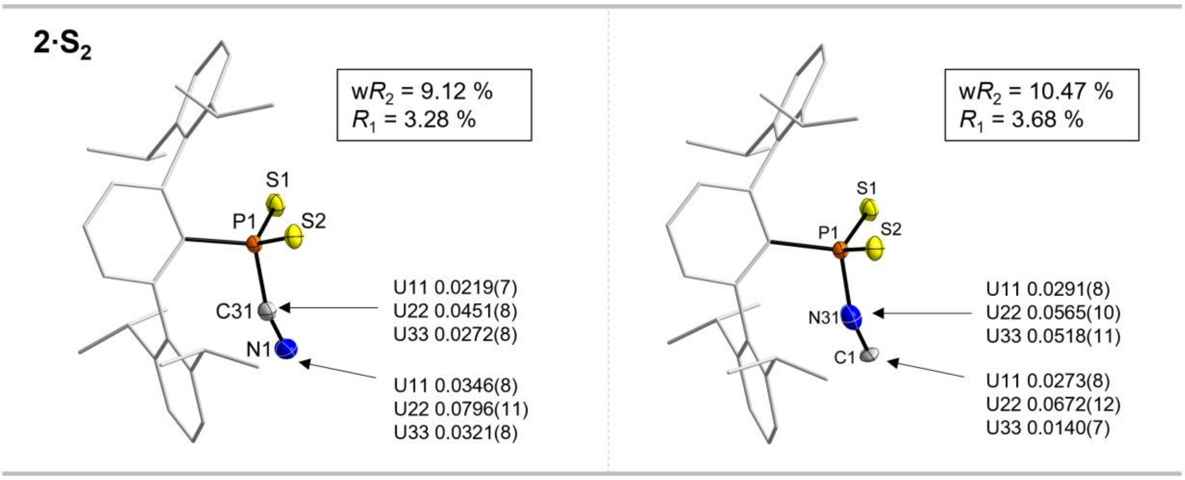

Figure S3: Molecular structure of the two independent entities in the unit cell of **3**, a monomeric form (top) and centrosymmetric dimer (bottom). Hydrogen atoms omitted; Dipp groups, ethylene bridges in 18-c-6 rendered as wireframe and O atoms of 18-c-6 rendered as spheres with arbitrary diameter for clarity reasons. Thermal ellipsoids are drawn at the 50% probability level. Selected bond lengths (Å) and angles (°): P1−C1 1.767(2), P1−C2 1.821(2), N1−C1 1.164(3), Na1−N1 2.388(2), P2−C44 1.761(2), P2−C45 1.819(2), N2−C44 1.160(3), N2−Na2 2.417(2), Na2−O11′ 2.609(3); C1−P1−C2 106.36(8), N1−C1−P1 166.44(17), C1−N1−Na1 171.71(2), C44−P2−C45 105.31(8), N2−C44−P2 165.74(2), C44−N2−Na2 146.1(2), N2−Na2−O11′ 165.97(8) (′ symmetry generated: 1-x,1-y,1-z).

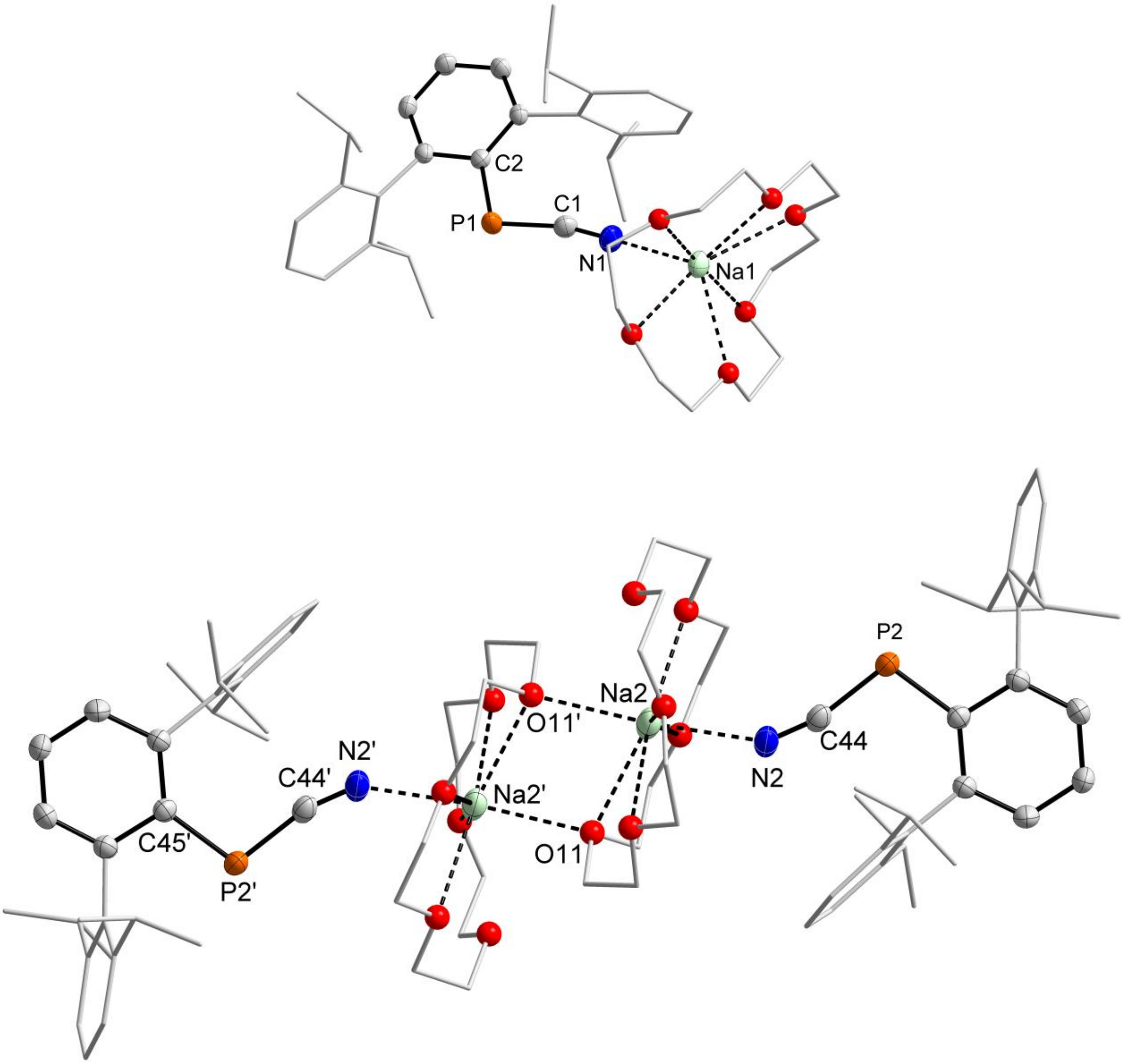

Figure S4: Molecular structure of the two independent entities in the unit cell of **3**, a monomeric form (top) and centrosymmetric dimer (bottom). Hydrogen atoms omitted; Dipp groups, ethylene bridges in 18-c-6 rendered as wireframe and O atoms of 18-c-6 rendered as spheres with arbitrary diameter for clarity reasons. Thermal ellipsoids are drawn at the 50% probability level. Selected bond lengths (Å) and angles (°): P1−C1 1.767(2), P1−C2 1.821(2), N1−C1 1.164(3), Na1−N1 2.388(2), P2−C44 1.761(2), P2−C45 1.819(2), N2−C44 1.160(3), N2−Na2 2.417(2), Na2−O11′ 2.609(3); C1−P1−C2 106.36(8), N1−C1−P1 166.44(17), C1−N1−Na1 171.71(2), C44−P2−C45 105.31(8), N2−C44−P2 165.74(2), C44−N2−Na2 146.1(2), N2−Na2−O11′ 165.97(8) (′ symmetry generated: 1-x,1-y,1-z).

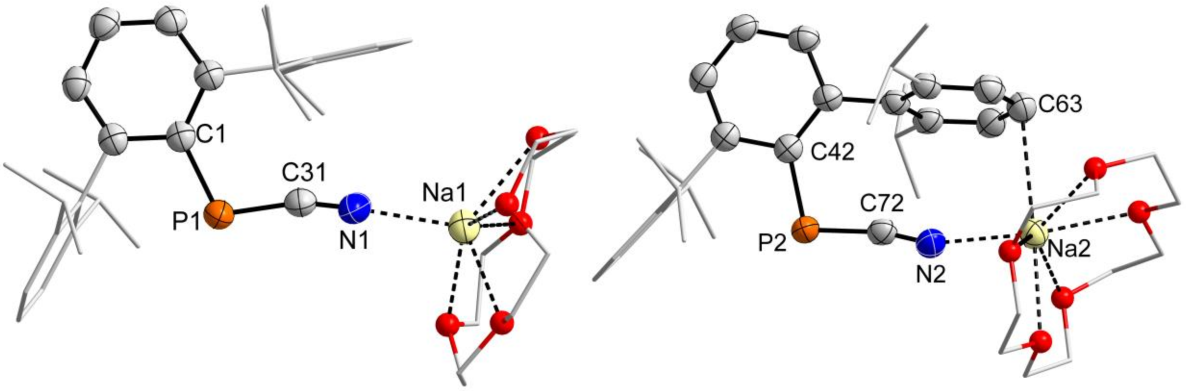

Figure S5: Two views of the molecular structure of **2·O**. Hydrogen atoms omitted and Dipp groups, ethylene bridges in 18-c-6 rendered as wireframe and O atoms rendered as spheres with arbitrary radius for clarity. Thermal ellipsoids are drawn at the 50% probability level. Selected bond lengths (Å) and angles (°) [values of minor part B]: P1A-C1 1.886(2) [1.860(2)], P1A−C31A 1.882(7) [1.877(9)], C31A−N1A 1.151(8) [1.150(9)], O1A-P1A 1.515(2) [1.528(2)], O1A-K1 2.671(2) [2.632(2)]; P1A−C31A−N1A 166(1) [157(1)], O1A−P1A−C31A−104.2(3) [104.6(8)], O1A−P1A−C1 108.04(10) [106.3(1)], C31A−P1A−C1 93.3(5) [89.9(6)].

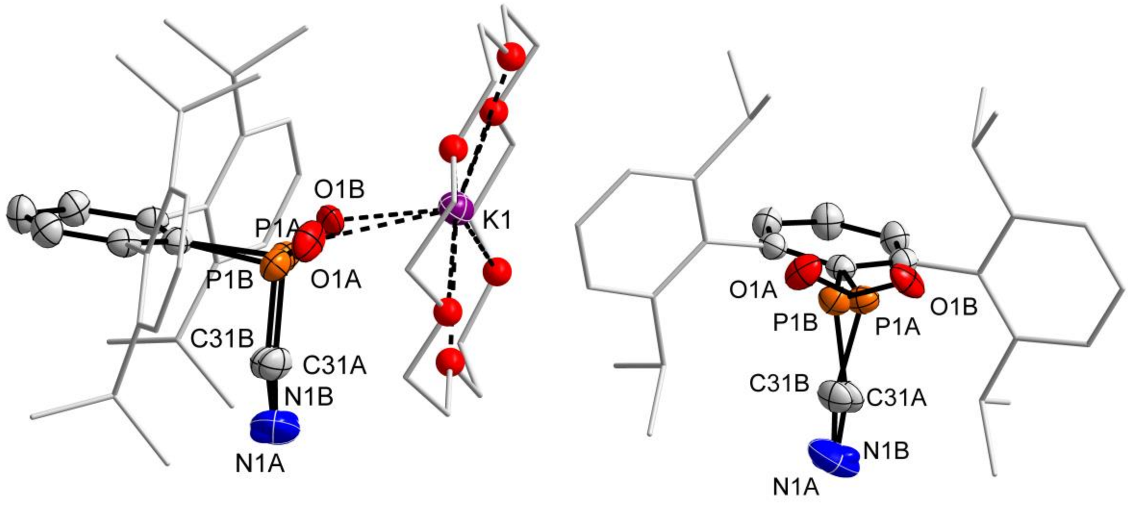

3 Syntheses of starting materials

3.1 ^{Dipp}TerP(H)CN (1)

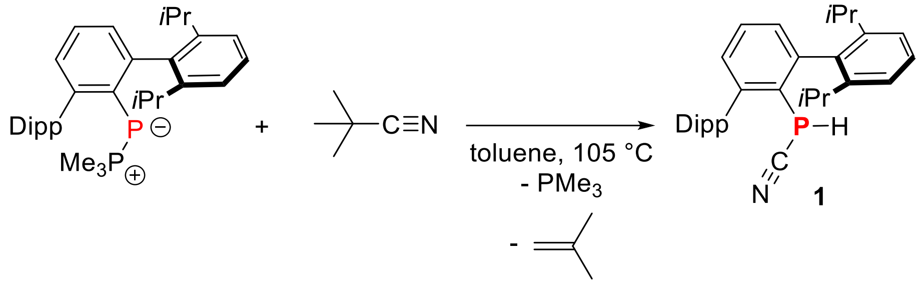

^{Dipp}TerP(H)CN *was prepared according to a slightly modified literature procedure.*[7] ^{Dipp}TerPPMe$_3$ (1.0 g, 2.0 mmol) was dissolved in 25 mL of toluene and *tert*-butyl isocyanide (0.8 mL, 7.1 mmol) was added slowly and afterwards the mixture was subjected to three freeze-pump-thaw cycles. The reaction mixture was then stirred for four days at 105 °C (oil bath). Then, all volatile components were removed under vacuum and the residue was washed with small amounts of *n*-pentane (3 x 1 mL). Subsequent drying under vacuum yielded ^{Dipp}TerP(H)CN (**1**) as off-white powder. Yield: 0.77 g (1.69 mmol, 84 %).

^{31}P{^{1}H} NMR (C_6D_6, 121.5 MHz): δ = −120.4 **^{1}H NMR** (C_6D_6, 300.1 MHz): δ = 0.99 (d, $^{3}J(^{1}H,^{1}H)$ = 6.7 Hz, 6 H, CH(CH_3)$_2$), 1.00 (d, $^{3}J(^{1}H,^{1}H)$ = 6.7 Hz, 6 H, CH(CH_3)$_2$), 1.29 (d, $^{3}J(^{1}H,^{1}H)$ = 6.7 Hz, 6 H, CH(CH_3)$_2$), 1.35 (d, $^{3}J(^{1}H,^{1}H)$ = 6.7 Hz, 6 H, CH(CH_3)$_2$), 2.57 (sept, $^{3}J(^{1}H,^{1}H)$ = 6.7 Hz, 2 H, C*H*(CH$_3$)$_2$), 2.72 (sept, $^{3}J(^{1}H,^{1}H)$ = 6.7 Hz, 2 H, C*H*(CH$_3$)$_2$), 7.07-7.10 (m, 3 H, *m*-CH_{DipTer}, *p*-CH_{DipTer}), 7.14-7.20 (m, 4 H, *m*-CH_{Dip})*, 7.29 (t, $^{3}J(^{1}H,^{1}H)$ = 7.6 Hz, 2 H, *p*-CH_{Dip}); * overlap with C_6D_5H signal. **^{13}C{^{1}H} NMR** (C_6D_6, 75.5 MHz): δ = 23.2 (d, $J_{P,C}$ = 2.5 Hz, CH(CH$_3$)$_2$), 23.4 (d, $J_{P,C}$ = 1.9 Hz, CH(CH$_3$)$_2$), 25.6 (s, CH(CH$_3$)$_2$), 25.8 (s, CH(CH$_3$)$_2$), 31.3 (s, *C*H(CH$_3$)$_2$), 31.4 (d, $J_{P,C}$ = 1.7 Hz, *C*H(CH$_3$)$_2$), 116.6 (d, $^{1}J_{P,C}$ = 75.7 Hz, PC≡N), 123.7 (d, $J_{P,C}$ = 3.9 Hz, CH$_{Aryl}$), 126.5 (d, $J_{C,P}$ = 4.7 Hz, C$_{q,Aryl}$),

129.3 (s, CH_{Aryl}), 129.7 (d, $J_{P,C}$ = 3.6 Hz, CH_{Aryl}), 130.3 (d, $J_{P,C}$ = 2.3 Hz, CH_{Aryl}), 138.2 (d, $J_{P,C}$ = 3.3 Hz, $C_{q,Aryl}$), 146.6 (d, $J_{P,C}$ = 14.1 Hz, $C_{q,Aryl}$), 146.8 (d, $^{1}J_{P,C}$ = 61.3 Hz, $C_{q}P$).

Figure S6: ^{1}H NMR spectrum of **^{Dipp}TerP(H)CN** (300.1 MHz, C_6D_6, rt).

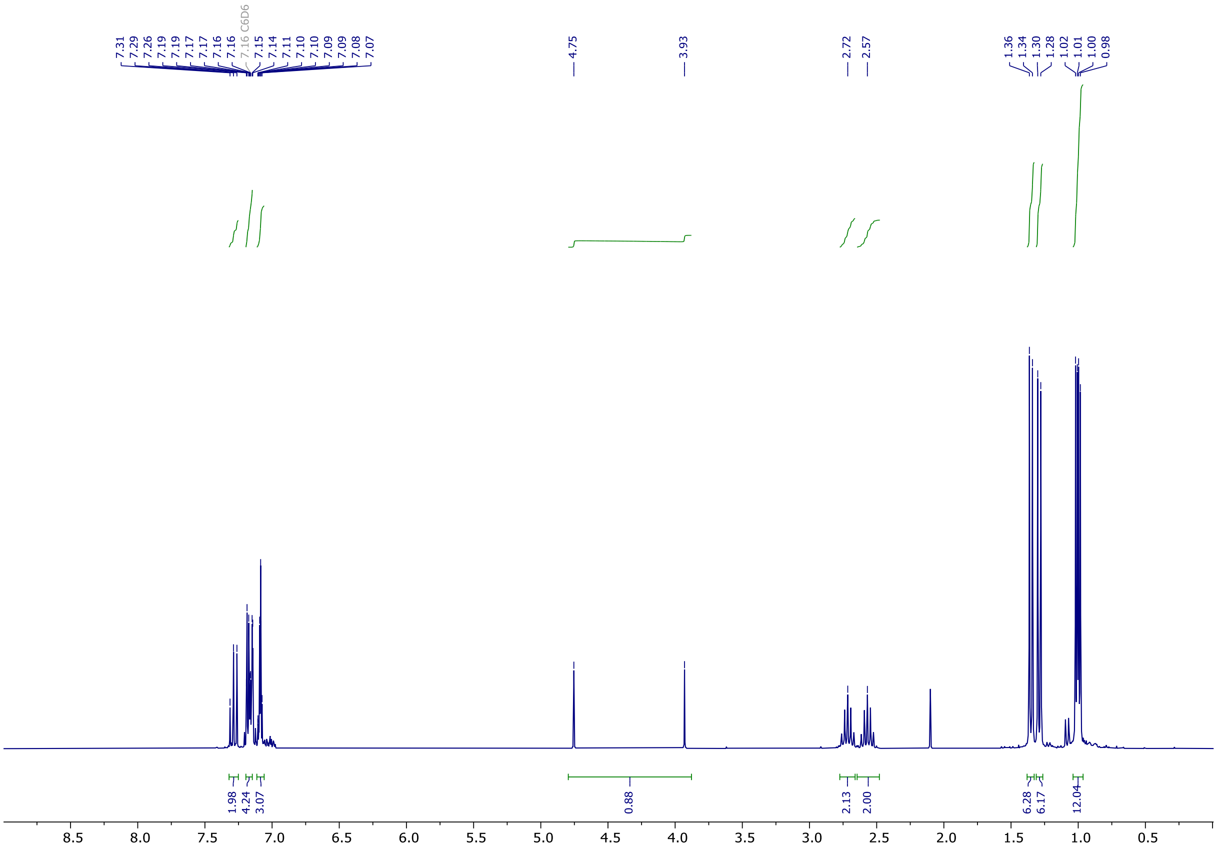

Figure S7: $^{13}C\{^{1}H\}$ NMR spectrum of ^{Dipp}**TerP(H)CN** (75.5 MHz, C_6D_6, rt).

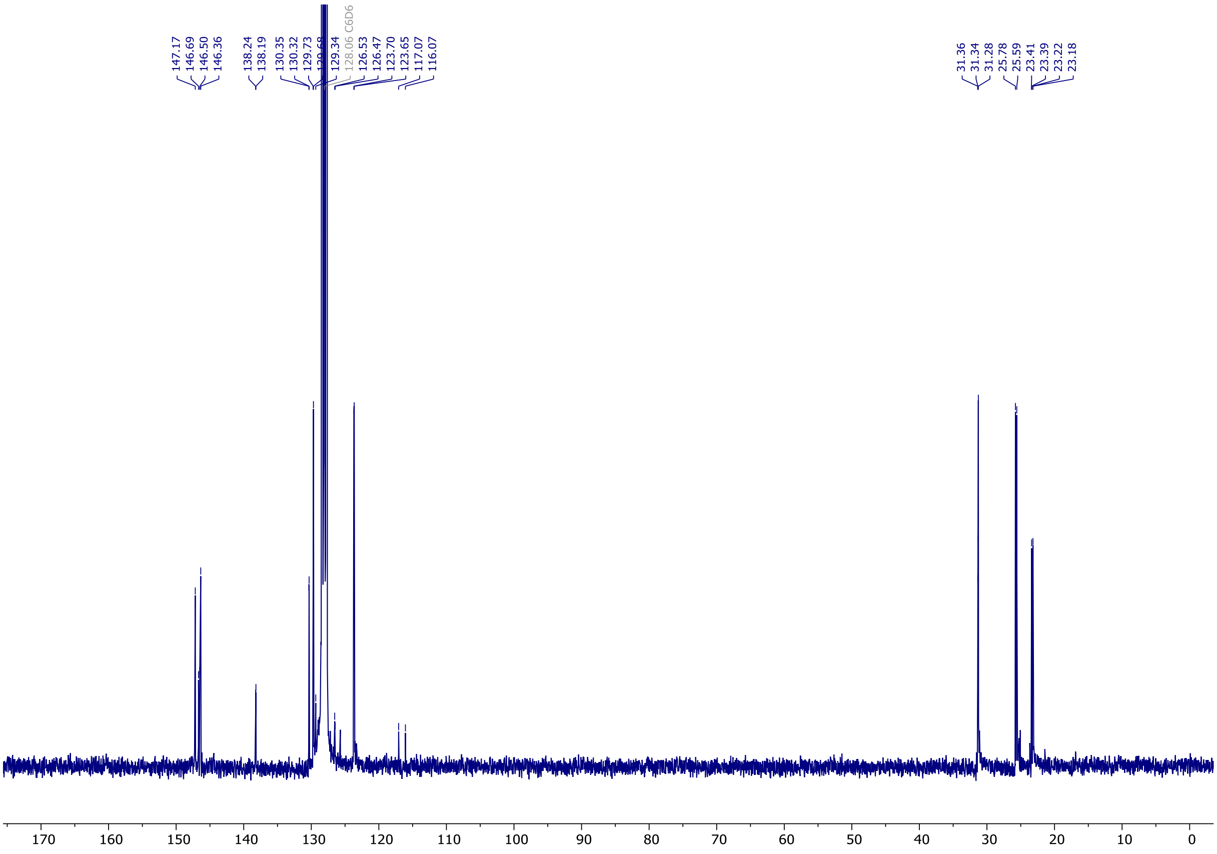

Figure S8: $^{31}P\{^{1}H\}$ NMR spectrum of ^{Dipp}**TerP(H)CN** (121.5 MHz, C_6D_6, rt).

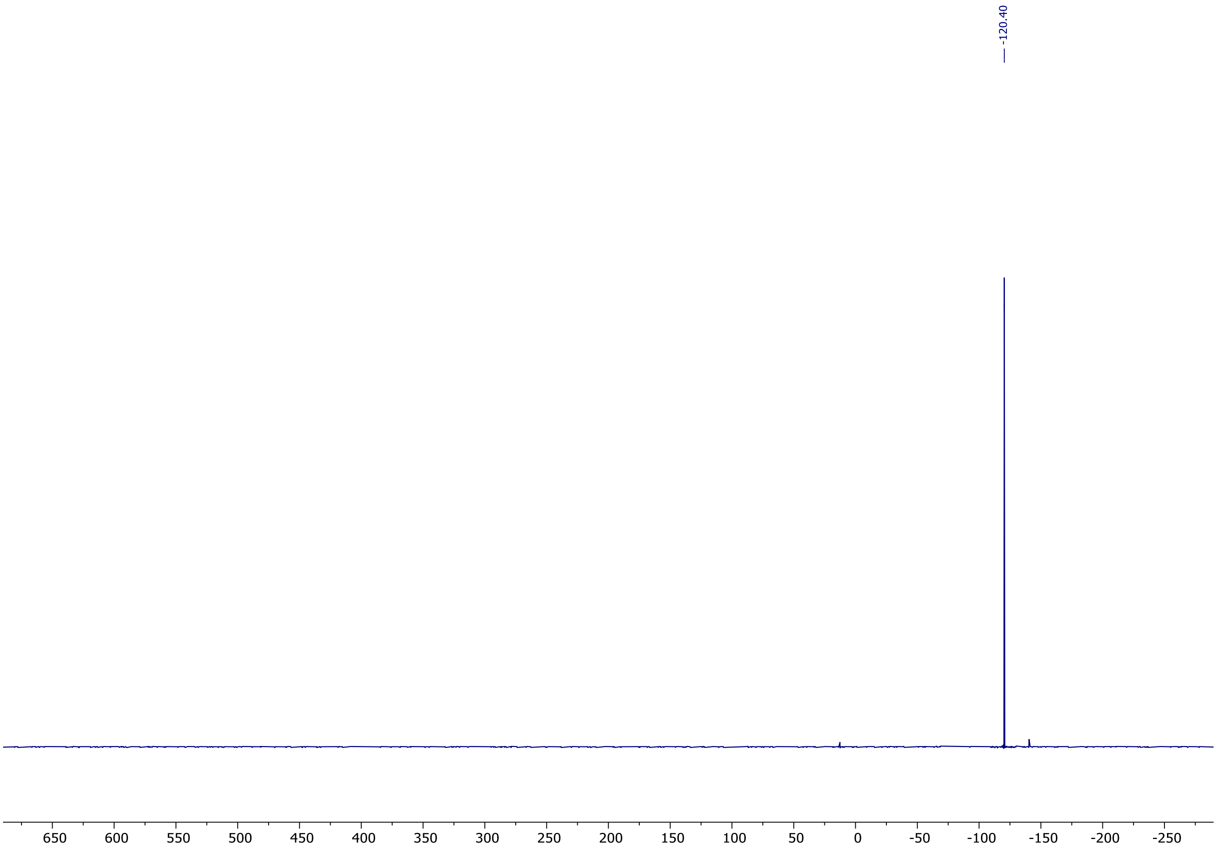

4 Syntheses of compounds

4.1 [(^{Dipp}TerPCN)K(18-crown-6)] (2)

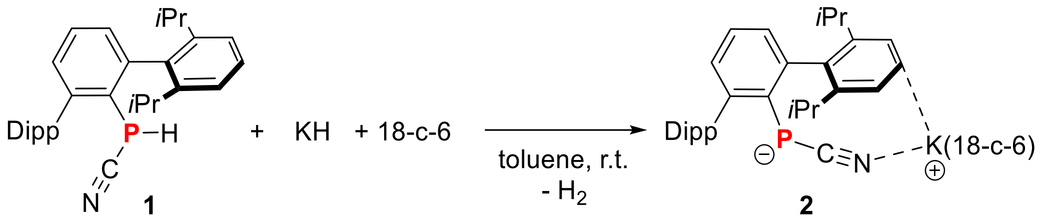

^{Dipp}TerP(H)CN (100 mg, 0.22 mmol), KH (10 mg, 0.25 mmol) and 18-crown-6 (58 mg, 0.22 mmol) were suspended in toluene (5 mL). The bright yellow suspension was stirred overnight at ambient temperature. Afterwards, all volatile components were removed *in vacuo* (1×10^{-3} mbar), the residue was re-dissolved in fresh toluene (15 mL) and insoluble solids were removed by cannula filtration. The clear filtrate was concentrated *in vacuo* (1×10^{-3} mbar) and stored at −30 °C, resulting in the deposition of bright yellow crystals. The supernatant was transferred to another flask and was used to get further fractions of crystalline product. The isolated crystals were dried *in vacuo* (1×10^{-3} mbar) for 2 hours at ambient temperature giving [(^{Dipp}TerPCN)K(18-crown-6)] (**2**) as a yellow crystalline solid. Yield: 115 mg (0.15 mmol, 69%).

CHN calc. (found) in %: C 68.13 (66.83), H 8.11 (6.45), N 1.85 (1.31); deviation probably due to incomplete combustion, repeated measurements with or without oxidizing agents did not result in better agreement. **^{31}P{^{1}H} NMR** (C_6D_6, 121.5 MHz): δ = −127.2 **^{1}H NMR** (C_6D_6, 300.2 MHz): δ = 1.35 (d, $^{3}J(^{1}H,^{1}H)$ = 6.7 Hz, 12 H, CH(C*H*$_3$)$_2$), 1.70 (d, $^{3}J(^{1}H,^{1}H)$ = 6.7 Hz, 12 H, CH(C*H*$_3$)$_2$), 3.00 (s, 24 H, 18-c-6), 3.49 (sept, $^{3}J(^{1}H,^{1}H)$ = 6.7 Hz, 4 H, C*H*(CH$_3$)$_2$), 7.00-7.07 (m, 3 H, *m*-C*H*$_{DipTer}$, *p*-C*H*$_{DipTer}$), 7.29-7.38 (m, 6 H, *m*-C*H*$_{Dip}$, *p*-C*H*$_{Dip}$). **^{13}C{^{1}H} NMR** (C_6D_6, 75.5 MHz): δ = 24.8 (d, $J_{P,C}$ = 2.4 Hz, CH(CH$_3$)$_2$), 25.6 (s, CH(CH$_3$)$_2$), 31.1 (s, *C*H(CH$_3$)$_2$), 69.9 (s, 18-c-6), 118.9 (s, CH$_{Aryl}$), 122.5 (s, CH$_{Aryl}$), 127.2 (s, CH$_{Aryl}$), 128.7 (d, $J_{P,C}$ = 2.0 Hz, CH$_{Aryl}$), 139.8 (d, $J_{P,C}$ = 19.2 Hz, C$_q$), 143.4 (d, $J_{P,C}$ = 5.1 Hz, C$_{q,Aryl}$), 148.3 (d, $J_{P,C}$ = 1.4 Hz, C$_q$) 152.9 (d, $J_{P,C}$ = 60.1 Hz, C$_q$P), PCN not detected. **IR**

(ATR, 32 scans, cm^{-1}): $\tilde{\nu}$ = 2953 (w), 2898 (w), 2861 (w), 2045 (w), 1573 (w), 1494 (w), 1455 (w), 1371 (w), 1351 (m), 1329 (w), 1304 (w), 1283 (w), 1249 (w), 1196 (w), 1175 (w), 1102 (s), 1057 (w), 1037 (w), 1003 (w), 960 (m), 872 (w), 837 (w), 803 (w), 781 (w), 758 (m), 735 (m), 697 (w), 633 (w), 606 (w), 585 (w), 555 (w), 428 (w), 529 (w), 512 (w), 467 (w). **MS** (ESI-TOF, pos., m/z, rel. int. > 10%): expected: m/z = 303.1205 [(18-crown-6)K]$^+$; found: m/z = 303.1209, (ESI-TOF, neg., m/z, rel. int. > 10%): expected: m/z = 454.2668 [^{Dipp}TerPCN]$^-$; found: m/z = 454.2677.

Single crystals of **2** suitable for X-ray diffraction can be grown from saturated toluene solution at −30 °C.

Figure S9: ^{1}H NMR spectrum of **2** (300.2 MHz, C_6D_6, rt).

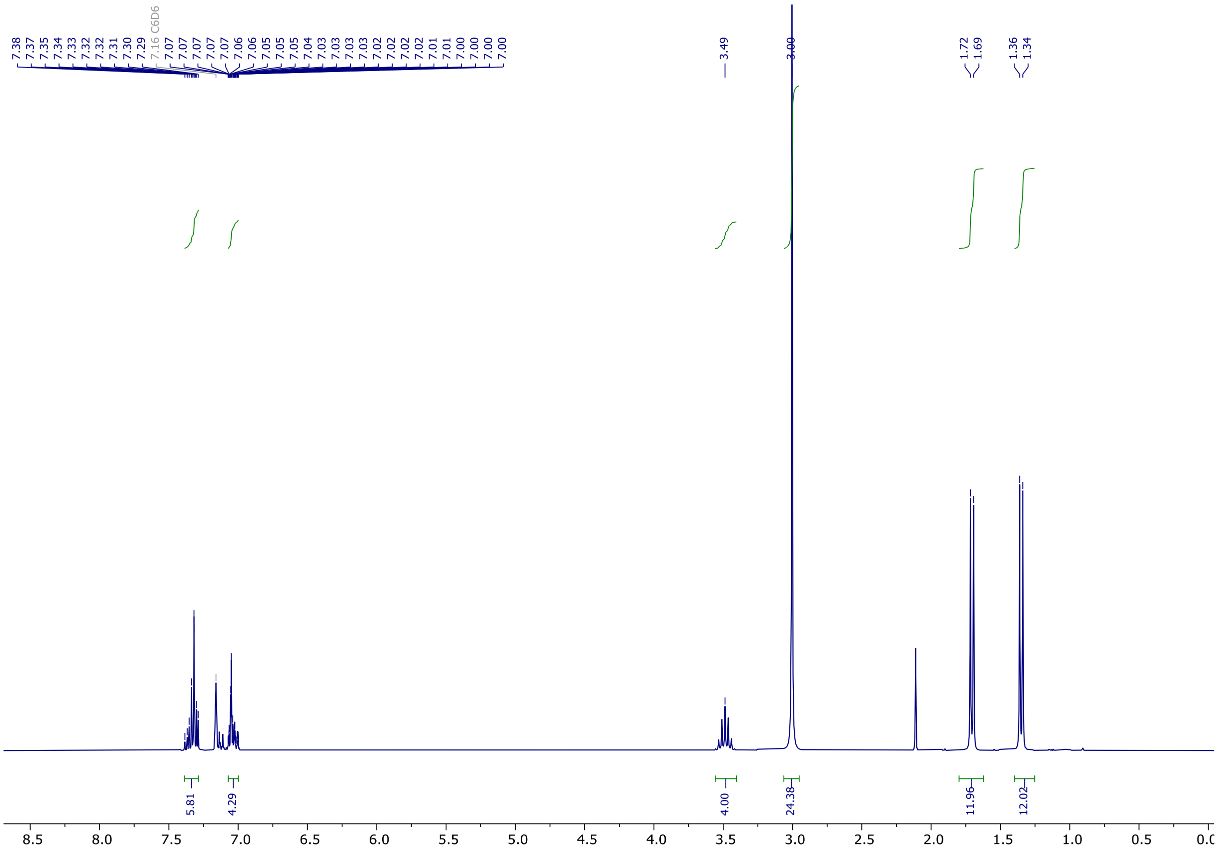

Figure S10: $^{13}C\{^1H\}$ NMR spectrum of **2** (75.5 MHz, C_6D_6, rt).

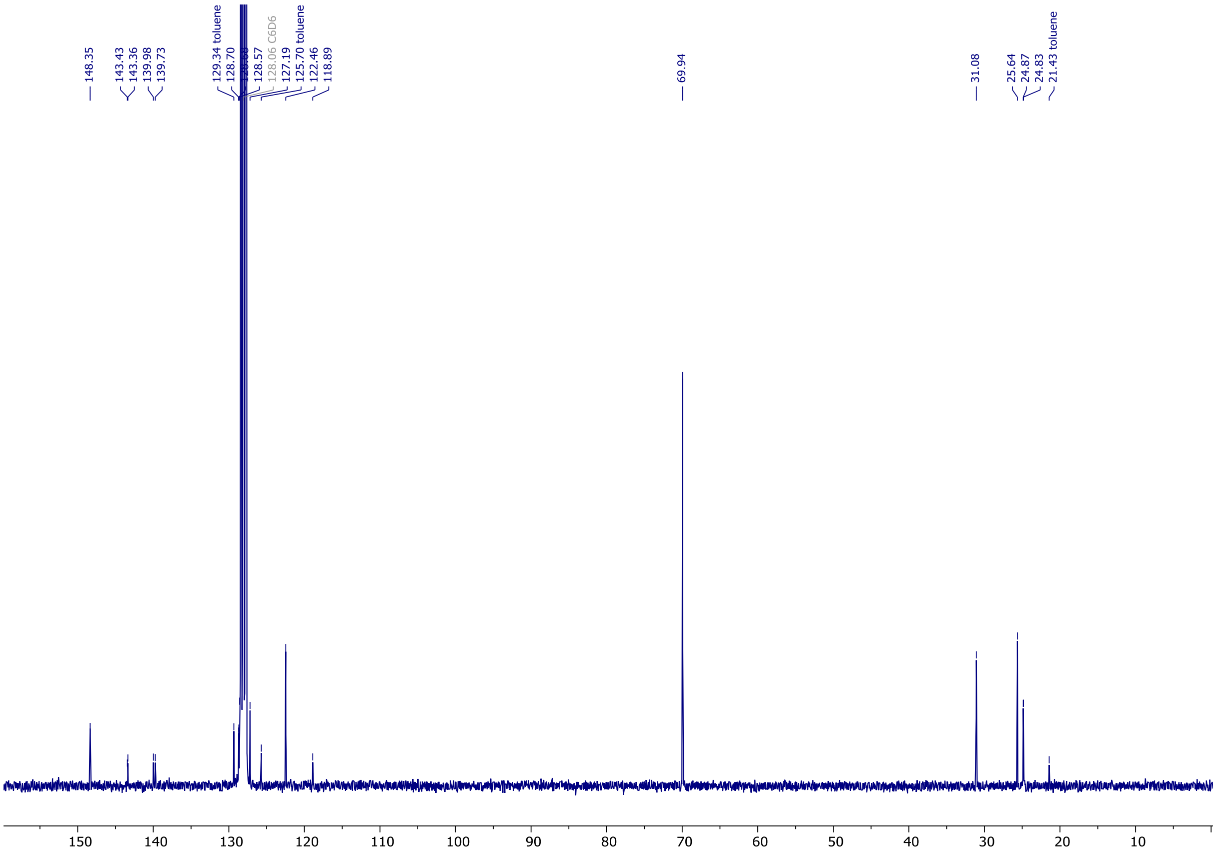

Figure S11: $^{31}P\{^1H\}$ NMR spectrum of **2** (121.5 MHz, C_6D_6, rt).

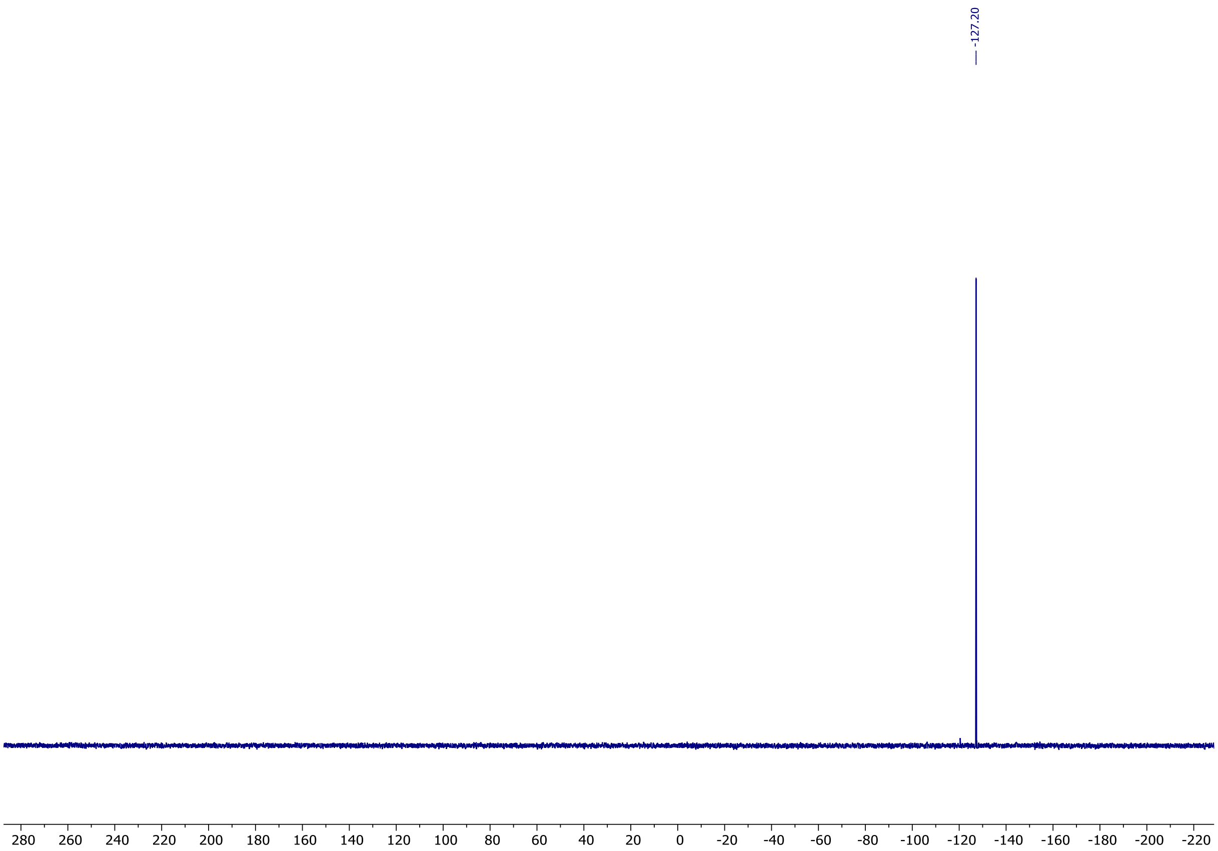

Figure S12: IR spectrum of **[(^{Dipp}TerPCN)K(18-crown-6)]** (ATR, solid sample, 32 scans, cm^{-1}).

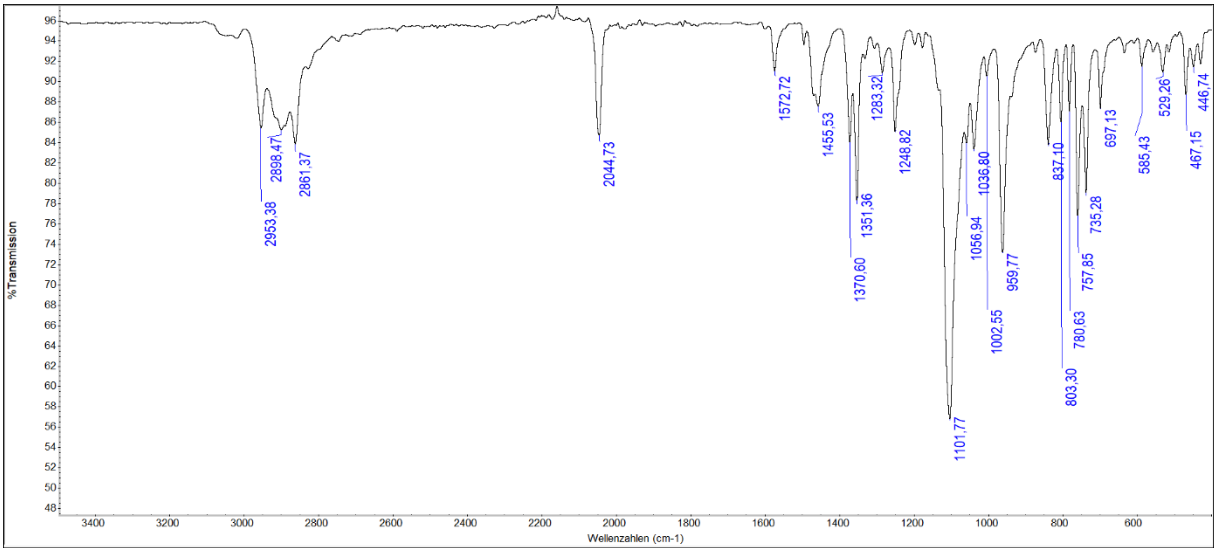

4.2 [(^{Dipp}TerPCN)Na(18-crown-6)] (3)

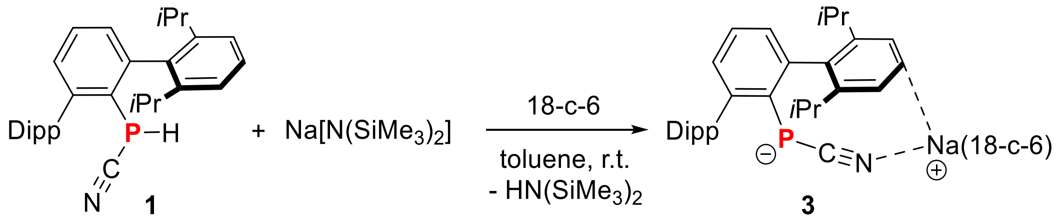

^{Dipp}TerP(H)CN (61 mg, 0.13 mmol), NaHMDS (27 mg, 0.15 mmol) and 18-crown-6 (38 mg, 0.14 mmol) were dissolved in toluene (5 mL). The orange suspension was stirred overnight at ambient temperature. Afterwards all volatile components were removed *in vacuo* (1×10^{-3} mbar) and the residue was re-dissolved in fresh toluene (5 mL). The clear orange solution was concentrated *in vacuo* (1×10^{-3} mbar) and stored at −30 °C for 2 days but no crystal was formed. Then the Schlenk flask was stored at −78 °C resulting in the formation of some seed crystals. Further crystallizations were done at −30 °C resulting in the deposition of bright yellow crystals. The supernatant was transferred to another flask and was used to get further fractions of crystalline product. The isolated crystals were dried *in vacuo* (1×10^{-3} mbar) for 2 hours at ambient temperature, yielding [(^{Dipp}TerPCN)Na(18-crown-6)] (**3**) as a yellow crystalline solid. Yield: 76 mg (0.1 mmol, 76%).

CHN calc. (found) in %: C 69.61 (68.01), H 8.29 (7.19), N 1.89 (1.43), were measured with oxidizing agent, vanadium pentoxide (V_2O_5). Deviations most likely result from incomplete combustion. **^{31}P{^{1}H} NMR** (C_6D_6, 121.5 MHz): δ = −129.3. **^{1}H NMR** (C_6D_6, 300.2 MHz): δ = 1.35 (d, $^3J(^1H,^1H)$ = 6.9 Hz, 12 H, CH(CH_3)$_2$), 1.69 (d, $^3J(^1H,^1H)$ = 6.9 Hz, 12 H, CH(CH_3)$_2$), 3.06 (s, 24 H, 18-c-6), 3.37 (sept, $^3J(^1H,^1H)$ = 6.7 Hz, 4 H, C*H*(CH$_3$)$_2$), 7.04 (m, 3 H, *m*-CH_{DipTer}, *p*-CH_{DipTer}), 7.27 (s, 6 H, *m*-CH_{Dip}, *p*-CH_{Dip}). **^{13}C{^{1}H} NMR** (C_6D_6, 75.5 MHz): δ = 24.8 (d, $J_{P,C}$ = 2.3 Hz, CH(CH$_3$)$_2$), 25.6 (s, CH(CH$_3$)$_2$), 31.0 (s, CH(CH$_3$)$_2$), 69.3 (s, 18-c-6), 119.1 (s, CH_{Aryl}), 122.5 (s, CH_{Aryl}), 127.3 (s, $C_{q,Aryl}$), 128.7 (d, $J_{P,C}$ = 2.0 Hz, CH_{Aryl}), 140.2 (d, $J_{P,C}$ = 20.6 Hz, $C_{q,Aryl}$), 143.3 (d, $J_{P,C}$ = 5.0 Hz, $C_{q,Aryl}$), 148.2 (d, $J_{P,C}$ = 1.5 Hz, $C_{q,Aryl}$), 152.6 (d, $J_{P,C}$ = 59.5 Hz, C_qP). **IR** (ATR, 32 scans, cm^{-1}): $\tilde{\nu}$ = 2952 (w), 2899 (w), 2860 (w), 2049 (m), 1574 (w), 1457 (w), 1371 (w), 1354 (w), 1328 (w), 1297 (w), 1248 (m),

1126 (m), 1095 (s), 1036 (m), 1002 (w), 983 (w), 944 (m), 933 (m), 861 (w), 835 (w), 823 (w), 806 (w), 780 (w), 758 (m), 731 (m), 694 (w), 641 (w), 602 (w), 587 (w), 553 (w), 538 (w), 508 (w), 468 (w), 430 (w). **MS** (ESI-TOF, pos., m/z, rel. int. > 10%): expected: m/z = 287.1466 [(18-crown-6)Na]$^+$; found: m/z = 287.1464, (ESI-TOF, neg., m/z, rel. int. > 10%): expected: m/z = 454.2668 [^{Dip}TerPCN]$^-$; found: m/z = 454.2665.

Single crystals of **3** suitable for X-ray diffraction can be grown from saturated toluene solution at −30 °C.

Figure S13: ^{1}H NMR spectrum of **3** (300.2 MHz, C_6D_6, rt).

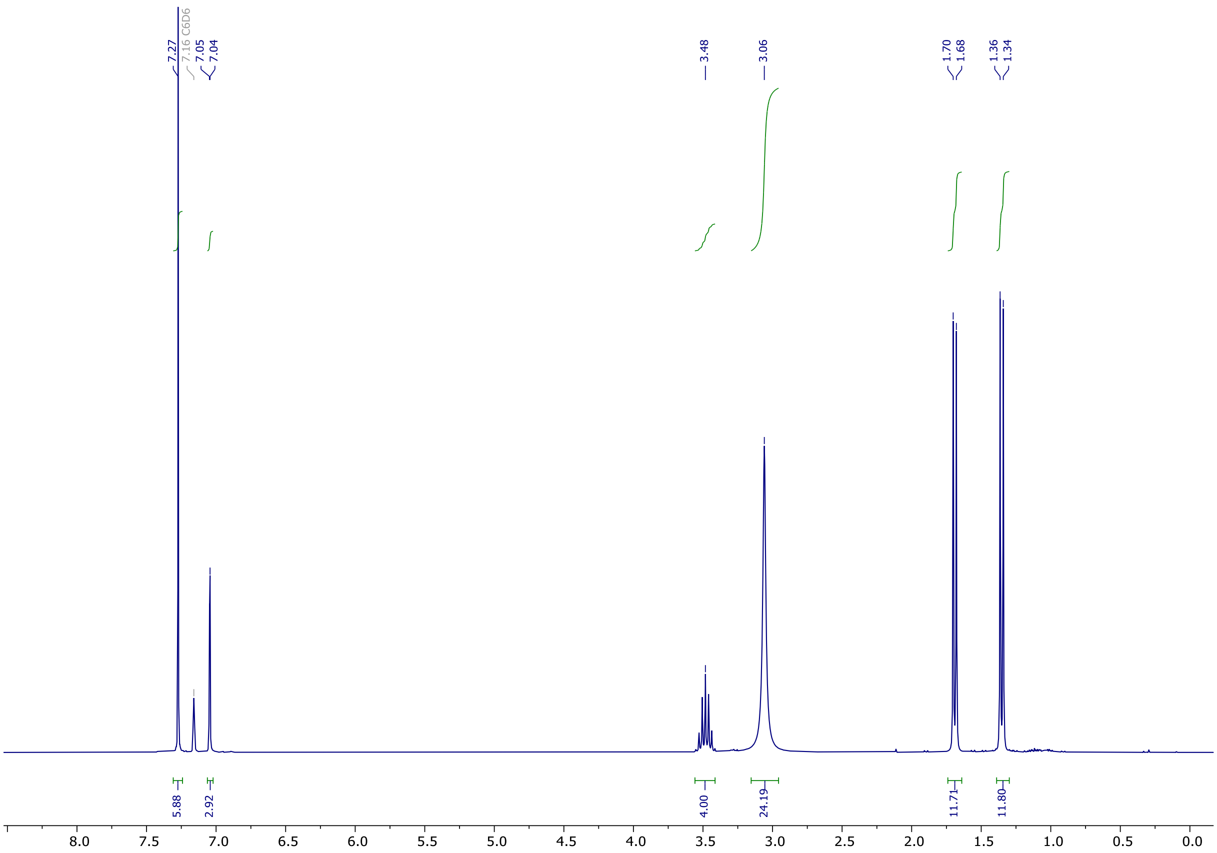

Figure S14: $^{13}C\{^1H\}$ NMR spectrum of **3** (75.5 MHz, C_6D_6, rt).

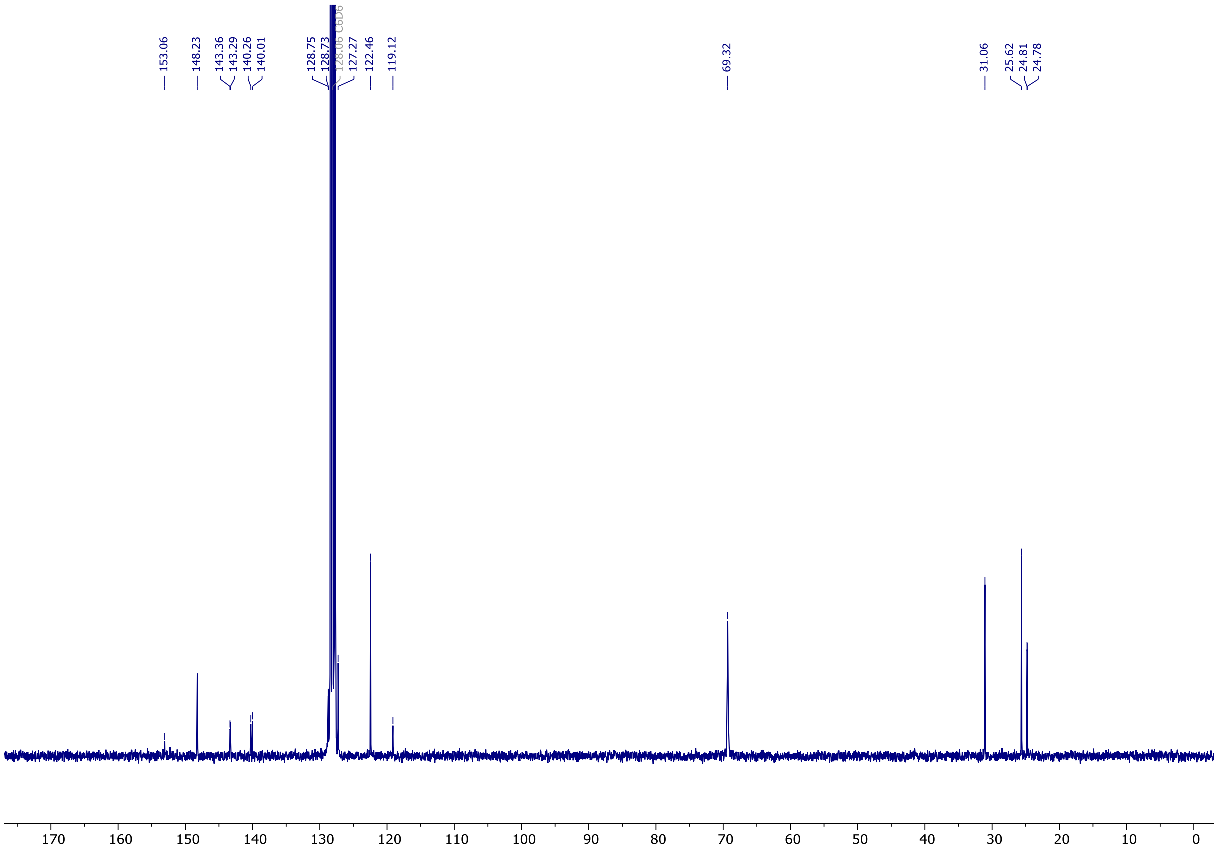

Figure S15: $^{31}P\{^1H\}$ NMR spectrum of **3** (121.5 MHz, C_6D_6, rt).

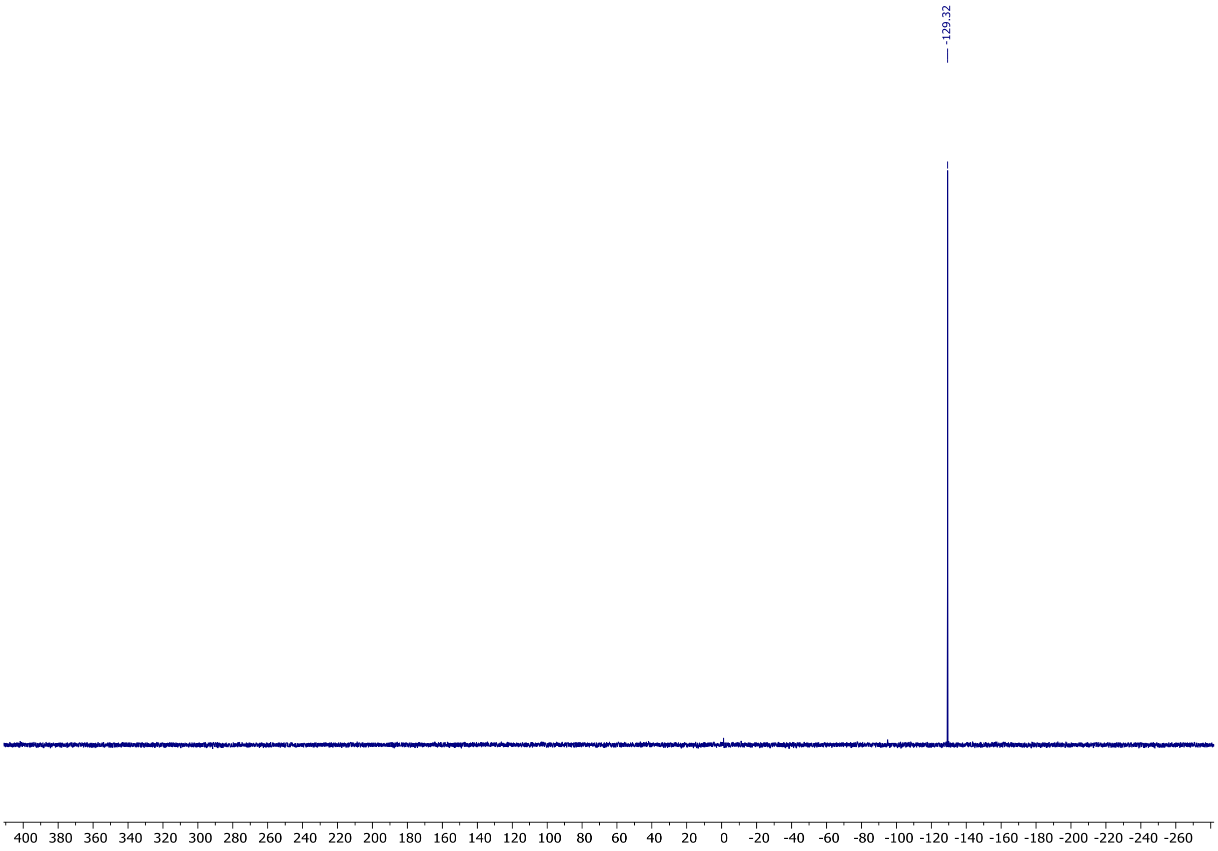

Figure S16: IR spectrum of **[(^{Dipp}TerPCN)Na(18-crown-6)]** (ATR, solid sample, 32 scans, cm^{-1}).

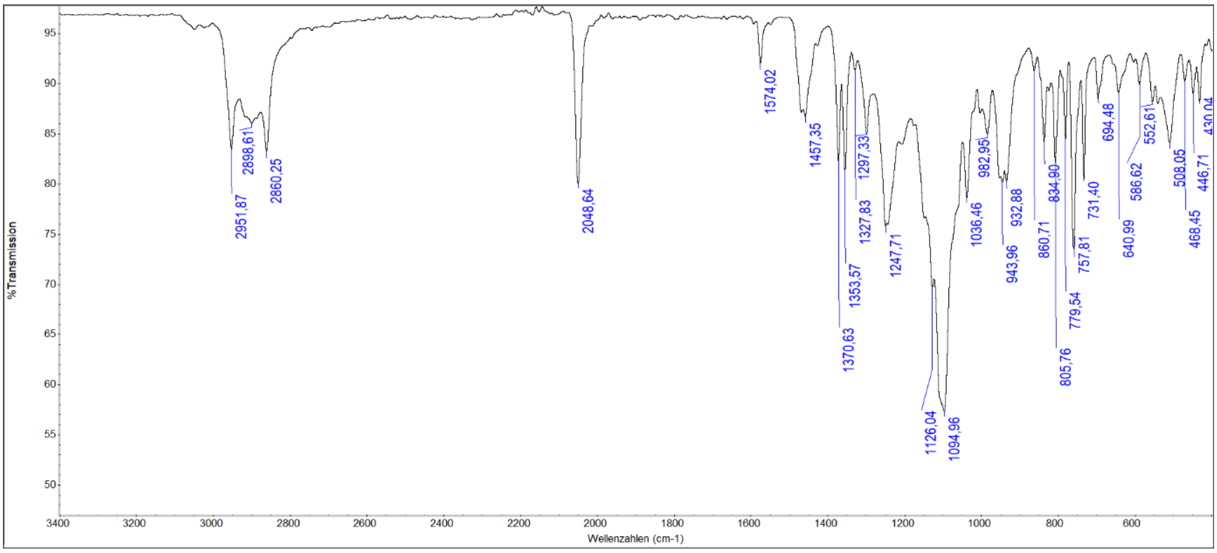

Figure S17: Cyclic voltammogram of [^{Dipp}TerPCN][Na(18-c-6)] in THF displays an irreversible oxidation process at –0.60 V (vs. Fc/Fc^{+}) followed by a minor anodic signal at around +0.44 V. Upon scanning cathodically a reduction process was registered at a peak potential of –2.62 V (vs. Fc/Fc^{+}). Upon scanning anodically solution changed from yellow to purple. Therefore, it was concluded that oxidation process at the peak potential of –0.60 V indeed corresponds to single-electron oxidation of [^{Dipp}TerPCN][Na(18-c-6)]. Upon scanning cathodically a reduction process was registered at peak potential of –2.62 V (vs. FcH/FcH+).

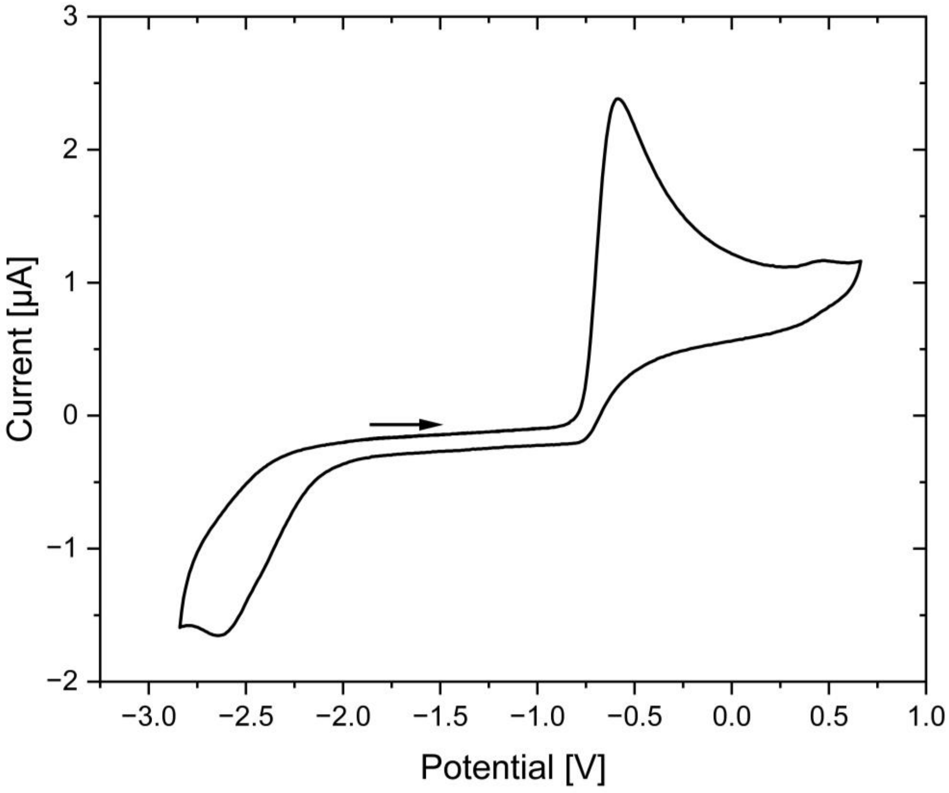

4.3 [(^{Dipp}TerPCN)Na(15-crown-5)] (4)

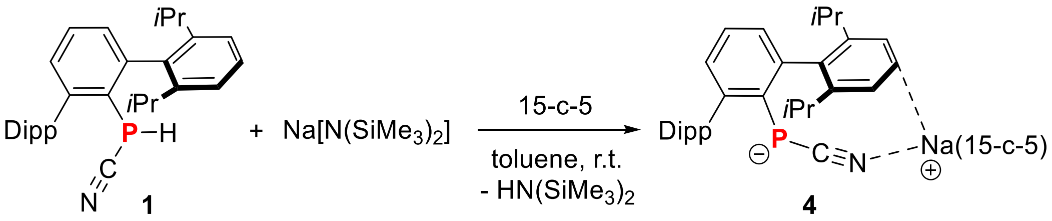

15-crown-5 (22 mg, 0.1 mmol), ^{Dipp}TerP(H)CN (41 mg, 0.09 mmol) and NaHMDS (18 mg, 0.1 mmol) were dissolved in toluene (2 mL). The resulting yellow suspension was stirred overnight at room temperature. Then all volatile components were removed *in vacuo* (1×10^{-3} mbar) and the residue was re-dissolved in fresh toluene (5 mL) and insoluble solids were removed by cannula filtration. The clear yellow solution was concentrated *in vacuo* (1×10^{-3} mbar) and stored at −30 °C, yielding yellow block-shaped crystals. The supernatant was transferred to another flask and was used to get further fractions of crystalline product. The isolated crystals were dried *in vacuo* (1×10^{-3} mbar) for 2 hours in a water bath (50 °C), yielding [(^{Dipp}TerPCN)Na(15-crown-5)] (**4**) as a yellow crystalline solid. Yield: 34 mg (0.05 mmol, 55%).

CHN calc. (found) in %: C 70.56 (69.10), H 8.23 (7.56), N 2.01 (1.69) were measured with oxidizing agent, vanadium pentoxide (V_2O_5). Deviations most likely result from incomplete combustion. **^{31}P{^{1}H} NMR** (C_6D_6, 121.5 MHz): δ = −129.9. **^{1}H NMR** (C_6D_6, 300.2 MHz): δ = 1.35 (d, $^3J(^1H,^1H)$ = 6.9 Hz, 12 H, CH(C*H*$_3$)$_2$), 1.70 (d, $^3J(^1H,^1H)$ = 6.9 Hz, 12 H, CH(C*H*$_3$)$_2$), 2.95 (s, 20 H, 15-c-5), 3.49 (sept, $^3J(^1H,^1H)$ = 6.9 Hz, 4 H, C*H*(CH$_3$)$_2$), 7.02-7.06 (m, 3 H, *m*-C*H*$_{DipTer}$, *p*-C*H*$_{DipTer}$), 7.17-7.30 (m, 6 H, *m*-C*H*$_{Dip}$, *p*-C*H*$_{Dip}$). **^{13}C{^{1}H} NMR** (C_6D_6, 75.5 MHz): δ = 24.8 (d, $J_{P,C}$ = 2.2 Hz, CH(CH$_3$)$_2$), 25.6 (s, CH(*C*H$_3$)$_2$), 31.1 (s, *C*H(CH$_3$)$_2$), 68.7 (s, 15-c-5), 119.1 (s, *C*H$_{Aryl}$), 122.4 (s, *C*H$_{Aryl}$), 126.7 (s, *C*$_{q,Aryl}$), 128.3 (d, $J_{P,C}$ = 1.9 Hz, *C*H$_{Aryl}$), 128.9 (s, *C*H$_{Aryl}$), 139.5 (s, *C*H$_{Aryl}$), 139.7 (d, $J_{P,C}$ = 18.9 Hz, *C*$_{q,Aryl}$), 143.0 (d, $J_{P,C}$ = 5.1 Hz, *C*$_{q,Aryl}$), 148.2 (d, $J_{P,C}$ = 1.4 Hz, *C*$_{q,Aryl}$). **IR** (ATR, 32 scans, cm^{-1}): $\tilde{\nu}$ = 3048 (w), 2954 (m), 2919 (w), 2864 (m), 2059 (m), 1573 (w), 1457 (w), 1370 (m), 1353 (m), 1329 (w), 1290 (w), 1247 (w), 1175 (w), 1113 (s), 1096 (s), 1056 (m), 1037 (m), 1003 (w), 943 (m), 861 (w), 829 (w), 803 (w), 779 (w), 757 (s), 731 (m), 697 (w), 689 (w), 585

(w), 554 (w), 540 (w), 522 (w), 465 (w), 452 (w). **MS** (ESI-TOF, pos., m/z, rel. int. > 10%): expected: m/z = 243.1203 [(15-crown-5)Na]$^+$; found: m/z = 243.1206, (ESI-TOF, neg., m/z, rel. int. > 10%): expected: m/z = 454.2668 [^{Dip}TerPCN]$^-$; found: m/z = 454.2669.

Single crystals of **4** suitable for X-ray diffraction can be grown from saturated toluene solution at −30 °C.

Figure S18: ^{1}H NMR spectrum of **4** (300.2 MHz, C_6D_6, rt).

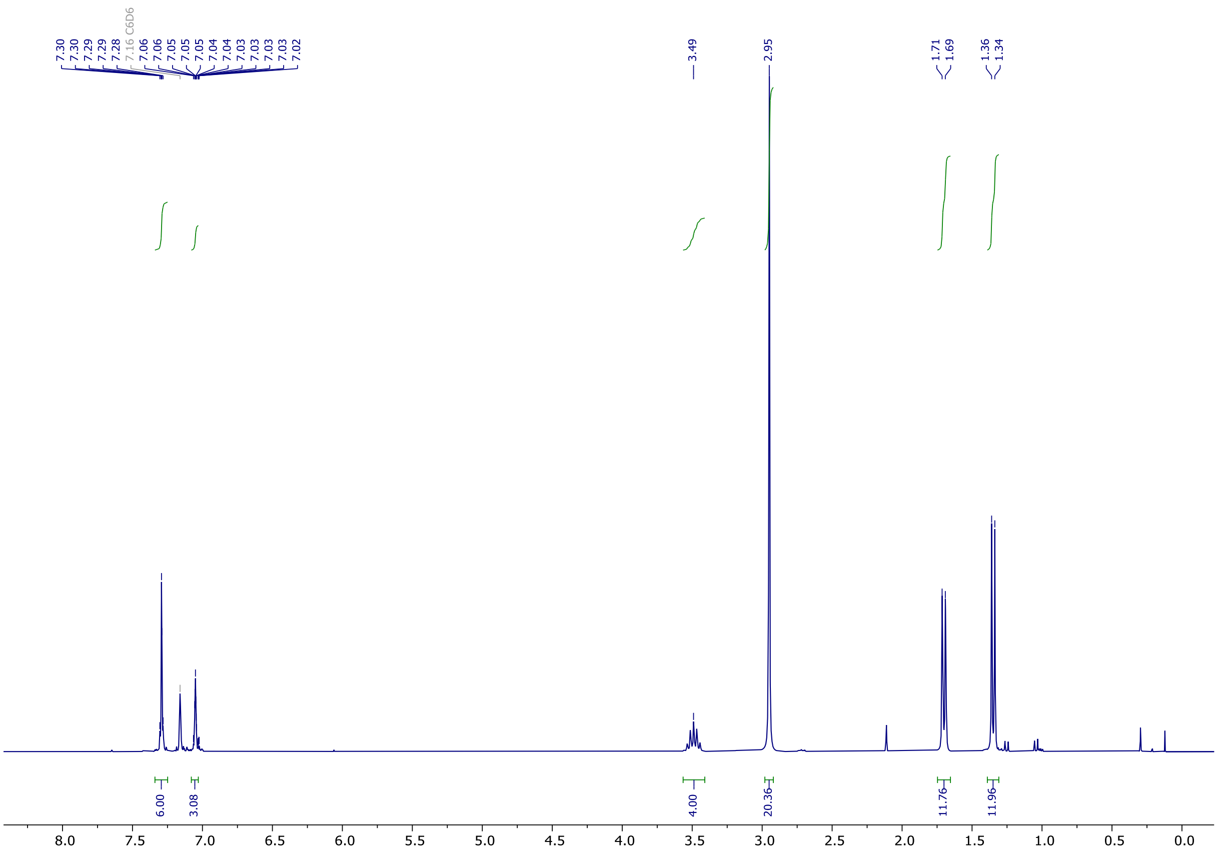

Figure S19: $^{13}C\{^1H\}$ NMR spectrum of **4** (75.5 MHz, C_6D_6, rt).

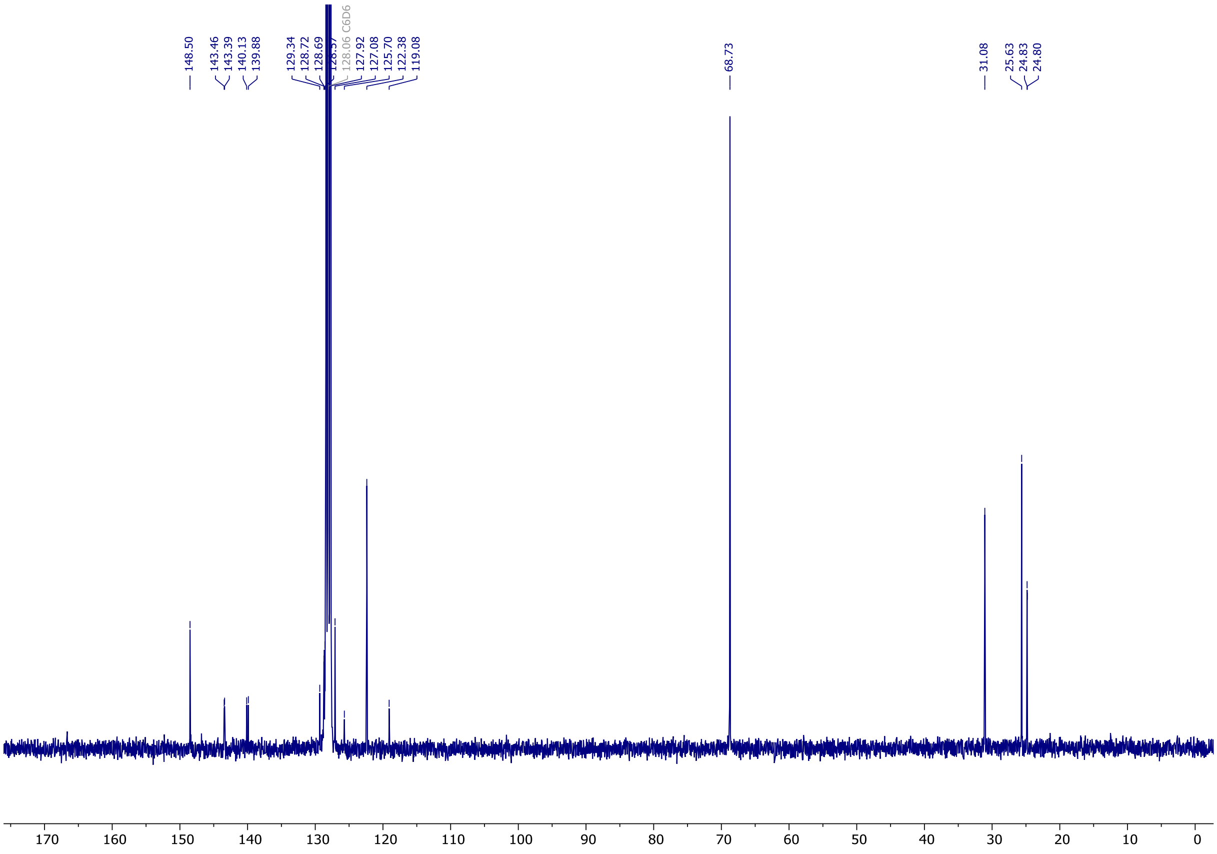

Figure S20: $^{31}P\{^1H\}$ NMR spectrum of **4** (121.5 MHz, C_6D_6, rt).

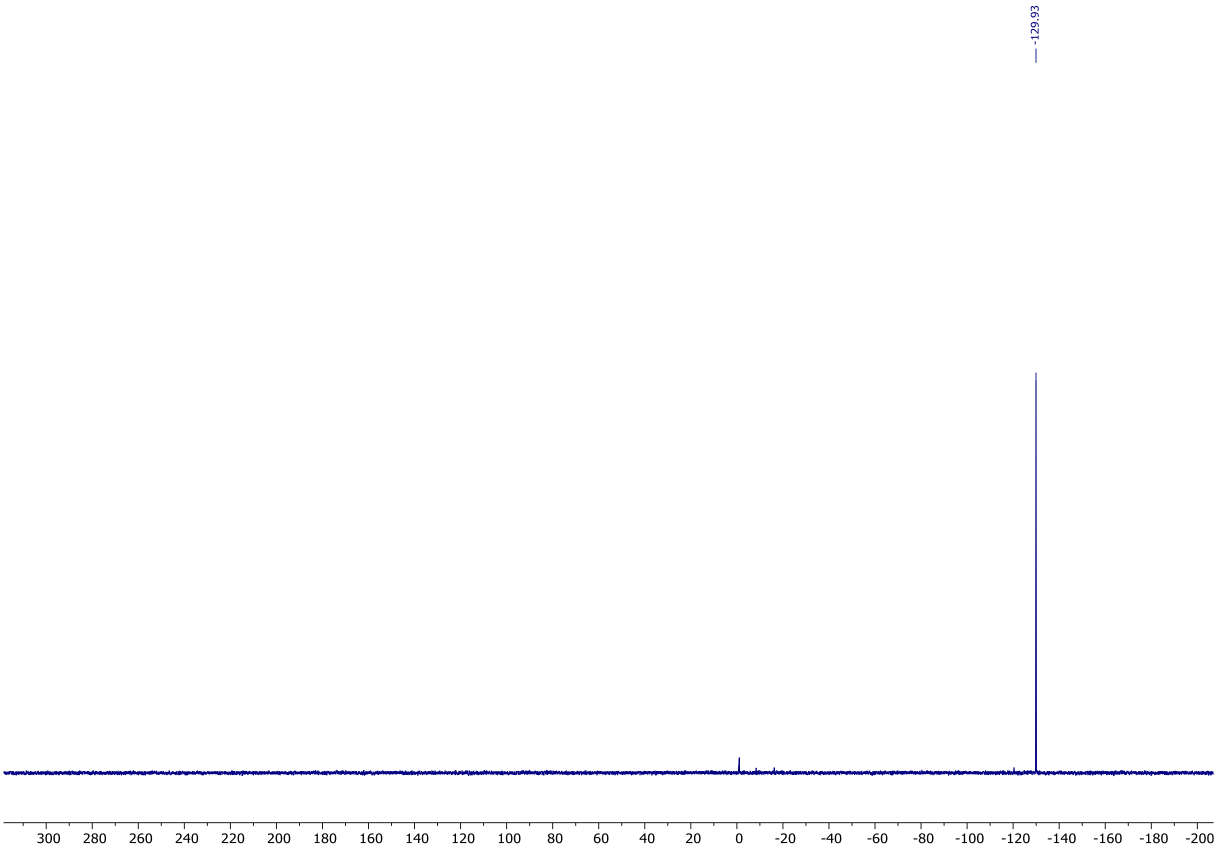

Figure S21: IR spectrum of **[(^{Dipp}TerPCN)Na(15-crown-5)]** (ATR, solid sample, 32 scans, cm^{-1}).

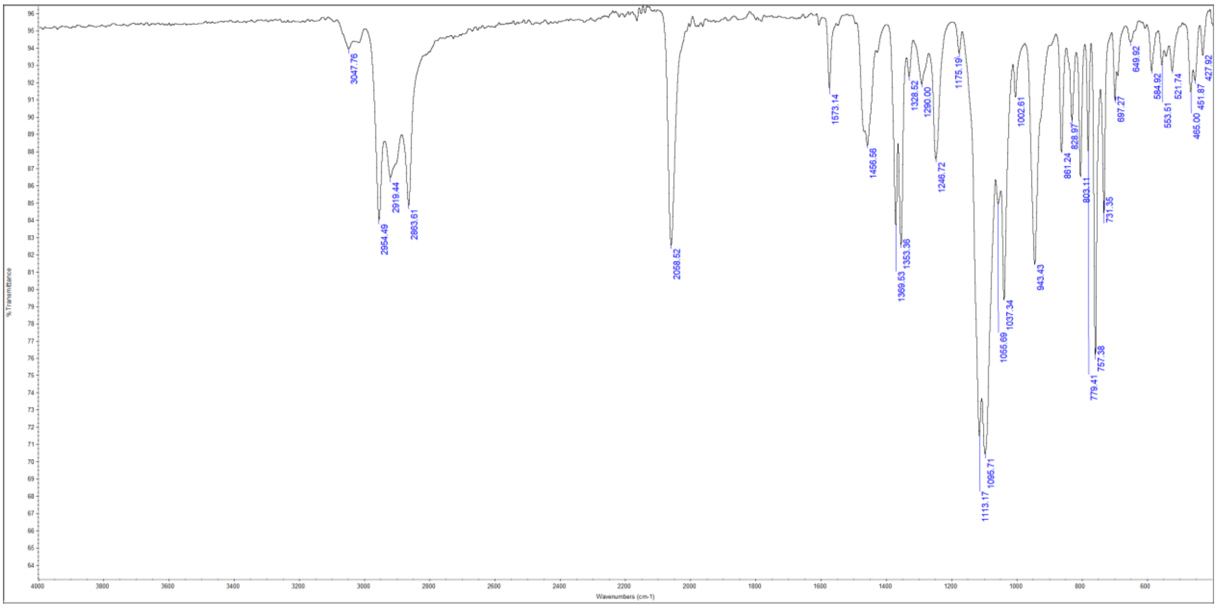

4.4 [^{Dipp}TerPO$_2$(CN)][K(18-crown-6)] (2·O$_2$)

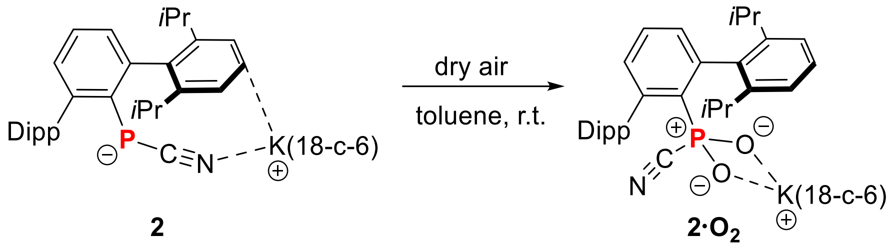

A) In a 10 mL Schlenk flask [^{Dipp}TerPCN][K(18-c-6)] salt (40 mg, 0.05 mmol) was dissolved in toluene (2 mL) and the resulting solution was degassed via freeze-pump-thaw for 3 times. The evacuated flask was filled with 1 atm of dried air (passed through Sicapent®) and the reaction mixture was stirred for 15 minutes at ambient temperature. The vessel was opened to a flow of dry air at 1 atm. As the reaction progresses, the initially bright yellow suspension turns into a brown then deep purple solution. The deep purple colour stays for 2 minutes and then faded to a light pink solution. Further stirring for 25 minutes shows that the light pink solution remains unchanged. Then all volatile components were removed *in vacuo* (1×10^{-3} mbar). The residue was redissolved in toluene (5 mL) and the clear filtrate was concentrated *in vacuo* (1×10^{-3} mbar) and stored at −30 °C, resulting in the deposition of colourless crystals. The supernatant was transferred to another flask and was used to get further fractions of crystalline product. The yield was rather poor and alternative approach was sought.

B) [^{Dipp}TerPO$_2$(CN)][K(18-c-6)] can also be synthesized directly from ^{Dipp}TerP(H)CN. In a 10 mL Schlenk flask ^{Dipp}TerP(H)CN (40 mg, 0.09 mmol), KH (4 mg, 0.15 mmol), and 18-crown-6 (24 mg, 0.09 mmol) were suspended in toluene (2 mL). The reaction mixture was stirred overnight for the in situ generation of [^{Dipp}TerPCN][K(18-c-6)] and afterwards the reaction mixture was degassed via three freeze-pump-thaw cycles. The evacuated flask was filled with 1 atm of dried air and the reaction mixture was stirred for 40 minutes at ambient temperature. The colour change observed was the same as in route **A**. The work-up after the reaction is the same as for route **A**. The isolated crystals were dried *in vacuo* (1×10^{-3} mbar) for 2 hours in a water bath (30 °C), yielding

^{Dipp}TerPO$_2$(CN)][K(18-crown-6)] (**2·O$_2$**) as a colourless crystalline solid. Yield: 42 mg (0.05 mmol, 60%).

CHN calc. (found) in %: C 65.37 (65.35), H 7.78 (7.39), N 1.77 (1.58) were measured with oxidizing agent, vanadium pentoxide (V_2O_5). **^{31}P{^{1}H} NMR** (C_6D_6, 121.5 MHz): δ = −9.0, −9.3. **^{1}H NMR** (C_6D_6, 400.1 MHz): δ = 1.21 (d, $^3J(^1H,^1H)$ = 6.9 Hz, 6 H, CH(CH_3)$_2$), 1.23 (d, $^3J(^1H,^1H)$ = 6.9 Hz, 6 H, CH(CH_3)$_2$), 1.60 (d, $^3J(^1H,^1H)$ = 6.9 Hz, 6 H, CH(CH_3)$_2$), 1.61 (d, $^3J(^1H,^1H)$ = 6.9 Hz, 6 H, CH(CH_3)$_2$), 3.16 (s, 24 H, 18-c-6), 3.20 (sept, $^3J(^1H,^1H)$ = 6.9 Hz, 4 H, CH(CH$_3$)$_2$), 7.21-7.22 (m, 3 H, *m*-CH_{DipTer}, *p*-CH_{DipTer}), 7.23-7.27 (m, 4 H, *m*-CH_{Dip}), 7.28-7.33 (m, 2 H, *p*-CH_{Dip}). **^{13}C{^{1}H} NMR** (C_6D_6, 101 MHz): δ = 23.8 (s, PCN, CH(CH_3)$_2$), 24.0 (s, PNC, CH(CH_3)$_2$), 25.80 (s, PNC, CH(CH_3)$_2$), 25.84 (s, PCN, CH(CH_3)$_2$), 31.46 (s, PNC, CH(CH$_3$)$_2$), 31.54 (s, PCN, CH(CH$_3$)$_2$), 69.9 (s, 18-c-6), 121.97 (s, PNC, CH_{Aryl}), 122.08 (s, PCN, CH_{Aryl}), 124.2 (d, $^1J_{P,C}$ = 90.7 Hz, P*C*≡N), 127.2 (s, CH_{Aryl}), 127.5 (s, CH_{Aryl}), 128.3 (s, CH_{Aryl})*, 131.2 (d, $J_{P,C}$ = 2.7 Hz, CH_{Aryl}), 131.4 (s, CH_{Aryl}), 141.7 (d, $J_{P,C}$ = 3.7 Hz, $C_{q,Aryl}$), 142.6 (d, $J_{P,C}$ = 3.2 Hz, $C_{q,Aryl}$), 144.4 (d, $J_{P,C}$ = 9.7 Hz, $C_{q,Aryl}$), 145.1 (d, $J_{P,C}$ = 9.8 Hz, $C_{q,Aryl}$), 147.1 (s, $C_{q,Aryl}$), 147.5 (s, $C_{q,Aryl}$) * overlap with C_6D_6 signal. **IR** (ATR, 32 scans, cm^{-1}): $\tilde{\nu}$ = 3048 (vw), 2958 (w), 2884 (w), 2865 (w), 2826 (w), 2084 (w), 1579 (vw), 1560 (vw), 1468 (w), 1430 (w), 1379 (vw), 1352 (m), 1286 (m), 1272 (m), 1253 (w), 1142 (m), 1109 (vs), 1059 (w), 963 (m), 841 (m), 824 (w), 804 (w), 794 (w), 750 (m), 711 (w), 614 (w), 585 (s), 569 (w), 563 (w), 536 (m), 499 (w), 458 (w). **Raman** (633 nm, 20 s, 10 scans, cm^{-1}): $\tilde{\nu}$ = 3064 (2), 3046 (5), 3040 (3), 2956 (7), 2927 (7), 2902 (9), 2866 (10), 2844 (6), 2808 (3), 2785 (1), 2752 (1), 2727 (1), 2701 (1), 2683 (1), 2175 (8), 2084 (3), 1594 (3), 1580 (2), 1564 (1), 1472 (5), 1459 (4), 1346 (1), 1333 (1), 1309 (2), 1277 (10), 1246 (2), 1229 (3), 1171 (1), 1160 (2), 1139 (3), 1108 (3), 1093 (4), 1047 (4), 1004 (5), 972 (1), 959 (2), 887 (6), 872 (6), 834 (1), 755 (1), 710 (1), 662 (1), 614 (9), 589 (1), 547 (1), 494 (2), 458 (1), 445 (1), 411 (1), 343 (3), 282 (8), 234 (3), 200 (1). **MS** (ESI-TOF, pos., m/z, rel. int. > 10%): expected: m/z = 303.1205 [(18-crown-6)K]$^+$; found: m/z = 303.1208, (ESI-TOF, neg., m/z, rel. int. > 10%): expected: m/z = 486.2567 [^{Dip}TerPO$_2$(CN)]$^-$; found: m/z = 486.2567.

Single crystals of **2·O₂** suitable for X-ray diffraction can be grown from saturated toluene solution at −30 °C.

Figure S22: ^{1}H NMR spectrum of **[^{Dipp}TerPO$_2$(CN)][K(18-crown-6)]** (400.1 MHz, C_6D_6, rt).

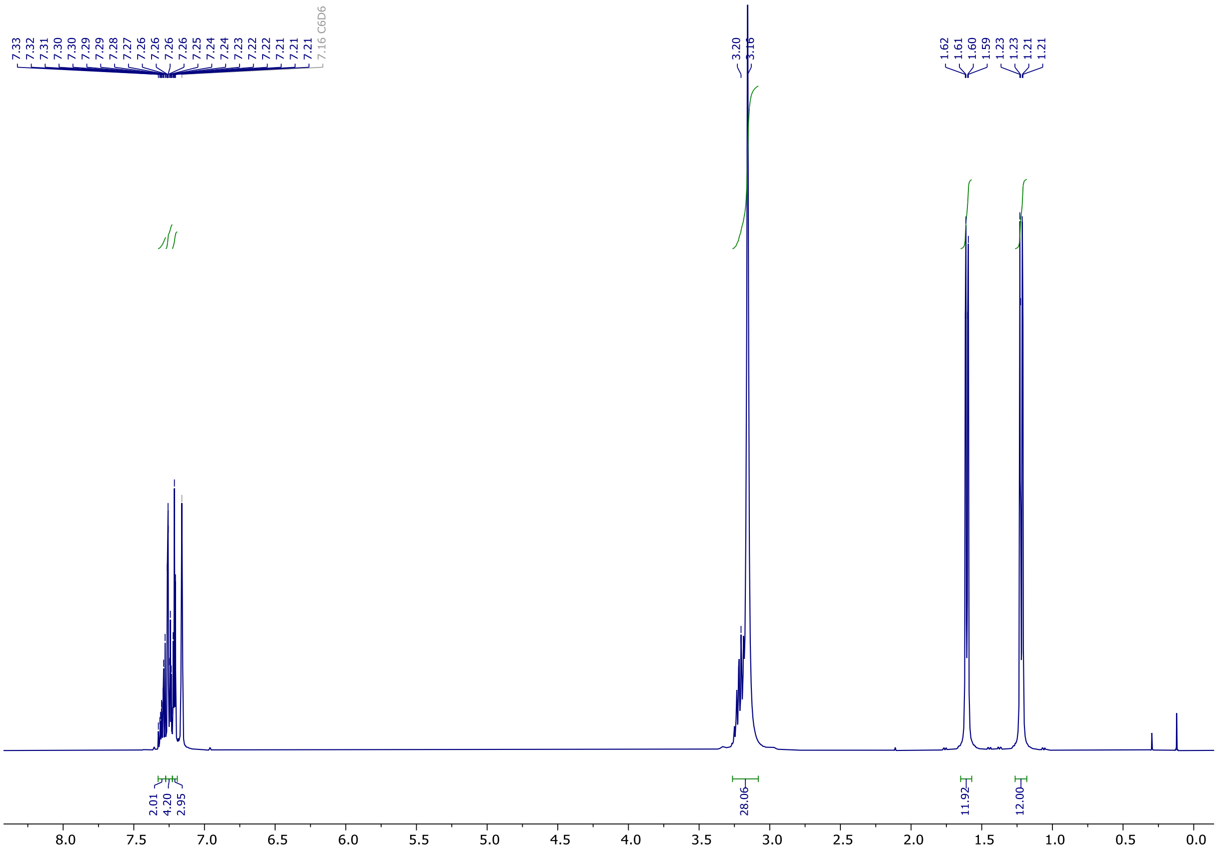

Figure S23: $^{13}C\{^1H\}$ NMR spectrum of **[^{Dipp}TerPO$_2$(CN)][K(18-crown-6)]** (101 MHz, C_6D_6, rt).

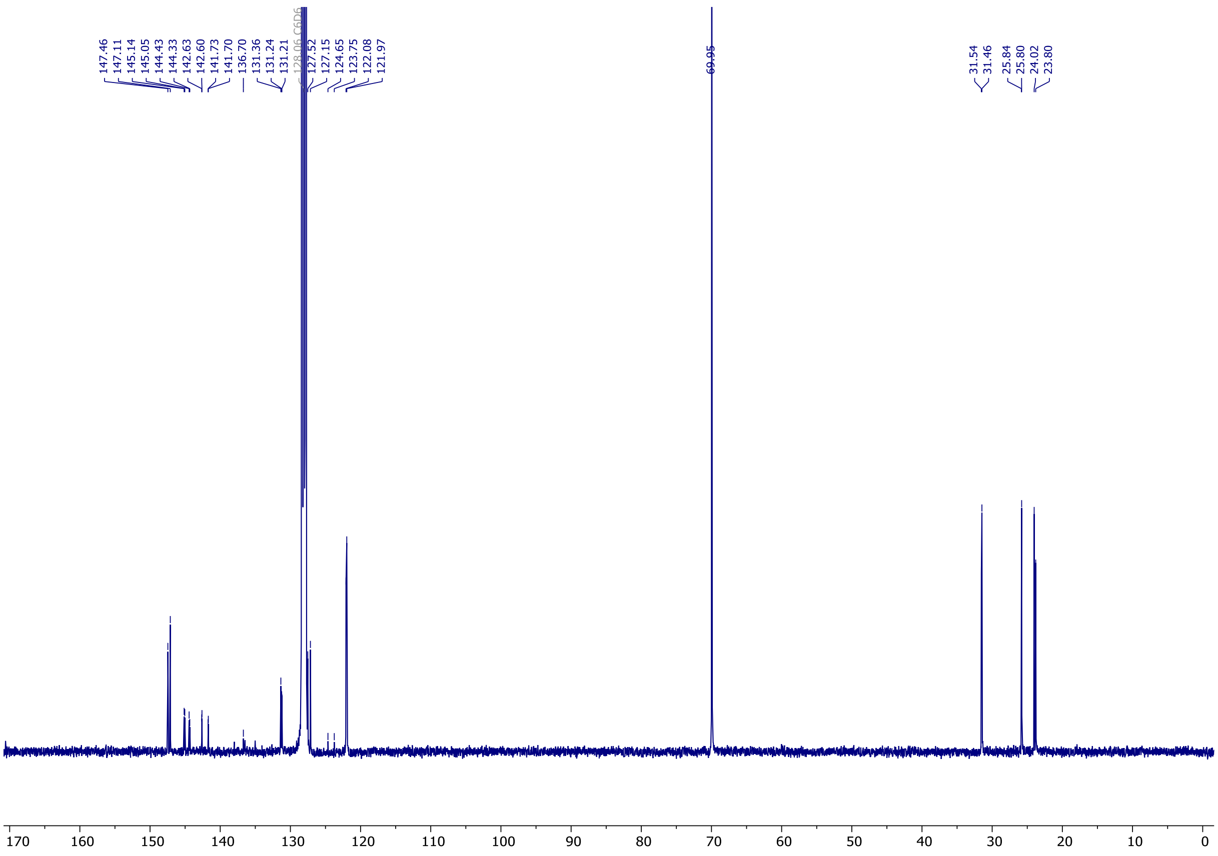

Figure S24: $^{31}P\{^1H\}$ NMR spectrum of **[^{Dipp}TerPO$_2$(CN)][K(18-crown-6)]** (121.5 MHz, C_6D_6, rt).

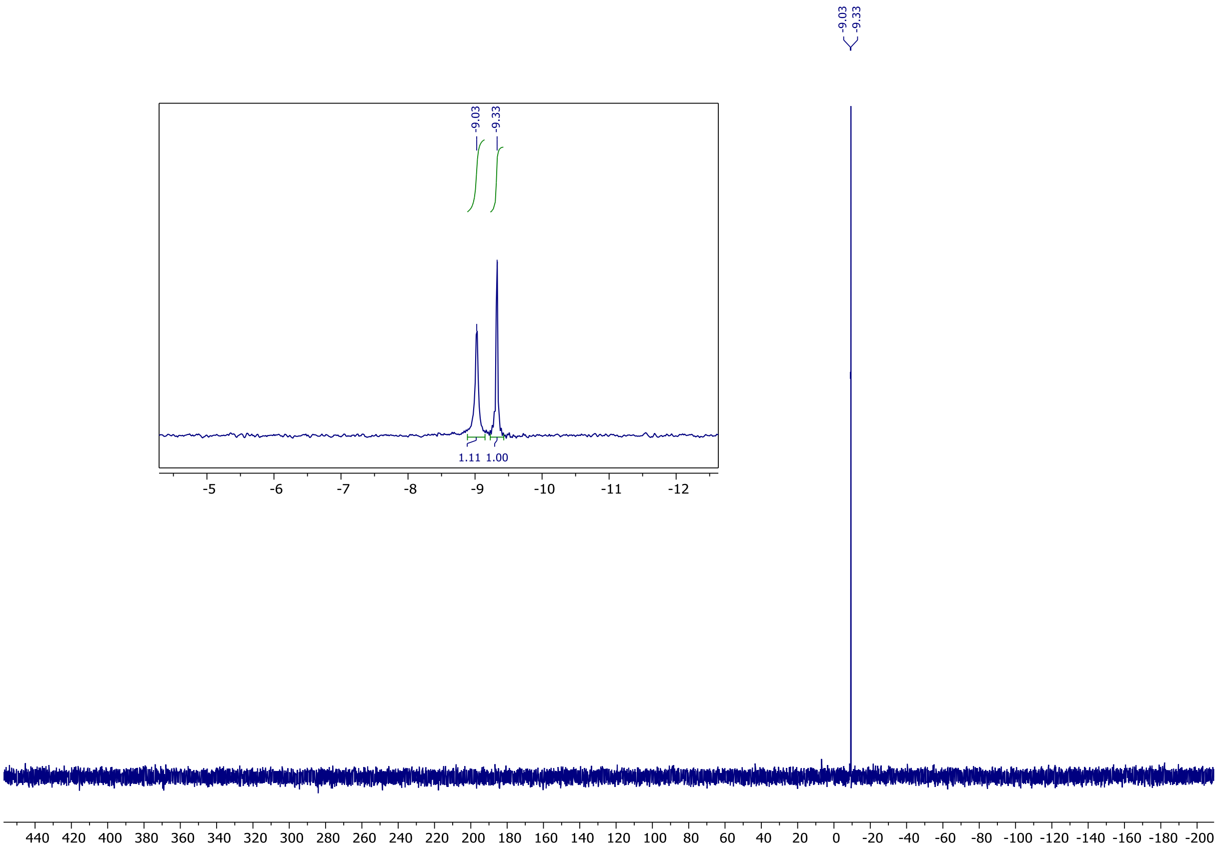

Figure S25: IR (ATR, solid sample, 32 scans, cm^{-1}) and Raman (633 nm, 20 s, 10 scans, cm^{-1}) spectra of isolated crystals of **[^{Dipp}TerPO$_2$(CN)][K(18-crown-6)]**.

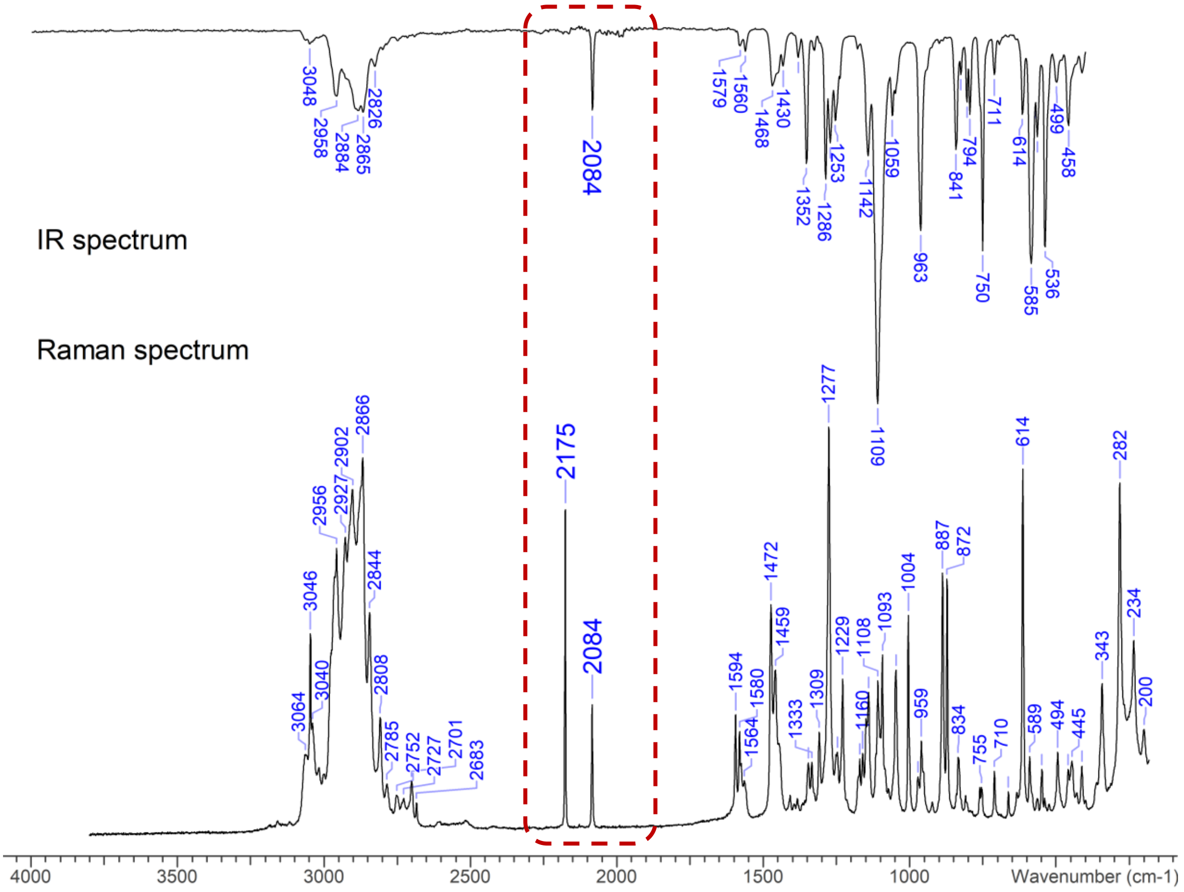

4.4.1 Heating of [^{Dipp}TerPO$_2$(CN)][K(18-crown-6)] in solution at 80 °C

In a Young NMR tube [^{Dip}TerPO$_2$(CN)][K(18-c-6)] (15 mg, 0.02 mmol) (as a mixture of PCN and PNC isomers) was dissolved in C_6D_6 (0.6 mL) and was heated in an oil bath at 80 °C overnight. Single crystals suitable for X-ray diffraction can be grown from a saturated toluene solution at −30 °C, yielding white block-shaped crystals of **2·O$_2$**. Yield: 8.5 mg (0.01 mmol, 57%). The NMR data was recorded after recrystallization showing that the tentative isocyanide isomer was completely transformed into the cyanide isomer (δ(^{31}P) = −9.4 ppm).

^{31}P{^{1}H} NMR (C_6D_6, 121.5 MHz): δ = −9.4. **^{1}H NMR** (C_6D_6, 300.2 MHz): δ = 1.21 (d, $^3J(^1H,^1H)$ = 6.9 Hz, 12 H, CH(C*H*$_3$)$_2$), 1.61 (d, $^3J(^1H,^1H)$ = 6.7 Hz, 12 H, CH(C*H*$_3$)$_2$), 3.16 (s, 24 H, 18-c-6), 3.21 (sept, $^3J(^1H,^1H)$ = 7.0 Hz, 4 H, C*H*(CH$_3$)$_2$), 7.21 (d, $^3J(^1H,^1H)$ = 3.0 Hz, 3 H, *m*-C*H*$_{DipTer}$, *p*-C*H*$_{DipTer}$), 7.23-7.26 (m, 4 H, *m*-C*H*$_{Dip}$), 7.29-7.34 (m, 2 H, *p*-C*H*$_{Dip}$). **IR** (ATR, 32 scans, cm^{-1}): $\tilde{\nu}$ = 3048 (vw), 2957 (w), 2864 (w), 2827 (w), 1578 (vw), 1560 (vw), 1467 (w), 1352 (m), 1271 (m), 1251 (m), 1140 (m), 1109 (vs), 1059 (w), 1047 (w), 962 (m), 841 (m), 824 (w), 804 (w), 793 (w), 750 (m), 709 (w), 614 (w), 587 (s), 563 (w), 538 (m), 495 (w), 460 (w), 429 (w), 412 (w). **Raman** (633 nm, 5 s, 5 scans, cm^{-1}): $\tilde{\nu}$ = 3046 (4), 3039 (2), 2906 (4), 2867 (4), 2843 (3), 2808 (1), 2752 (1), 2727 (1), 2701 (1), 2683 (1), 2175 (3), 1594 (4), 1580 (1), 1563 (1), 1473 (3), 1457 (2), 1345 (1), 1333 (2), 1276 (10), 1247 (1), 1229 (1), 1170 (2), 1160 (1), 1138 (3), 1093 (5), 1045 (4), 1003 (2), 958 (1), 951 (1), 886 (2), 871 (3), 834 (1), 755 (2), 709 (1), 661 (1), 632 (1), 625 (1), 613 (5), 589 (1), 562 (1), 549 (1), 546 (1), 538 (1), 493 (2), 485 (1), 458 (1), 445 (1), 411 (1), 399 (1), 360 (1), 342 (2), 325 (1), 313 (1), 300 (1), 282 (3), 266 (1), 256 (1), 234 (2), 214 (1), 199 (1).

Figure S26: ^{1}H NMR spectrum of the cyanide isomer of [^{Dipp}TerPO$_2$(CN)][K(18-crown-6)] (300.2 MHz, C_6D_6, rt).

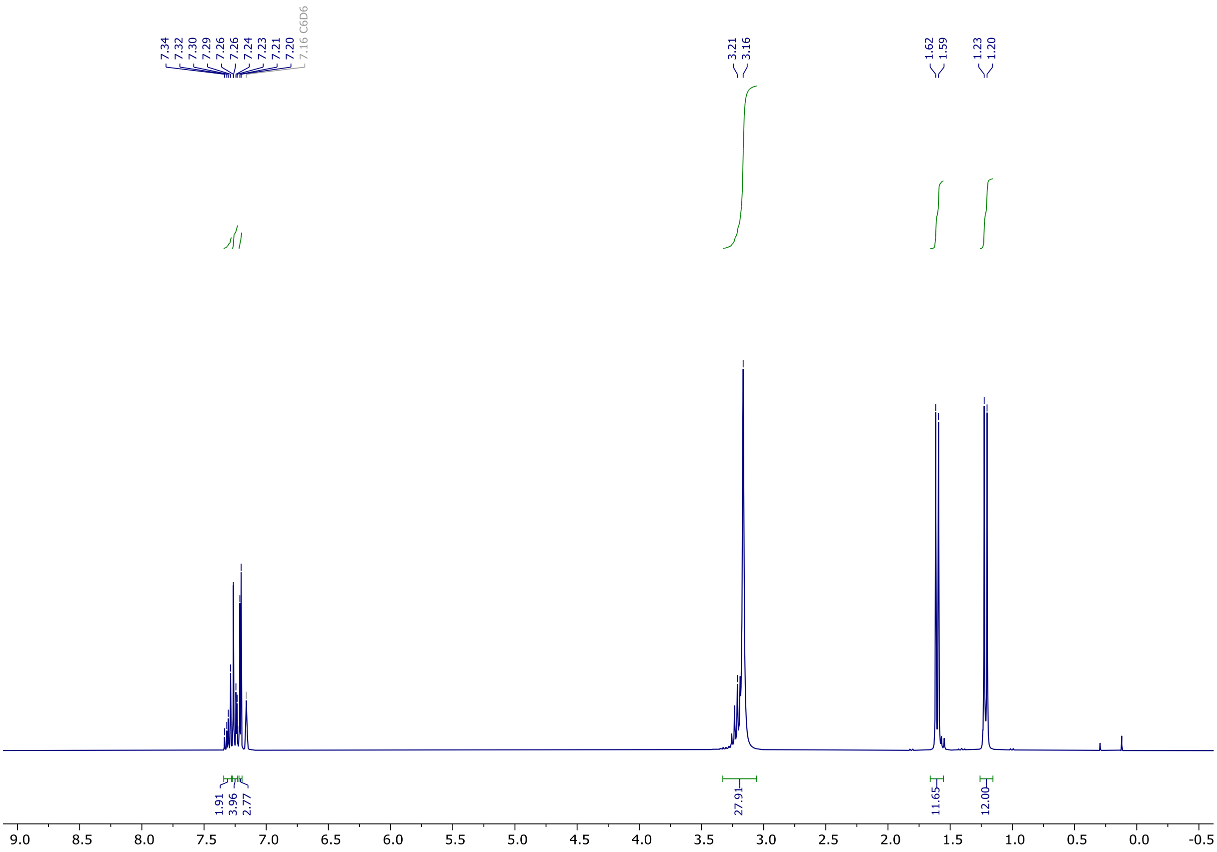

Figure S27: ^{31}P NMR spectrum of the cyanide isomer of [^{Dipp}TerPO$_2$(CN)][K(18-crown-6)] (121.5 MHz, C_6D_6, rt).

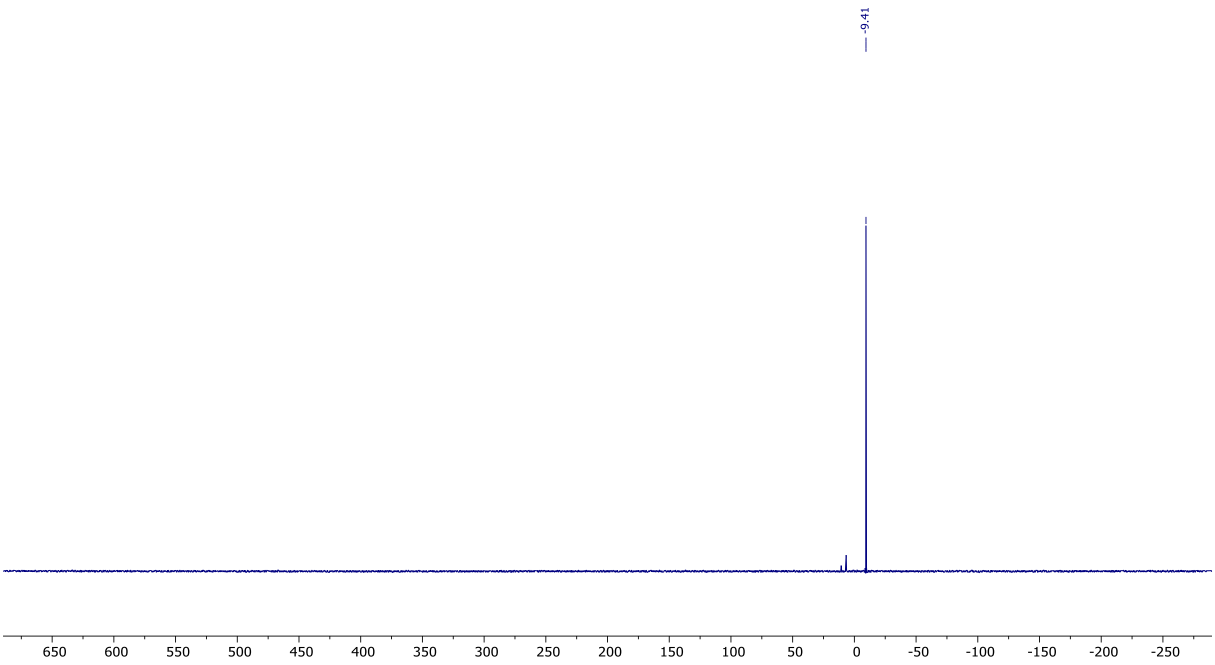

Figure S28: IR (ATR, solid sample, 32 scans, cm^{-1}) and Raman (633 nm, 5 s, 5 scans, cm^{-1}) spectra of isolated crystals of **[^{Dip}TerPO$_2$(CN)][K(18-crown-6)]** after heating a C_6D_6 solution of **2·O$_2$** to 80 °C.

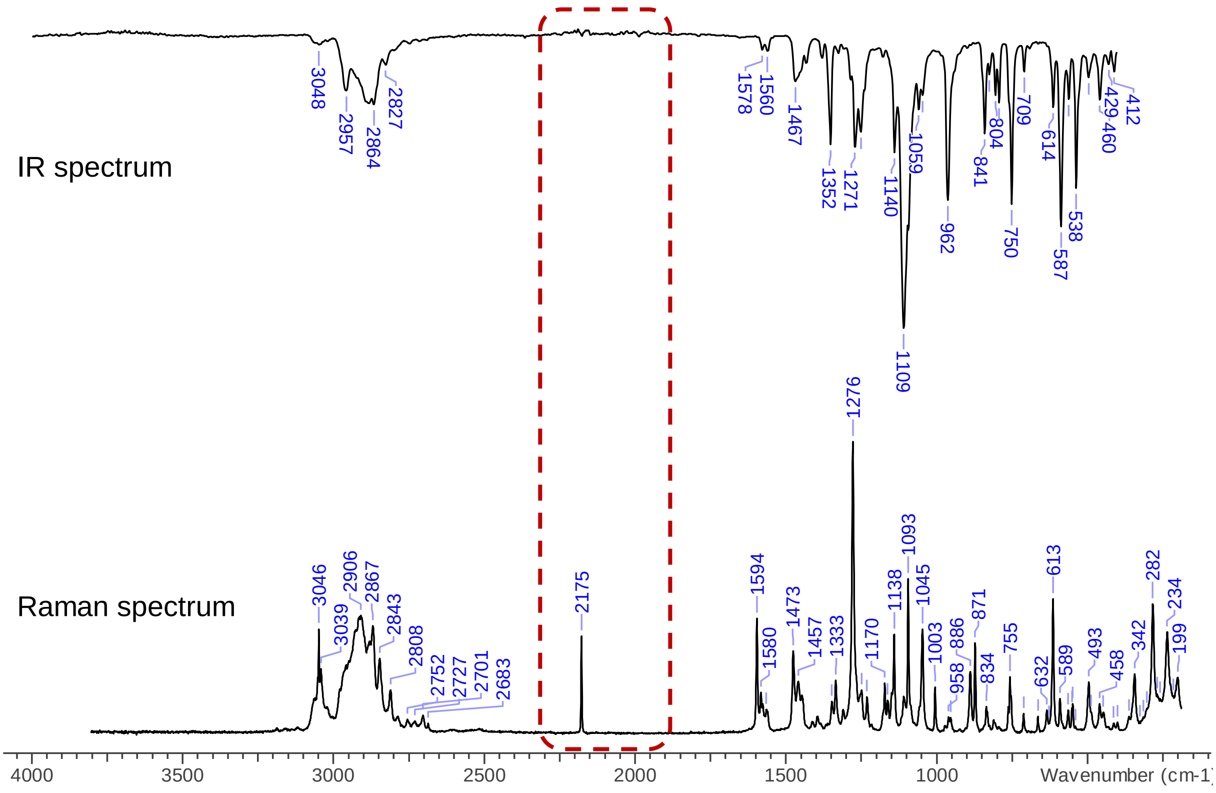

4.4.2 Heating of [^{Dipp}TerPO$_2$(CN)][K(18-crown-6)] in the solid state at 110 °C

In a Young NMR tube a sample of crystalline [^{Dipp}TerPO$_2$(CN)][K(18-c-6)] (as a mixture of PCN and PNC isomers) (10 mg, 0.02 mmol) was heated in an oil bath at 110 °C overnight (16 h). After heating the crystals were dissolved in C_6D_6 and NMR spectra were recorded, revealing that most of the PNC-isomer was converted to the PCN isomer. This is evident from the major ^{31}P NMR resonance at 9.3 ppm (PCN, vide supra, 4.4.1) with only a small peak at 9.0 ppm (PNC) in a 9:1 ratio. Moreover, only one set of signals for the ^{Dipp}Ter group was detected in both the ^{1}H and ^{13}C NMR spectra.

^{31}P{^{1}H} NMR (C_6D_6, 162.01 MHz): δ = −9.3 (*P*CN), −9.0 (*P*NC). **^{1}H NMR** (C_6D_6, 400.1 MHz): δ = 1.22 (d, $^3J_{H,H}$ = 6.9 Hz, 12 H, CH(C*H*$_3$)$_2$), 1.61 (d, ^3J = 6.7 Hz, 12 H, CH(C*H*$_3$)$_2$), 3.17 (s, 24 H, 18-c-6), 3.22 (sept, $^3J_{H,H}$ = 7.0 Hz, 4 H, C*H*(CH$_3$)$_2$), 7.18-7.21 (m, *m*-C*H*$_{\text{DipTer}}$, *p*-C*H*$_{\text{DipTer}}$), 7.23-7.27 (m, 4 H, *m*-C*H*$_{\text{Dip}}$), 7.29-7.34 (m, 2 H, *p*-C*H*$_{\text{Dip}}$). **^{13}C{^{1}H} NMR** (C_6D_6, 101 MHz): δ = 23.8 (s, CH(CH$_3$)$_2$), 25.84 (s, CH(CH$_3$)$_2$), 31.54 (s, CH(CH$_3$)$_2$), 69.9 (s, 18-c-

6), 122.1 (s, CH_{Aryl}), 124.2 (d, $^1J_{P,C}$ = 90.7 Hz, PC≡N), 127.2 (s, CH_{Aryl}), 131.2 (d, $J_{P,C}$ = 2.7 Hz, CH_{Aryl}), 141.7 (d, $J_{P,C}$ = 3.5 Hz, $C_{q,Aryl}$), 144.4 (d, $J_{P,C}$ = 9.8 Hz, $C_{q,Aryl}$), 147.5 (s, $C_{q,Aryl}$).

Figure S29: ^{1}H NMR spectrum of the cyanide isomer of [^{Dipp}TerPO$_2$(CN)][K(18-crown-6)] after heating a isomer mixture in the solid state at 110 °C (400.1 MHz, C_6D_6, rt).

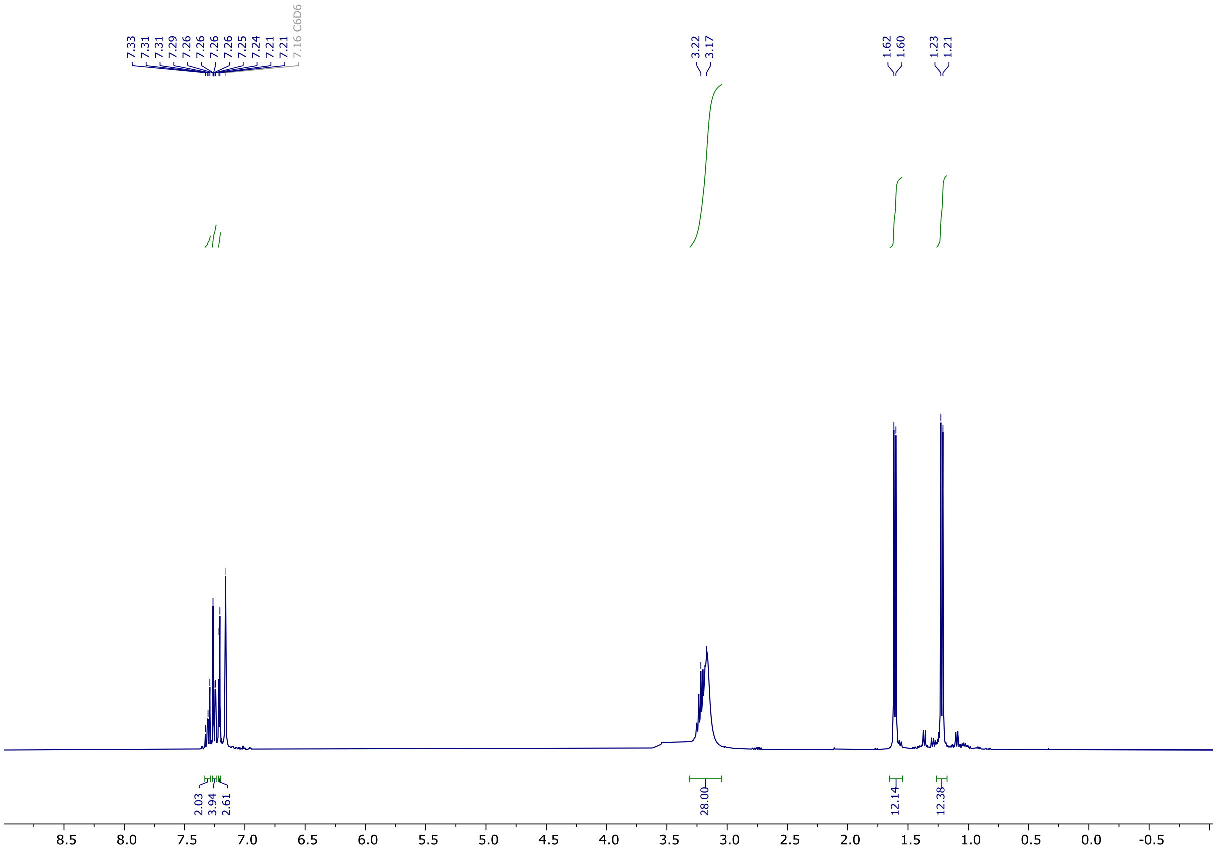

Figure S30: $^{13}C\{^{1}H\}$ NMR spectrum of the cyanide isomer of $[^{Dipp}TerPO_2(CN)][K(18\text{-crown-6})]$ after heating a isomer mixture in the solid state at 110 °C (101 MHz, C_6D_6, rt).

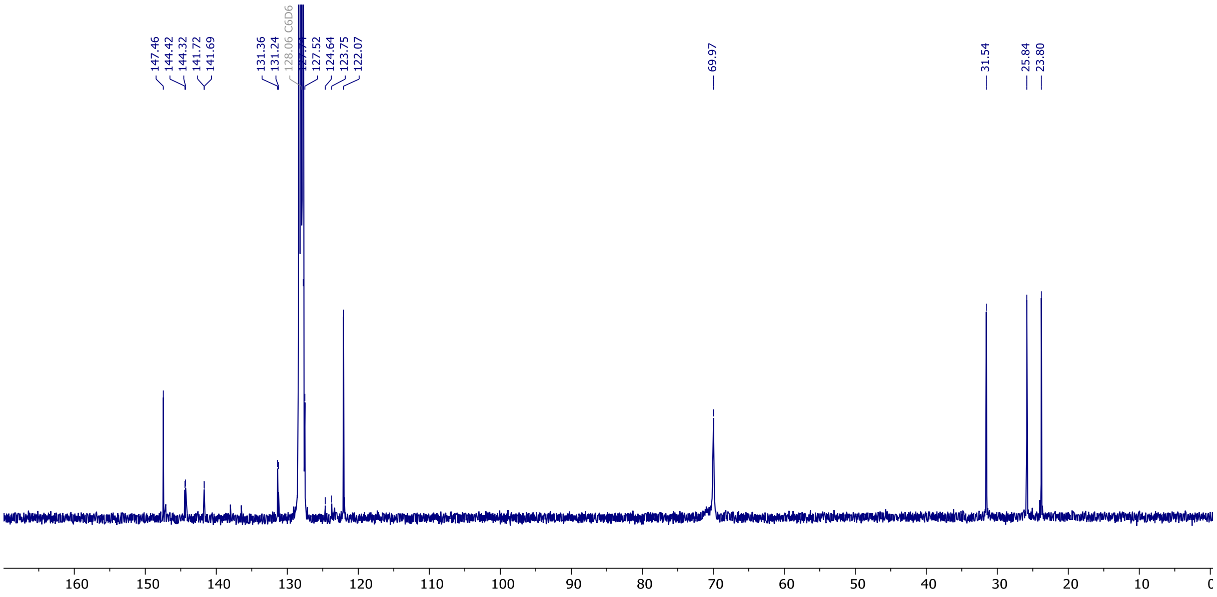

Figure S31: ^{31}P NMR spectrum of the cyanide isomer of $[^{Dipp}TerPO_2(CN)][K(18\text{-crown-6})]$ after heating a isomer mixture in the solid state at 110 °C (162 MHz, C_6D_6, rt); −9.03 ppm $PO_2(NC)$; −140.4 ppm traces of $^{Dipp}TerPH_2$

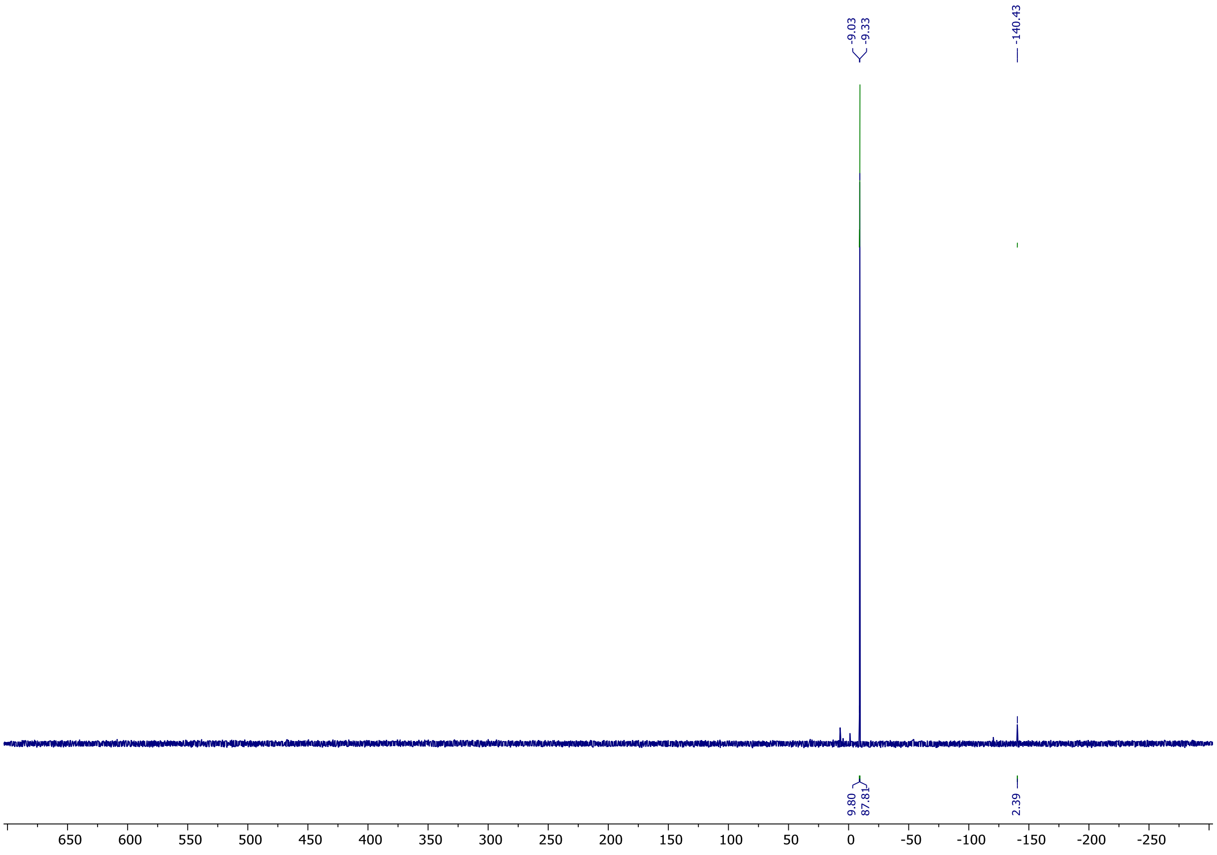

4.4.3 Synthesis of [^{Dipp}TerPO$_2$(CN)][K(18-crown-6)] in the solid state

In a 10 mL Schlenk flask [^{Dipp}TerPCN][K(18-c-6)] salt (16 mg, 0.02 mmol) was added, and the evacuated flask was filled with 1 atm of dried air (passed through Sicapent®). The reaction was carried out in a neat condition and the solid was stirred for 3 hours 30 minutes at ambient temperature. As the reaction progresses, the initially bright yellow solid was slowly faded to pale yellow and then white solid. Afterwards, the solid were dissolved in C_6D_6 and NMR spectra were recorded, revealing mixture of PNC-isomer and PCN isomer. Minimal amount of [^{Dipp}TerPCN][K(18-c-6)] salt are observed as it is not fully converted to [^{Dipp}TerPO$_2$(CN)][K(18-c-6)].

^{31}P{^{1}H} NMR (C_6D_6, 162.01 MHz): δ = −9.3 (*P*CN), −9.0 (*P*NC). **^{1}H NMR** (C_6D_6, 300.2 MHz): δ = 1.22 (d, $^{3}J_{H,H}$ = 6.9 Hz, 12 H, CH(C*H*$_3$)$_2$), 1.61 (d, ^{3}J = 6.7 Hz, 12 H, CH(C*H*$_3$)$_2$), 3.16 (s, 24 H, 18-c-6), 3.22 (sept, $^{3}J_{H,H}$ = 6.9 Hz, 4 H, C*H*(CH$_3$)$_2$), 7.20-7.24 (m, 4H, *m*-C*H*$_{DipTer}$, *p*-C*H*$_{DipTer}$), 7.26-7.30 (m, 4 H, *m*-C*H*$_{Dip}$), 7.31-7.35 (m, 1 H, *p*-C*H*$_{Dip}$).

Figure S32: ^{1}H NMR spectrum of the cyanide isomer of [^{Dipp}TerPO$_2$(CN)][K(18-crown-6)] in the solid state (300.2 MHz, C_6D_6, rt).

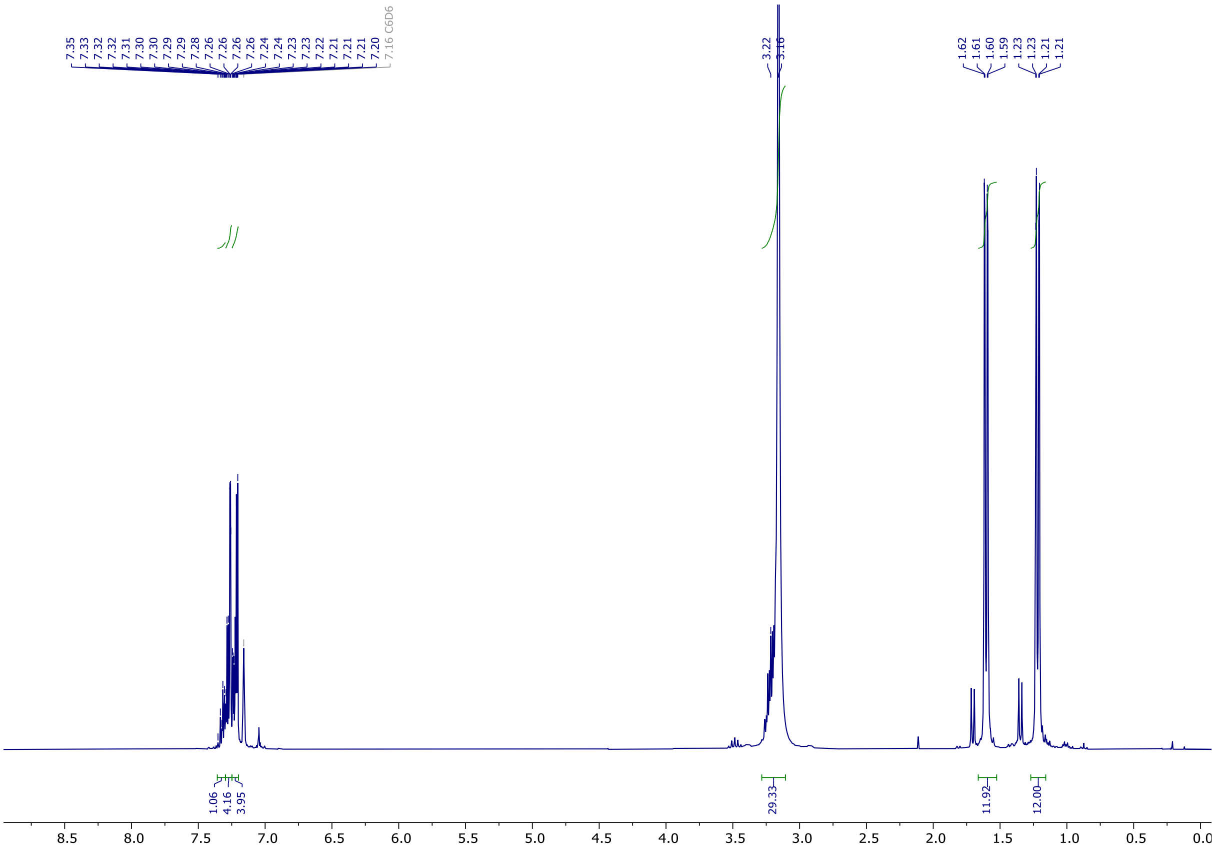

Figure S33: ^{31}P NMR spectrum of the cyanide isomer of [^{Dipp}TerPO$_2$(CN)][K(18-crown-6)] in the solid state (162 MHz, C_6D_6, rt); −9.03 ppm PO$_2$(NC);−127.1 ppm traces of [^{Dipp}TerCN][K(18-crown-6)]

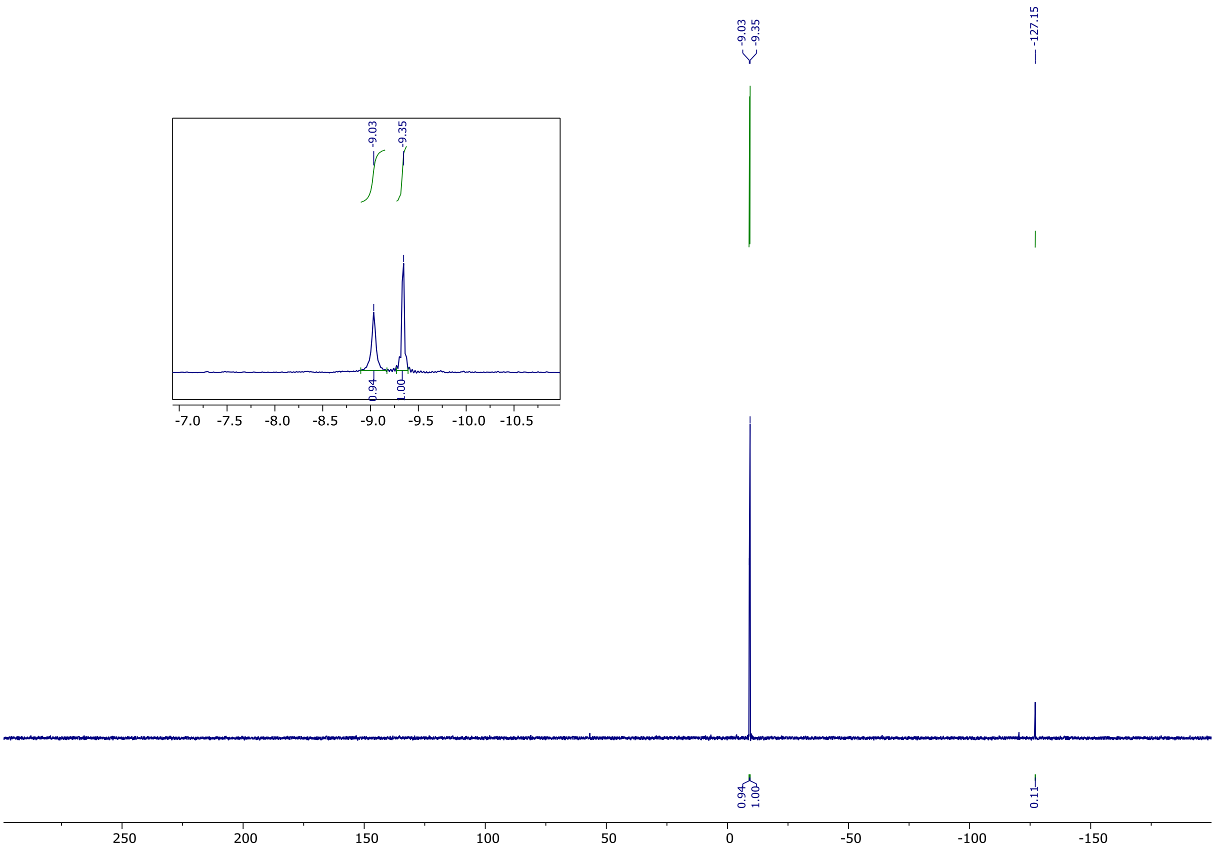

4.5 [^{Dipp}TerPS$_2$(CN)][K(18-crown-6)] (2·S$_2$)

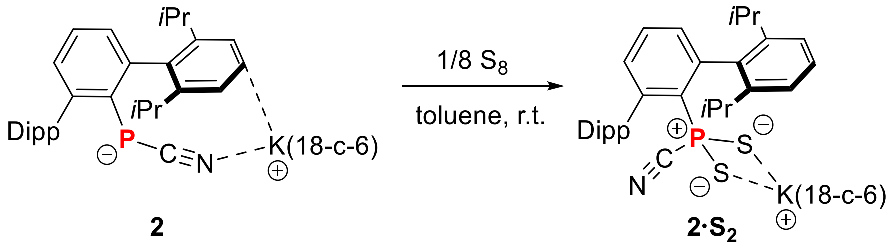

[^{Dipp}TerPS$_2$(CN)][K(18-c-6)] can be synthesized directly from ^{Dipp}TerP(H)CN. In a 10 mL Schlenk flask ^{Dipp}TerP(H)CN (50 mg, 0.11 mmol), KH (5 mg, 0.12 mmol), 18-crown-6 (29 mg, 0.11 mmol) and Sulphur (7 mg, 0.22 mmol) were suspended in toluene (2.5 mL). The reaction mixture was stirred overnight for the in situ generation of [^{Dipp}TerPS$_2$(CN)][K(18-c-6)]. The colour of reaction mixture changed from light orange to pale yellow. Afterwards, all volatile components were removed *in vacuo* (1×10^{-3} mbar), the residue was re-dissolved in fresh toluene (3 mL) and insoluble solids were removed by cannula filtration. The clear filtrate was concentrated *in vacuo* (1×10^{-3} mbar) and stored at −30 °C, resulting in the deposition of white crystals. The supernatant was transferred to another flask and was used to get further fractions of crystalline product. The isolated crystals were dried *in vacuo* (1×10^{-3} mbar) for 1 hours at ambient temperature giving [(^{Dipp}TerPCN)K(18-crown-6)] (**2.S2**) as a yellow crystalline solid. Yield: 48 mg (0.06 mmol, 55%).

CHN calc. (found) in %: C 62.82 (65.21), H 7.48 (7.28), N 1.70 (1.44), S 7.80 (7.23). **^{31}P{^{1}H} NMR** (C_6D_6, 121.5 MHz): δ = 32.3. **^{1}H NMR** (C_6D_6, 400.1 MHz): δ = 1.18 (d, $^3J(^1H,^1H)$ = 6.8 Hz, 12 H, CH(C*H*$_3$)$_2$), 1.71 (d, $^3J(^1H,^1H)$ = 6.7 Hz, 12 H, CH(C*H*$_3$)$_2$), 3.12 (s, 24 H, 18-c-6), 3.38 (sept, $^3J(^1H,^1H)$ = 6.8 Hz, 4 H, C*H*(CH$_3$)$_2$), 7.10-7.13 (m, 1 H, *p*-C*H*$_{DipTer}$), 7.18-7.24 (m, 2 H, *m*-C*H*$_{DipTer}$), 7.25-7.30 (m, 4 H, *m*-C*H*$_{Dip}$), 7.35-7.41 (m, 2 H, *p*-C*H*$_{Dip}$). **^{13}C{^{1}H} NMR** (C_6D_6, 101 MHz): δ = 24.1 (s, CH(CH$_3$)$_2$), 25.9 (s, CH(CH$_3$)$_2$), 31.7 (s, CH(CH$_3$)$_2$), 70.0 (s, 18-c-6), 122.7 (CH$_{Aryl}$), 123.1 (d, $^1J_{P,C}$ = 52.1 Hz, PC≡N), 125.7 (s, C$_{q,Aryl}$), 127.0 (d, $J_{P,C}$ = 3.1 Hz, CH$_{Aryl}$), 128.6 (d, $J_{P,C}$ = 11.6 Hz, CH$_{Aryl}$), 129.3 (s, C$_{q,Aryl}$), 133.1 (d, $J_{P,C}$ = 11 Hz, CH$_{Aryl}$), 137.9 (s, C$_{q,Aryl}$), 138.8 (s, C$_{q,Aryl}$), 139.8 (s, C$_{q,Aryl}$), 140.2 (d, $J_{P,C}$ = 4.2 Hz, C$_{q,Aryl}$), 140.6 (d, $J_{P,C}$ = 12.1 Hz, C$_{q,Aryl}$), 148.5 (s, C$_{q,Aryl}$). **IR** (ATR, 64 scans,

cm^{-1}): $\tilde{\nu}$ = 3055 (vw), 2954 (w), 2860 (w), 2360 (w), 2337 (vw), 1458 (w), 1375 (w), 1350 (m), 1282 (vw), 1252 (w), 1238 (w), 1134 (w), 1109 (vs), 1057 (w), 962 (m), 820 (w), 804 (w), 793 (w), 762 (w), 748 (m), 731 (m), 696 (w), 675 (s), 611 (m), 575 (w), 559 (w), 536 (m), 465 (w), 424 (w). **Raman** (633 nm, 30 s, 30 scans, cm^{-1}): $\tilde{\nu}$ = 3054 (4), 2916 (6), 2866 (4), 2845 (3), 2810 (2), 2154 (3), 1591 (3), 1462 (2), 1334 (2), 1277 (7), 1248 (2), 1178 (3), 1046 (6), 1002 (10), 871 (5), 786 (4), 705 (3), 608 (4), 538 (7), 467 (5), 334 (3), 286 (3), 248 (4). **MS** (ESI-TOF, pos., m/z, rel. int. > 10%): expected: m/z = 303.1205 [(18-crown-6)K]$^+$; found: m/z = 303.1212, (ESI-TOF, neg., m/z, rel. int. > 10%): expected: m/z = 518.2110 [^{Dip}TerPS$_2$(CN)]$^-$; found: m/z = 518.2120.

Single crystals of **2·S$_2$** suitable for X-ray diffraction can be grown from saturated toluene solution at −30 °C.

Figure S34: ^{1}H NMR spectrum of **[^{Dipp}TerPS$_2$(CN)][K(18-crown-6)]** (400.1 MHz, C_6D_6, rt).

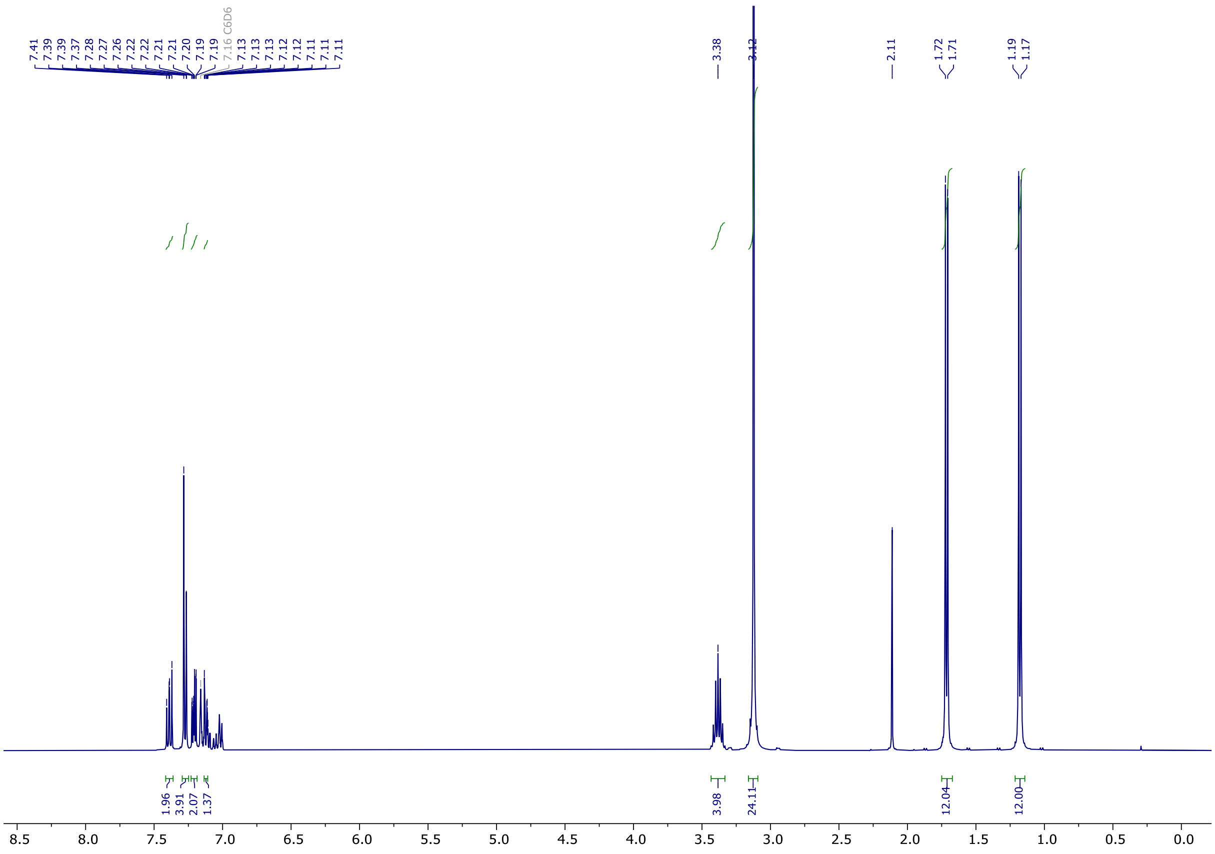

Figure S35: $^{13}C\{^1H\}$ NMR spectrum of **[^{Dipp}TerPS$_2$(CN)][K(18-crown-6)]** (101 MHz, C_6D_6, rt).

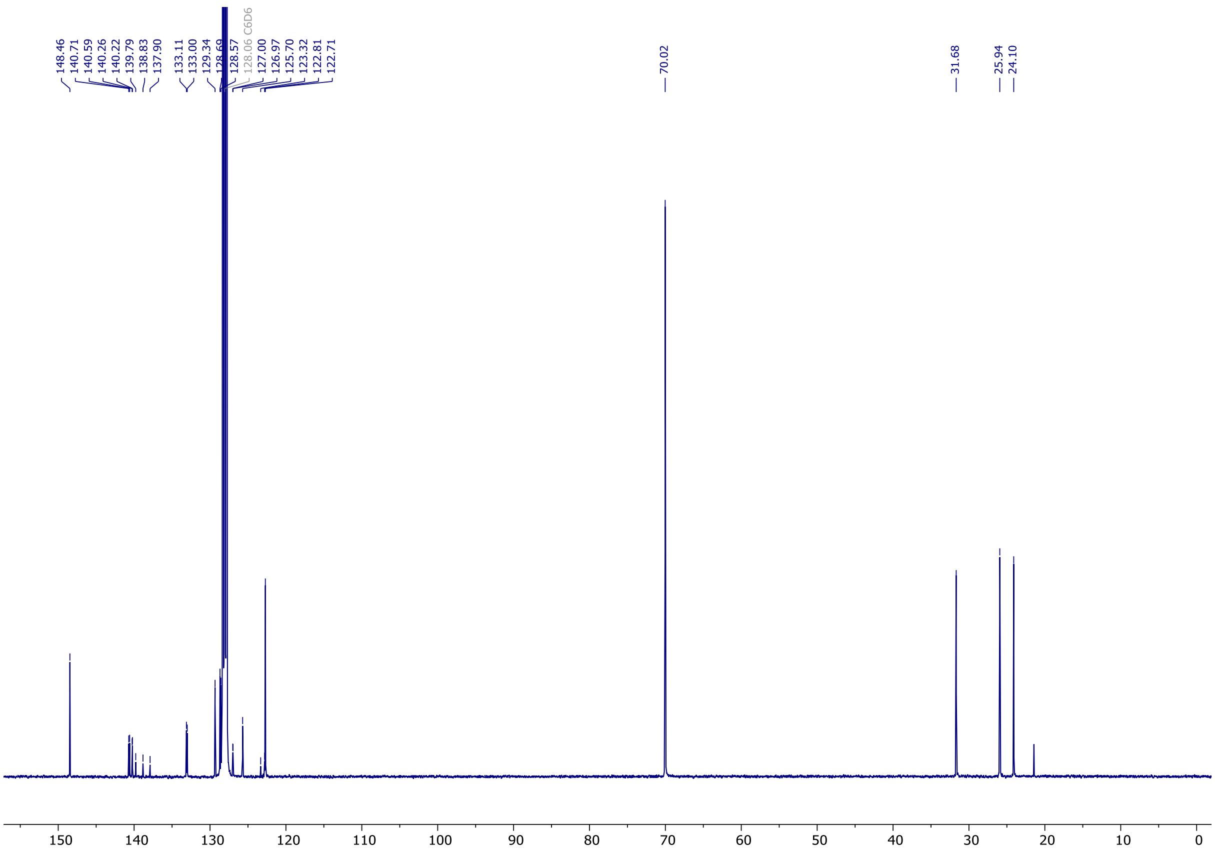

Figure S36: $^{31}P\{^1H\}$ NMR spectrum of **[^{Dipp}TerPS$_2$(CN)][K(18-crown-6)]** (121.5 MHz, C_6D_6, rt).

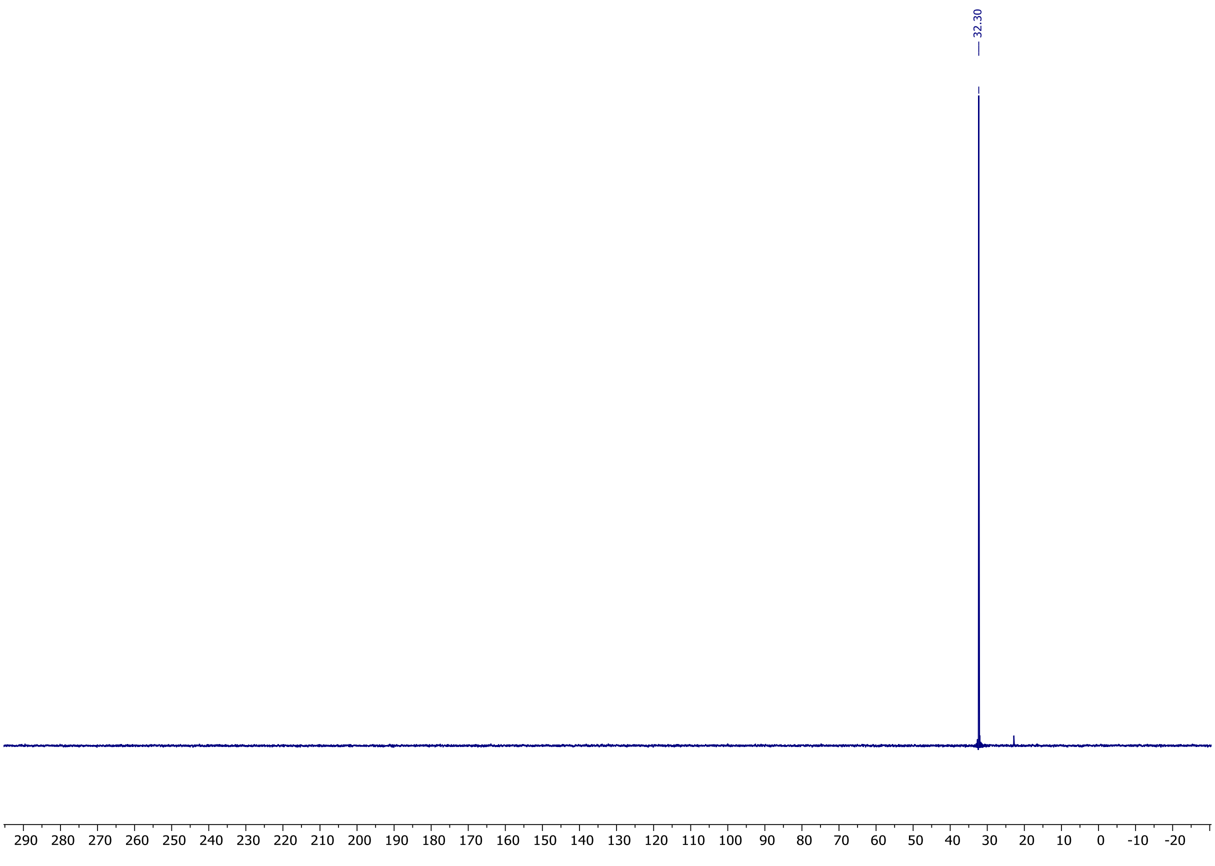

Figure S37: IR (ATR, solid sample, 64 scans, cm^{-1}) and Raman (633 nm, 30 s, 30 scans, cm^{-1}) spectra of isolated crystals of **[^{Dipp}TerPS$_2$(CN)][K(18-crown-6)]**.

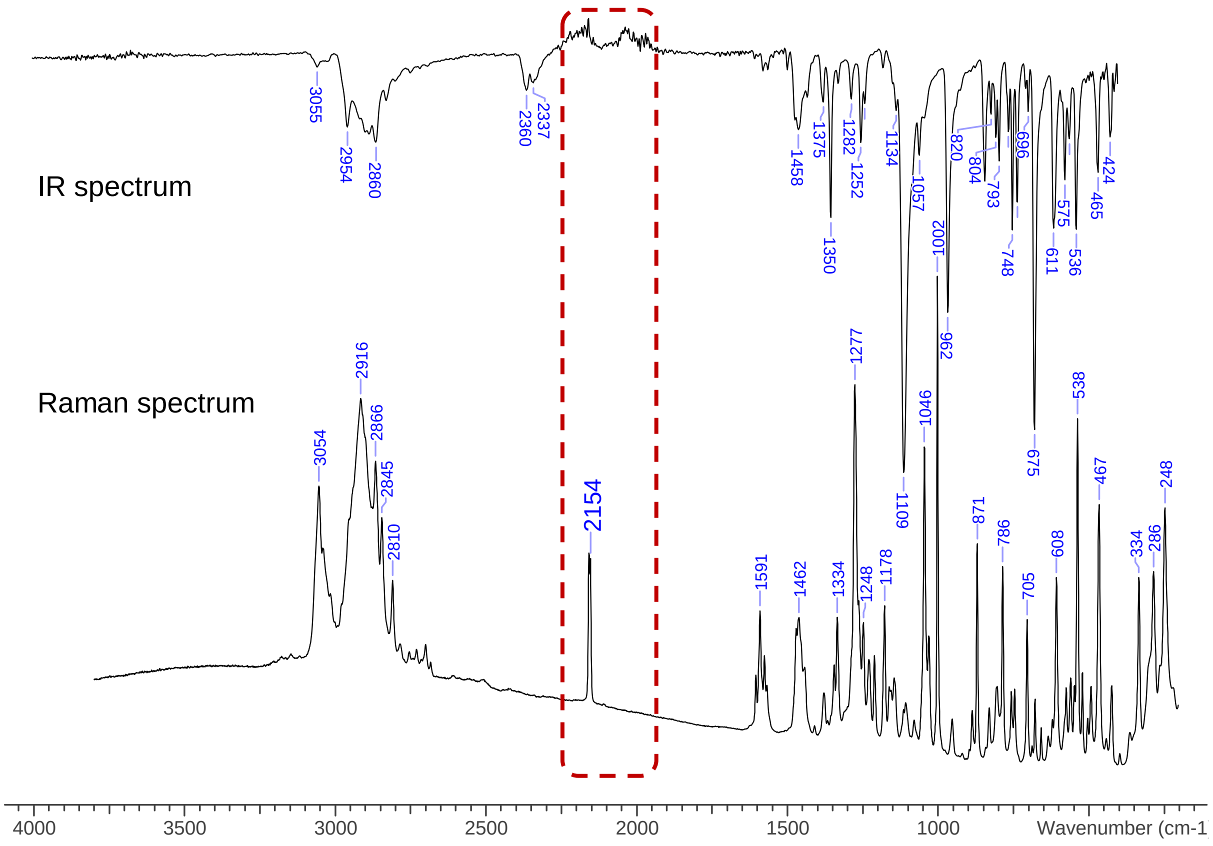

4.6 [^{Dipp}TerPO$_2$(CN)][Na(18-crown-6)] (3·O$_2$)

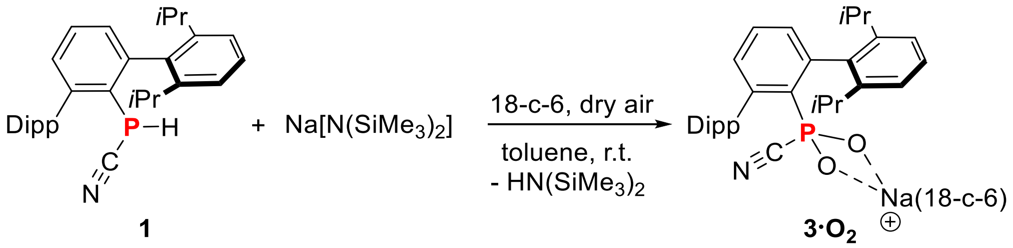

In a 10 mL Schlenk flask 18-crown-6 (30 mg, 0.11 mmol), ^{Dipp}TerP(H)CN (50 mg, 0.11 mmol) and NaHMDS (22 mg, 0.12 mmol) were dissolved in toluene (2.5 mL) and stirred overnight. Then the mixture was degassed via three freeze-pump-thaw cycles. The evacuated flask was filled with 1 atm of dried air (passed through Sicapent®) and the reaction mixture was stirred for 15 minutes at ambient temperature. As the reaction progresses, the initially bright yellow suspension immediately changes into orange then purple. The deep purple colour stayed for 5 minutes and then faded to pink then light pink and finally pale-yellow. Further stirring for 25 minutes shows that the pale-yellow solution remained unchanged. Then all volatile components were removed *in vacuo* (1×10^{-3} mbar). The residue was redissolved in toluene (5 mL) and the clear filtrate was concentrated *in vacuo* (1×10^{-3} mbar) and stored at −30 °C, resulting in the deposition of colourless crystals. The supernatant was transferred to another flask and was used to get further fractions of crystalline product. The isolated crystals were dried *in vacuo* (1×10^{-3} mbar) for 2 hours in a water bath (30 °C), yielding [^{Dipp}TerPO$_2$(CN)][Na(18-crown-6)] (**3·O$_2$**) as a colourless crystalline solid. Yield: 41 mg (0.05 mmol, 45%).

CHN calc. (found) in %: C 66.73 (67.20), H 7.94 (7.69), N 1.81 (1.65). **^{31}P{^{1}H} NMR** (C_6D_6, 121.5 MHz): δ = −8.5, −9.3. **^{1}H NMR** (C_6D_6, 300.2 MHz): δ = 1.19 (dd, $^{3}J(^{1}H,^{1}H)$ = 6.9, 1.2 Hz, 12 H, CH(CH_3)$_2$),* 1.57 (d, $^{3}J(^{1}H,^{1}H)$ = 6.8 Hz, 12 H, CH(CH_3)$_2$), 3.16 (sept, $^{3}J(^{1}H,^{1}H)$ = 6.9 Hz, 4 H, C*H*(CH$_3$)$_2$),* 3.18 (s, 24 H, 18-c-6), 7.19-7.31 (m, 9 H, CH_{Aryl}) *overlapping signals of the PCN and PNC isomers. **^{13}C{^{1}H} NMR** (C_6D_6, 75.5 MHz): δ = 23.9 (s, PCN, CH(CH$_3$)$_2$), 24.1 (s, PNC, CH(CH$_3$)$_2$), 25.7 (s, PNC, CH(CH$_3$)$_2$), 25.8 (s, PCN, CH(CH$_3$)$_2$), 31.4 (s, PNC, CH(CH$_3$)$_2$), 31.5 (s, PCN, CH(CH$_3$)$_2$), 69.3 (s, 18-c-6), 127.1

(s, CH$_{Aryl}$), 127.3 (s, CH$_{Aryl}$), 127.9 (d, $J_{P,C}$ = 4.0 Hz, CH$_{Aryl}$)*, 128.2 (d, $J_{P,C}$ = 4.4 Hz, CH$_{Aryl}$)*, 131.5 (d, $J_{P,C}$ = 6.9 Hz, CH$_{Aryl}$), 131.7 (d, $J_{P,C}$ = 5.9 Hz, CH$_{Aryl}$), 142.1 (d, $J_{P,C}$ = 3.4 Hz, $C_{q,Aryl}$), 142.7 (d, $J_{P,C}$ = 3.2 Hz, $C_{q,Aryl}$), 145.2 (d, $J_{P,C}$ = 9.8 Hz, $C_{q,Aryl}$), 145.5 (d, $J_{P,C}$ = 9.8 Hz, $C_{q,Aryl}$), 146.9 (s, $C_{q,Aryl}$), 147.1 (s, $C_{q,Aryl}$) *overlap with C_6D_6 signal. **IR** (ATR, 64 scans, cm^{-1}): $\tilde{\nu}$ = 2954 (w), 2864 (w), 2083 (m), 1577 (vw), 1560 (w), 1458 (w), 1379 (w), 1354 (m), 1325 (vw), 1271 (m), 1252 (m), 1138 (m), 1099 (vs), 1045 (m), 957 (m), 835 (w), 823 (w), 804 (m), 795 (m), 750 (s), 708 (w), 613 (m), 580 (s), 561 (w), 536 (s), 505 (w), 459 (w). **Raman** (633 nm, 30 s, 30 scans, cm^{-1}): $\tilde{\nu}$ = 3064 (2), 3035 (3), 2964 (4), 2928 (7), 2908 (8), 2864 (7), 2175 (3), 2084 (3), 1592 (4), 1577 (2), 1460 (3), 1332 (2), 1276 (10), 1227 (2), 1138 (2), 1093 (4), 1044 (5), 1002 (3), 885 (4), 807 (2), 748 (2), 611 (7), 338 (2), 279 (3). **MS** (ESI-TOF, pos., m/z, rel. int. > 10%): expected: m/z = 287.1466 [(18-crown-6)Na]$^+$; found: m/z = 287.1458, (ESI-TOF, neg., m/z, rel. int. > 10%): expected: m/z = 486.2567 [^{Dip}TerPO$_2$(CN)]$^-$; found: m/z = 486.2576.

Single crystals of **3·O₂** suitable for X-ray diffraction can be grown from saturated toluene solution at −30 °C.

Figure S38: ^{1}H NMR spectrum of **[^{Dipp}TerPO$_2$(CN)][Na(18-crown-6]** (300.2 MHz, C_6D_6, rt).

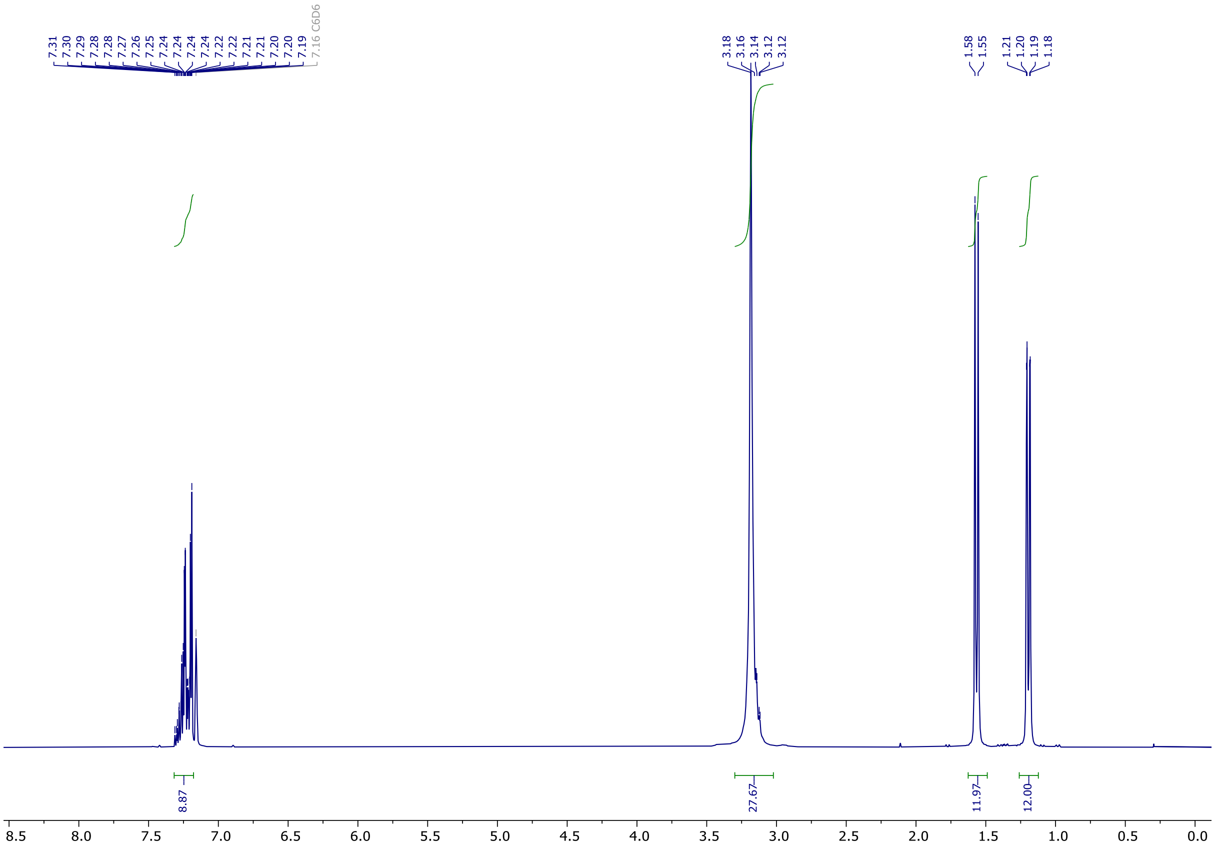

Figure S39: $^{13}C\{^1H\}$ NMR spectrum of **[$^{Dipp}TerPO_2(CN)$][Na(18-crown-6)]** (75.5 MHz, C_6D_6, rt).

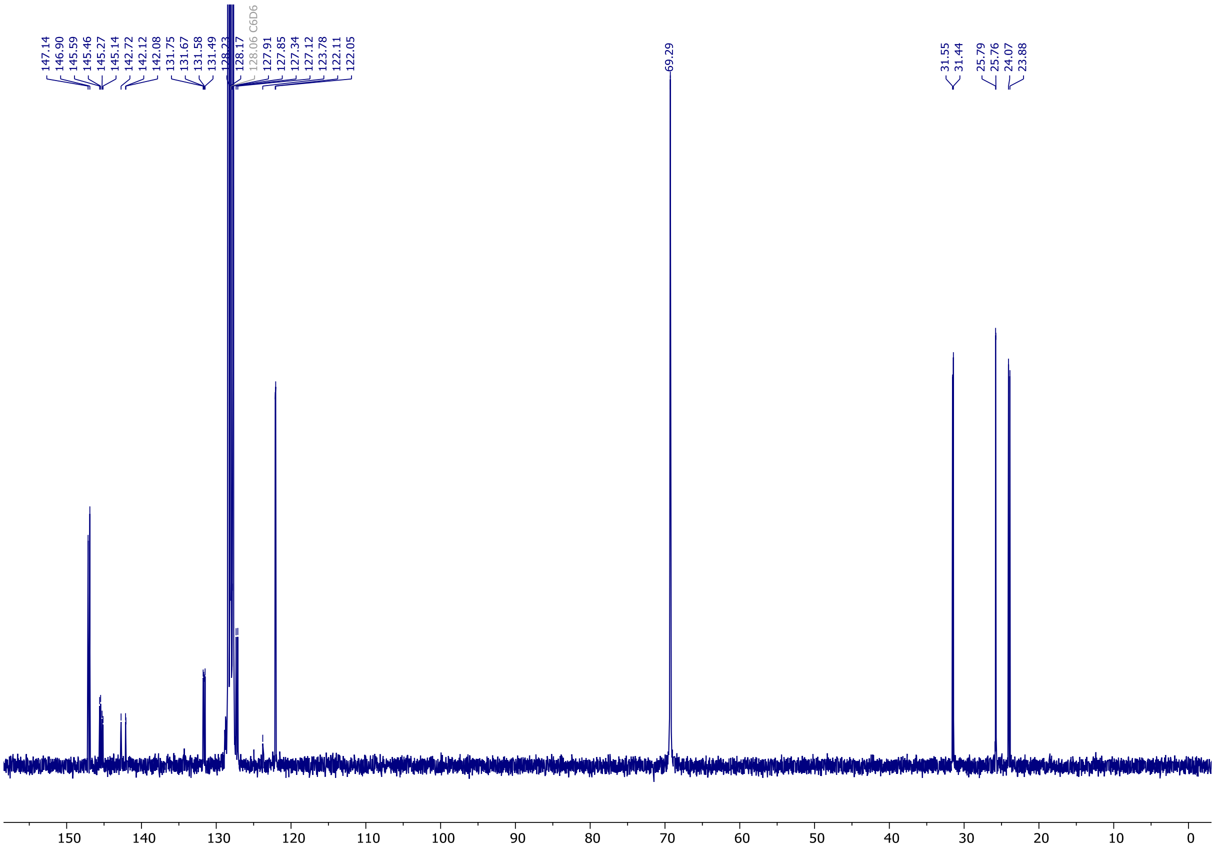

Figure S40: $^{31}P\{^1H\}$ NMR spectrum of **[$^{Dipp}TerPO_2(CN)$][Na(18-crown-6)]** (122 MHz, C_6D_6, rt).

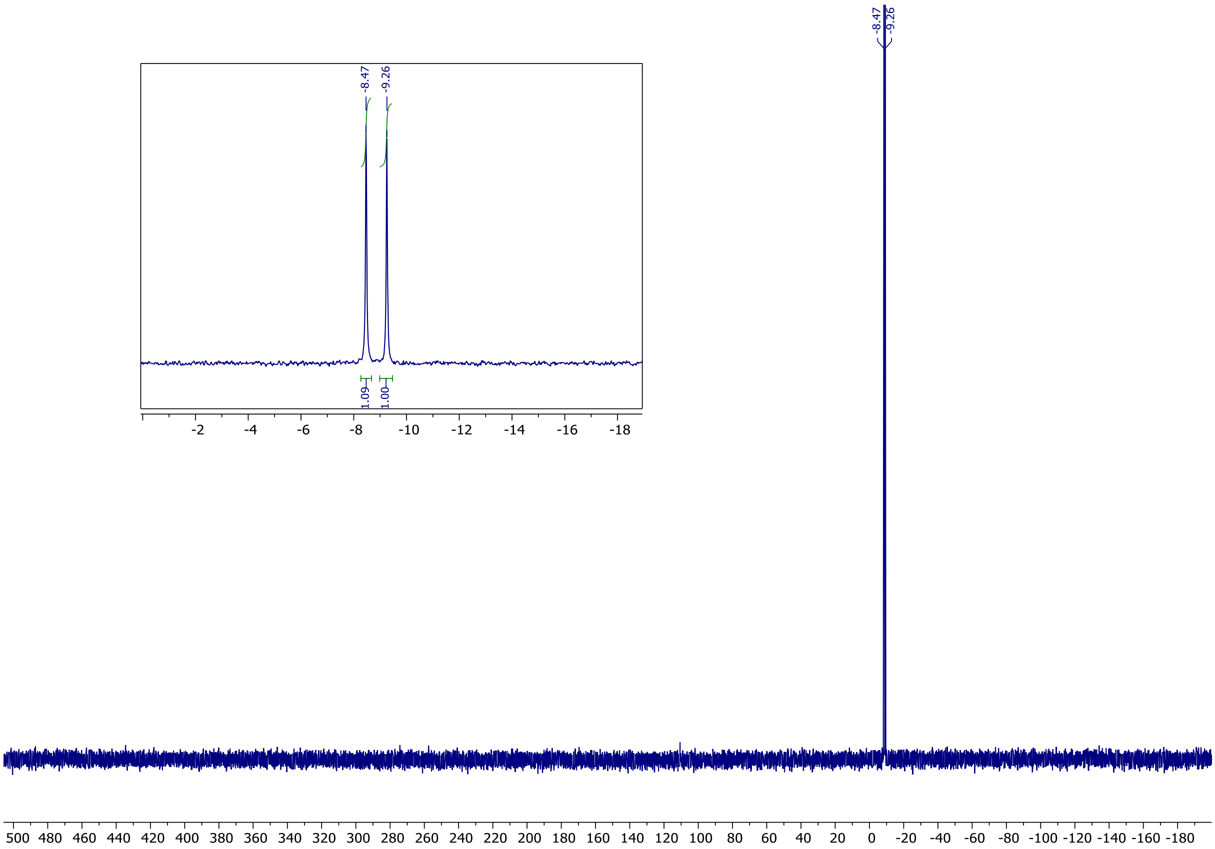

Figure S41: IR (ATR, solid sample, 64 scans, cm^{-1}) and Raman (633 nm, 30 s, 30 scans, cm^{-1}) spectrums of isolated crystals of **[^{Dipp}TerPO$_2$(CN)][Na(18-crown-6)]**.

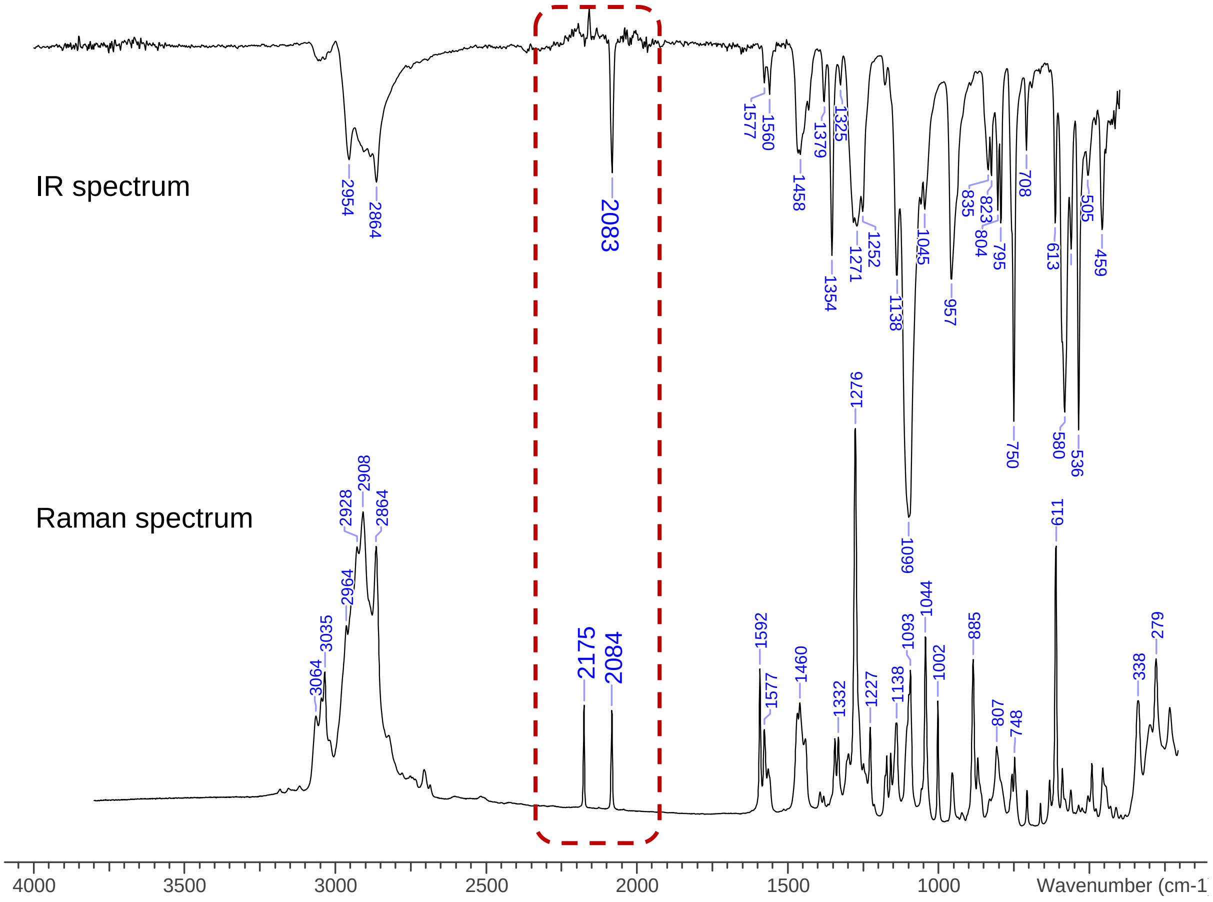

4.6.1 Heating of [^{Dipp}TerPO$_2$(CN)][Na(18-crown-6)] in solution at 80 °C

In a Young NMR tube [^{Dip}TerPO$_2$(CN)][Na(18-c-6)] (11 mg, 0.01 mmol) (as a mixture of PCN and PNC isomers) was dissolved in C_6D_6 (0.6 mL) and was heated in an oil bath at 80 °C overnight. Single crystals suitable for X-ray diffraction can be grown from a saturated toluene solution at −30 °C, yielding white block-shaped crystals of **3·O₂**. Yield: 7.5 mg (0.01 mmol, 68%). The NMR data was recorded after recrystallization showing that the tentative isocyanide isomer was completely transformed into the cyanide isomer (δ(^{31}P) = −9.27 ppm).

^{31}P{^{1}H} NMR (C_6D_6, 121.5 MHz): δ = −9.27. **^{1}H NMR** (C_6D_6, 300.2 MHz): δ = 1.19 (d, $^3J(^1H,^1H)$ = 6.8 Hz, 12 H, CH(CH_3)$_2$), 1.57 (d, $^3J(^1H,^1H)$ = 6.8 Hz, 12 H, CH(CH_3)$_2$), 3.17* (sept, $^3J(^1H,^1H)$ = 6.8 Hz, 4 H, CH(CH$_3$)$_2$), 3.18* (s, 24 H, 18-c-6), 7.17-7.20 (m, 3 H, *m*-CH_{DipTer}, *p*-CH_{DipTer}), 7.20-7.24 (m, 4 H, *m*-CH_{Dip}), 7.24-7.31 (m, 2 H, *p*-CH_{Dip}).*Overlapping signals. **^{13}C{^{1}H} NMR** (C_6D_6, 101 MHz): δ = 23.5 (s, PCN, CH(CH$_3$)$_2$), 25.8 (s, CH(CH$_3$)$_2$), 31.5 (s, PCN, CH(CH$_3$)$_2$), 69.3 (s, 18-c-6), 122.1 (s, CH$_{Aryl}$), 124.4 (d, $^1J_{P,C}$ = 89.7 Hz, PC≡N), 127.3 (s, CH$_{Aryl}$), 127.9 (s, CH$_{Aryl}$)*, 128.4 (s, CH$_{Aryl}$)*, 131.7 (d, $J_{P,C}$ = 12.8 Hz, CH$_{Aryl}$), 142.1 (d, $J_{P,C}$ = 3.2 Hz, $C_{q,Aryl}$), 145.2 (d, $J_{P,C}$ = 9.8 Hz, $C_{q,Aryl}$), 147.1 (s, $C_{q,Aryl}$) *overlap with C_6D_6 signal. **IR** (ATR, 64 scans, cm^{-1}): $\tilde{\nu}$ = 3049 (vw), 2954 (w), 2864 (m), 1577 (w), 1560 (w), 1466 (w), 1458 (w), 1431 (w), 1379 (w), 1354 (m), 1325 (w), 1296 (w), 1263 (m), 1252 (m), 1178 (w), 1138 (m), 1105 (vs), 1093 (vs), 1045 (m), 957 (m), 835 (w), 823 (m), 804 (m), 793 (m), 750 (s), 708 (w), 613 (m), 584 (s), 559 (m), 536 (s), 503 (w), 459 (m), 413 (w). **Raman** (633 nm, 30 s, 30 scans, cm^{-1}): $\tilde{\nu}$ = 2965 (4), 2929 (5), 2907 (6), 2864 (6), 2176 (10), 1593 (3), 1466 (3), 1277 (7), 1227 (3), 1093 (4), 1044 (3), 1003 (4), 885 (4), 807 (3), 612 (8), 280 (3), 234 (2).

Figure S42: ^{1}H NMR spectrum of the cyanide isomer of **[^{Dipp}TerPO$_2$(CN)][Na(18-crown-6)]** (300.2 MHz, C_6D_6, rt).

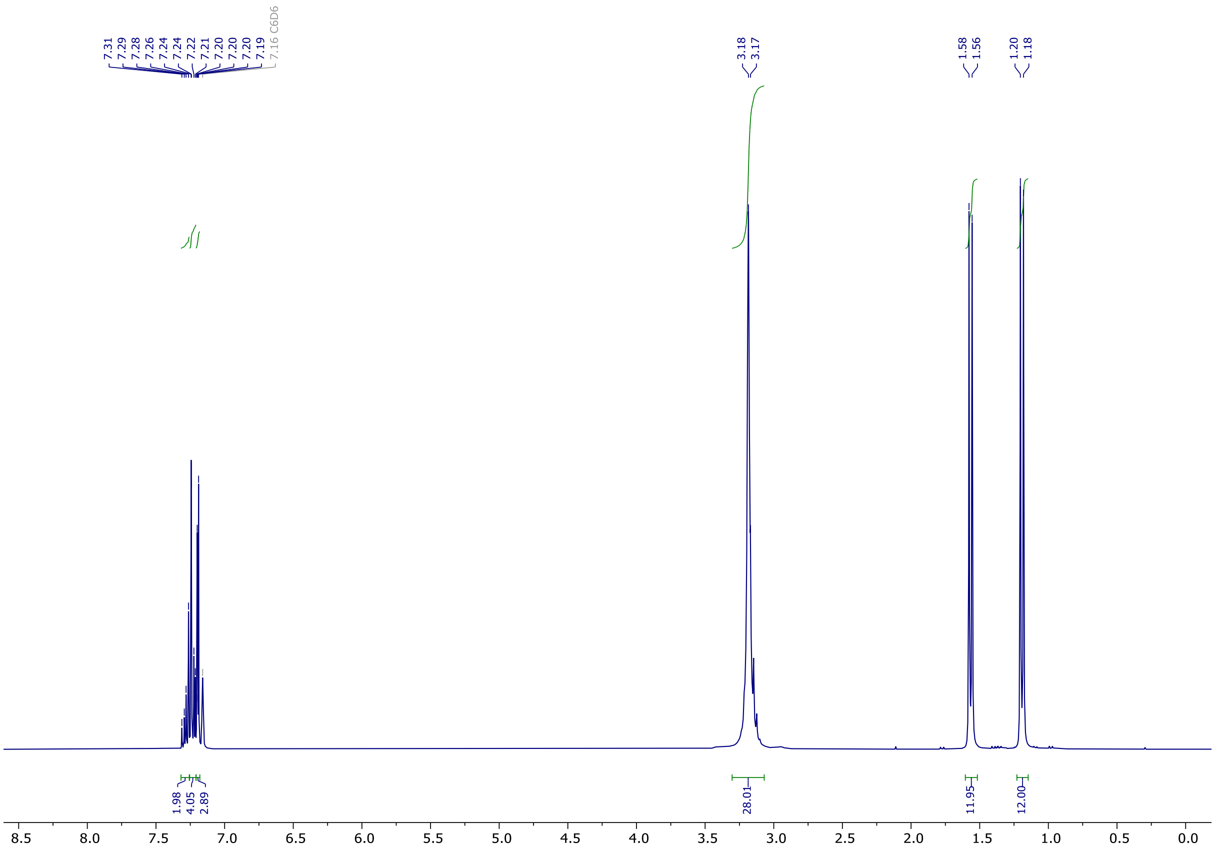

Figure S43: $^{13}C\{^1H\}$ NMR spectrum of **[$^{Dipp}TerPO_2(CN)$][Na(18-crown-6)]** (75.5 MHz, C_6D_6, rt).

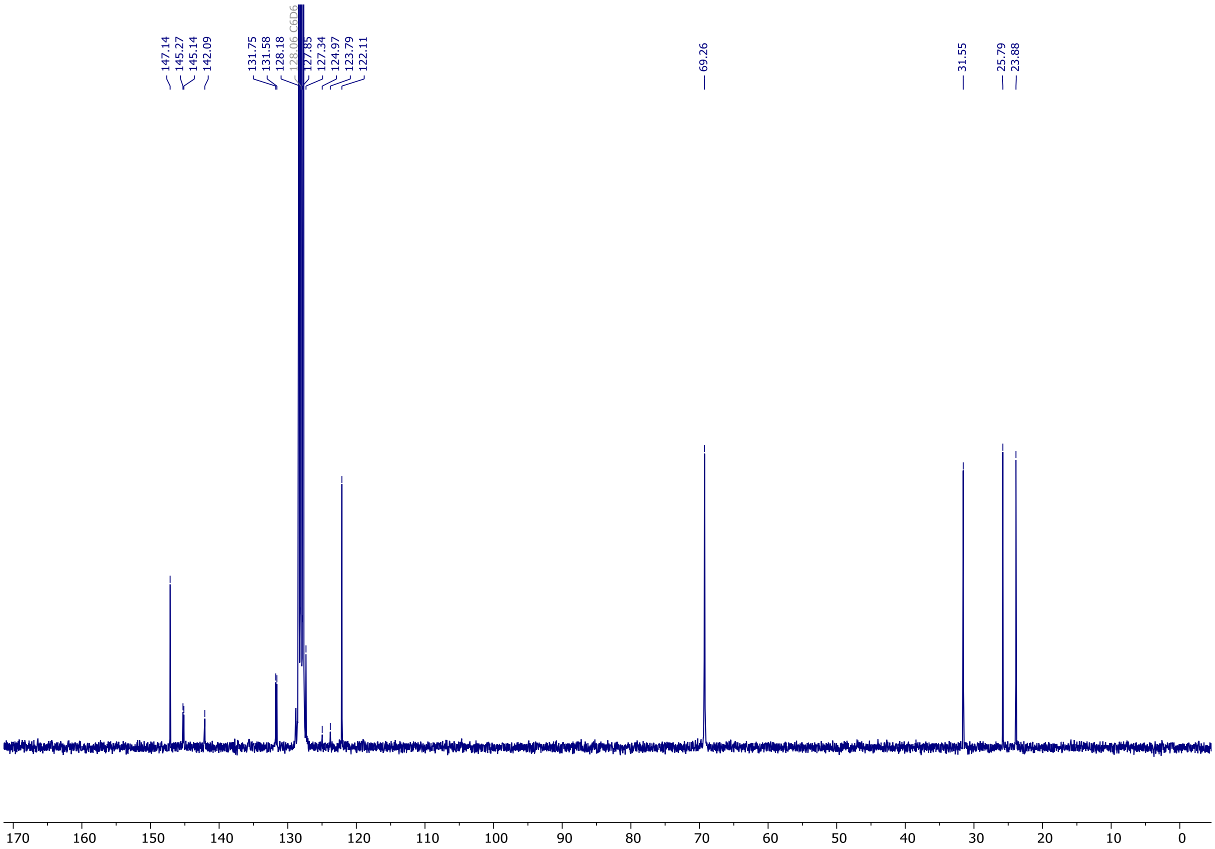

Figure S44: ^{31}P NMR spectrum of the cyanide isomer of **[^{Dipp}TerPO$_2$(CN)][Na(18-crown-6)]** (121.5 MHz, C_6D_6, rt).

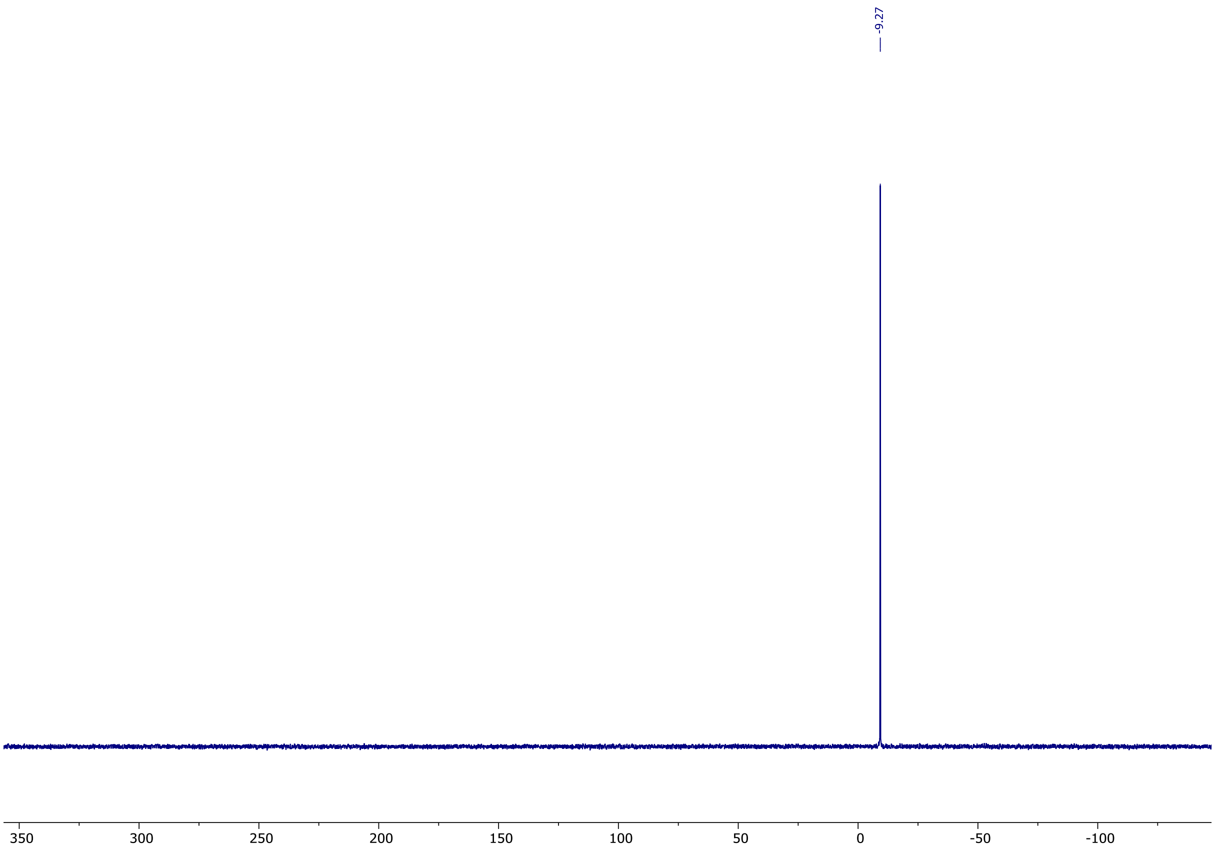

Figure S45: IR (ATR, solid sample, 64 scans, cm^{-1}) and Raman (633 nm, 30 s, 30 scans, cm^{-1}) spectra of isolated crystals of **[^{Dip}TerPO$_2$(CN)][Na(18-crown-6)]** after heating a C_6D_6 solution of **3·O$_2$** to 80 °C.

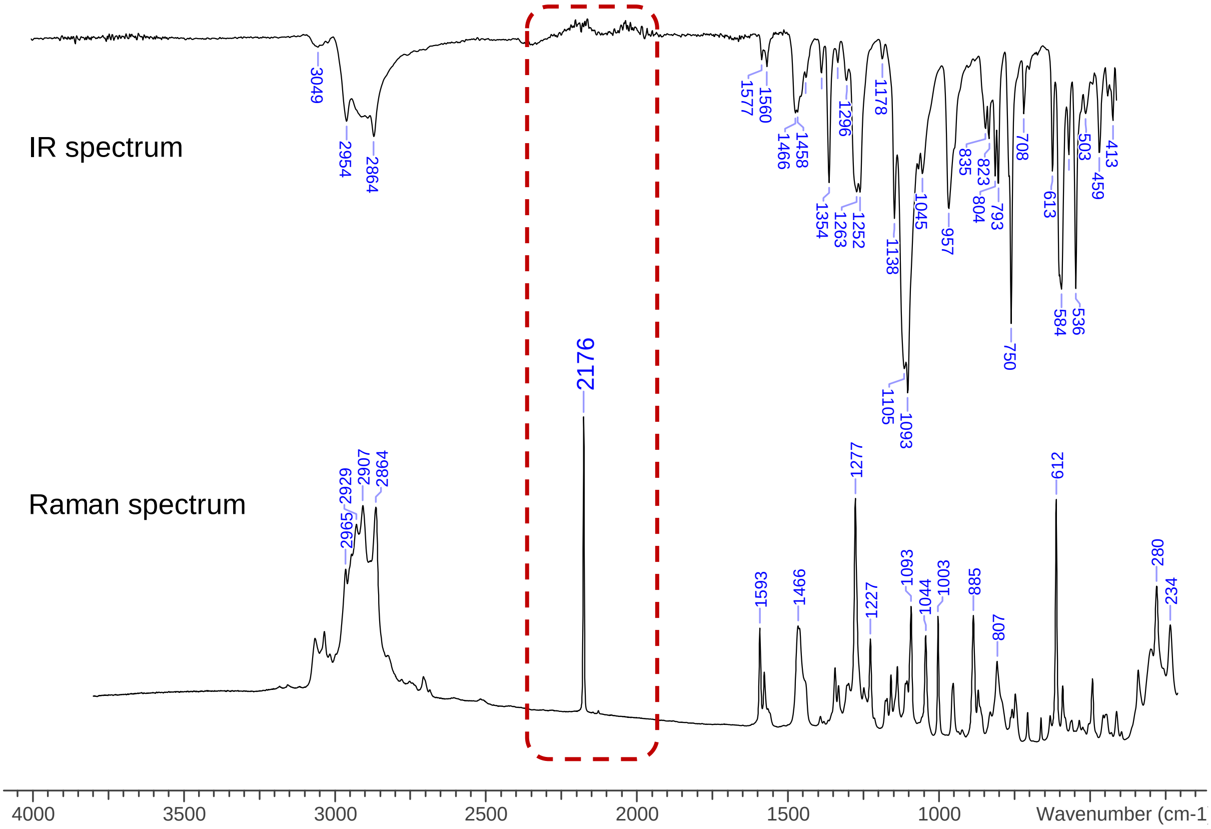

4.7 [^{Dipp}TerPO$_2$(CN)][Na(15-crown-5)] (4·O$_2$)

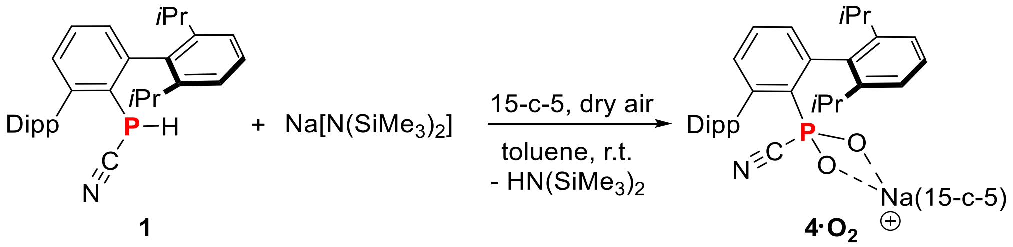

In a 10 mL Schlenk flask 15-crown-5 (20 mg, 0.09 mmol), ^{Dipp}TerP(H)CN (40 mg, 0.09 mmol) and NaHMDS (17 mg, 0.09 mmol) were dissolved in toluene (2 mL) and then the mixture was degassed via three freeze-pump-thaw cycles. The evacuated flask was filled with 1 atm of dried air (passed through Sicapent®) and the reaction mixture was stirred for 15 minutes at ambient temperature. As the reaction progresses, the initially bright yellow suspension immediately changes into orange then purple. The deep purple colour stayed for 5 minutes and then faded to pink then light pink and finally pale-yellow. Further stirring for 25 minutes shows that the pale-yellow solution remained unchanged. Then all volatile components were removed *in vacuo* (1×10^{-3} mbar). The residue was redissolved in toluene (5 mL) and the clear filtrate was concentrated *in vacuo* (1×10^{-3} mbar) and stored at −30 °C, resulting in the deposition of colourless crystals. The supernatant was transferred to another flask and was used to get further fractions of crystalline product. The isolated crystals were dried *in vacuo* (1×10^{-3} mbar) for 2 hours in a water bath (30 °C), yielding [^{Dipp}TerPO$_2$(CN)][Na(15-crown-5)] (**4·O$_2$**) as a colourless crystalline solid. Yield: 31 mg (0.04 mmol, 49%).

CHN calc. (found) in %: C 67.47 (66.53), H 7.87 (6.88), N 1.92 (1.54). **^{31}P{^{1}H} NMR** (C_6D_6, 162 MHz): δ = −8.33. **^{1}H NMR** (C_6D_6, 400.1 MHz): δ = 1.20 (d, $^3J(^1H,^1H)$ = 6.9 Hz, 12 H, CH(CH_3)$_2$),* 1.61 (d, $^3J(^1H,^1H)$ = 6.9 Hz, 12 H, CH(CH_3)$_2$), 3.15 (s, 20 H, 15-c-5),* 3.19 (sept, $^3J(^1H,^1H)$ = 6.9 Hz, 4 H, CH(CH$_3$)$_2$),* 7.20-7.22 (m, 3 H, *m*-CH_{DipTer}, *p*-CH_{DipTer}), 7.23-7.31 (m, 6 H, *m*-CH_{Dip}, *p*-CH_{Dip}). *overlapping signals of the PCN and PNC isomers. **^{13}C{^{1}H} NMR** (C_6D_6, 101 MHz): δ = 23.7 (s, CH(CH$_3$)$_2$), 23.9 (s, CH(CH$_3$)$_2$), 25.80 (s, CH(CH$_3$)$_2$), 25.84 (s, CH(CH$_3$)$_2$), 31.48 (s, CH(CH$_3$)$_2$), 31.57 (s, CH(CH$_3$)$_2$), 68.7 (s, 15-c-5), 122.04 (s, CH$_{Aryl}$), 122.14 (s, CH$_{Aryl}$), 124.0 (d, $^1J_{P,C}$ = 93.5 Hz, PC≡N), 127.2 (s, CH$_{Aryl}$),

127.6 (s, CH_{Aryl}), 128.7 (s, CH_{Aryl}), 131.3 (d, CH_{Aryl}),* 131.5 (d, CH_{Aryl}),* 141.6 (d, $J_{P,C}$ = 3.5 Hz, $C_{q,Aryl}$), 142.5 (d, $J_{P,C}$ = 3.5 Hz, $C_{q,Aryl}$), 144.7 (d, $J_{P,C}$ = 10.0 Hz, $C_{q,Aryl}$), 145.3 (d, $J_{P,C}$ = 3.5 Hz, $C_{q,Aryl}$), 147.1 (s, $C_{q,aryl}$), 147.4 (s, $C_{q,aryl}$). *overlapping signals, $J_{P,C}$ coupling constants cannot be resolved. **IR** (ATR, 32 scans, cm^{-1}): $\tilde{\nu}$ = 3055 (vw), 2960 (m), 2925 (w), 2867 (m), 2080 (w), 1577 (w), 1564 (w), 1461 (m), 1430 (w), 1381 (w), 1352 (m), 1288 (m), 1276 (m), 1249 (m), 1175 (w), 1140 (m), 1103 (vs), 1047 (m), 954 (m), 946 (m), 864 (w), 822 (w), 804 (m), 794 (m), 750 (s), 709 (w), 612 (m), 581 (s), 563 (m), 536 (s), 497 (w), 458 (m), 410 (w). **Raman** (633 nm, 20 s, 10 scans, cm^{-1}): $\tilde{\nu}$ = 3057 (2), 3046 (2), 2928 (5), 2865 (4), 2788 (1), 2752 (1), 2706 (1), 2510 (1), 2175 (2), 2081 (1), 1591 (5), 1577 (2), 1457 (3), 1396 (1), 1348 (2), 1334 (3), 1276 (10), 1247 (1), 1227 (2), 1175 (1), 1159 (1), 1140 (2), 1095 (4), 1046 (4), 1003 (2), 885 (1), 863 (2), 829 (1), 807 (1), 756 (1), 708 (1), 663 (1), 632 (1), 613 (5), 591 (1), 562 (1), 493 (2), 458 (1), 445 (1), 342 (1), 285 (3), 237 (3). **MS** (ESI-TOF, pos., m/z, rel. int. > 10%): expected: m/z = 243.1203 $[(15\text{-crown-}5)Na]^+$; found: m/z = 243.1202, (ESI-TOF, neg., m/z, rel. int. > 10%): expected: m/z = 486.2567 $[^{Dip}TerPO_2(CN)]^-$; found: m/z = 486.2571.

Single crystals of **4·O₂** suitable for X-ray diffraction can be grown from saturated toluene solution at −30 °C.

Figure S46: ^{1}H NMR spectrum of **[^{Dipp}TerPO$_2$(CN)][Na(15-crown-5]** (400.1 MHz, C_6D_6, rt).

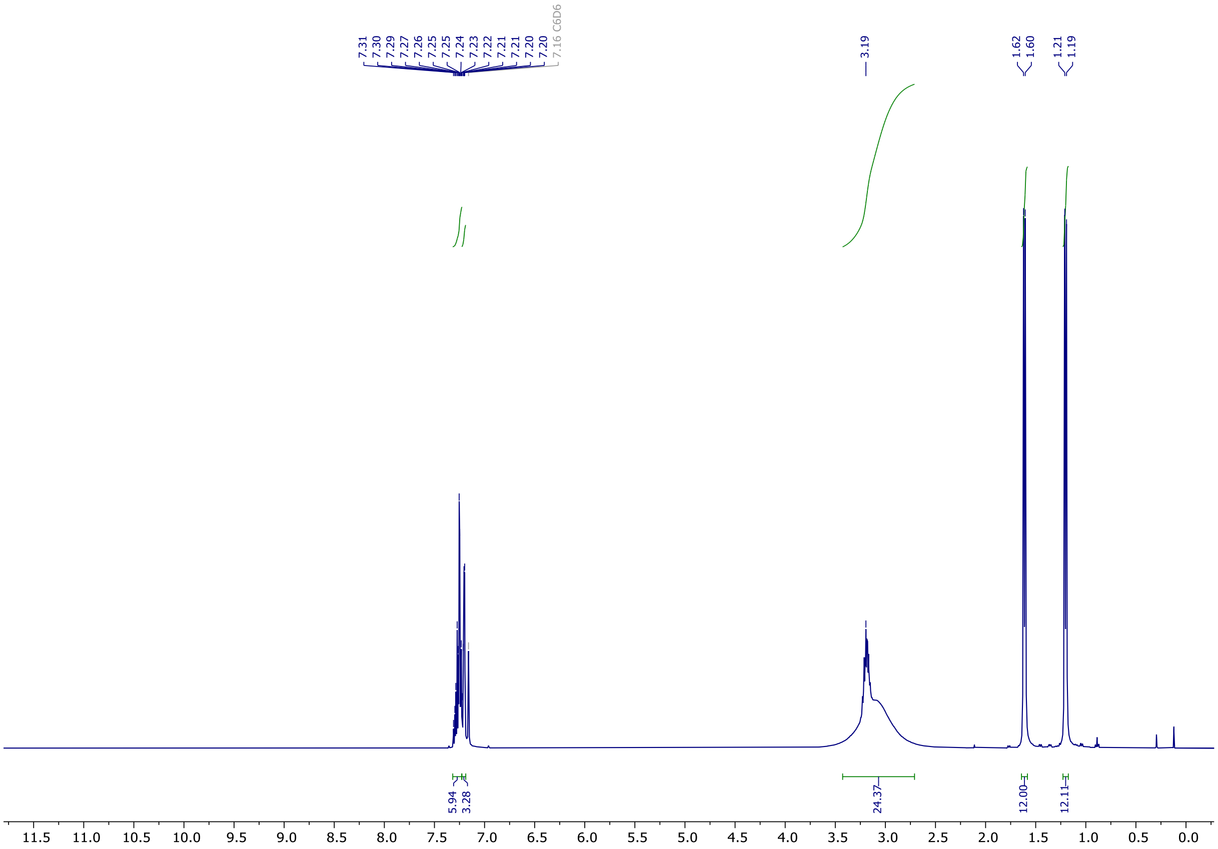

Figure S47: $^{13}C\{^1H\}$ NMR spectrum of **[^{Dipp}TerPO$_2$(CN)][Na(15-crown-5)]** (101 MHz, C_6D_6, rt).

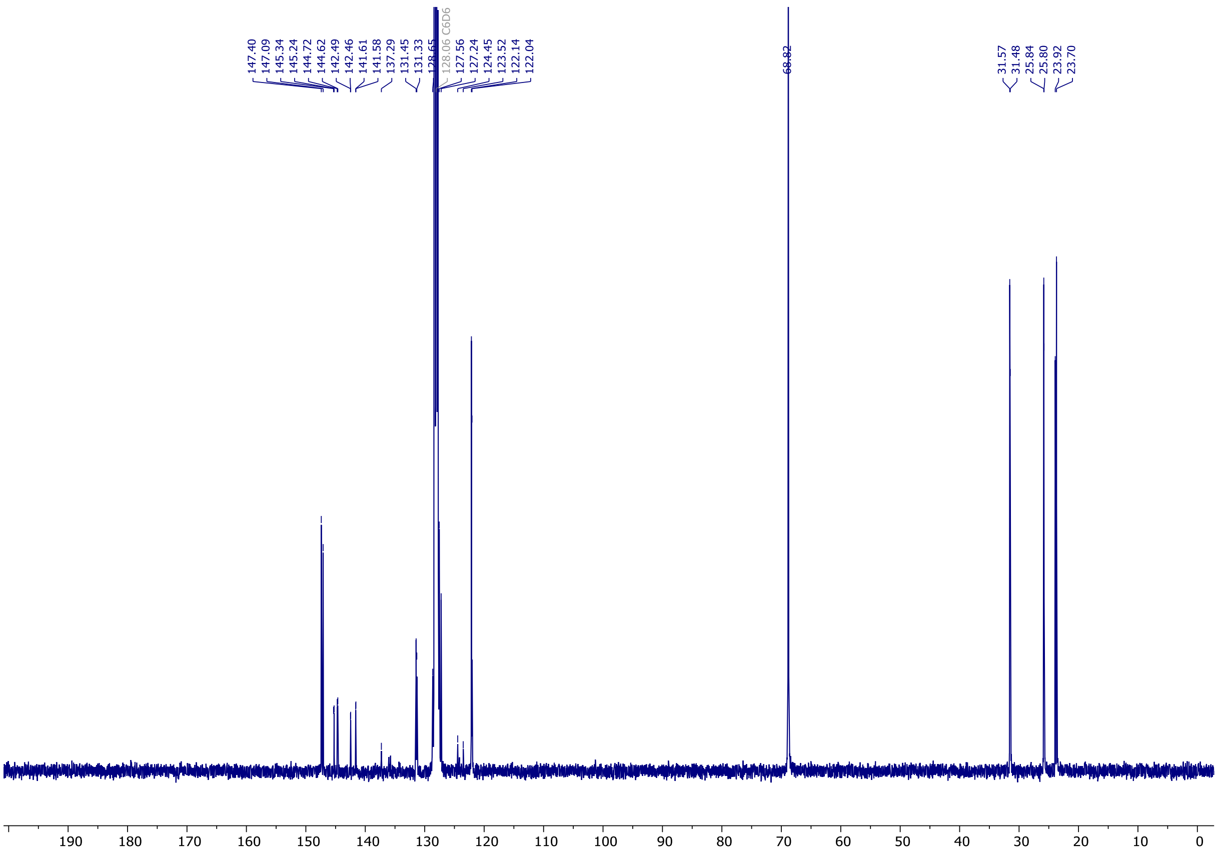

Figure S48: $^{31}P\{^1H\}$ NMR spectrum of **[^{Dipp}TerPO$_2$(CN)][Na(15-crown-5)]** (162 MHz, C_6D_6, rt).

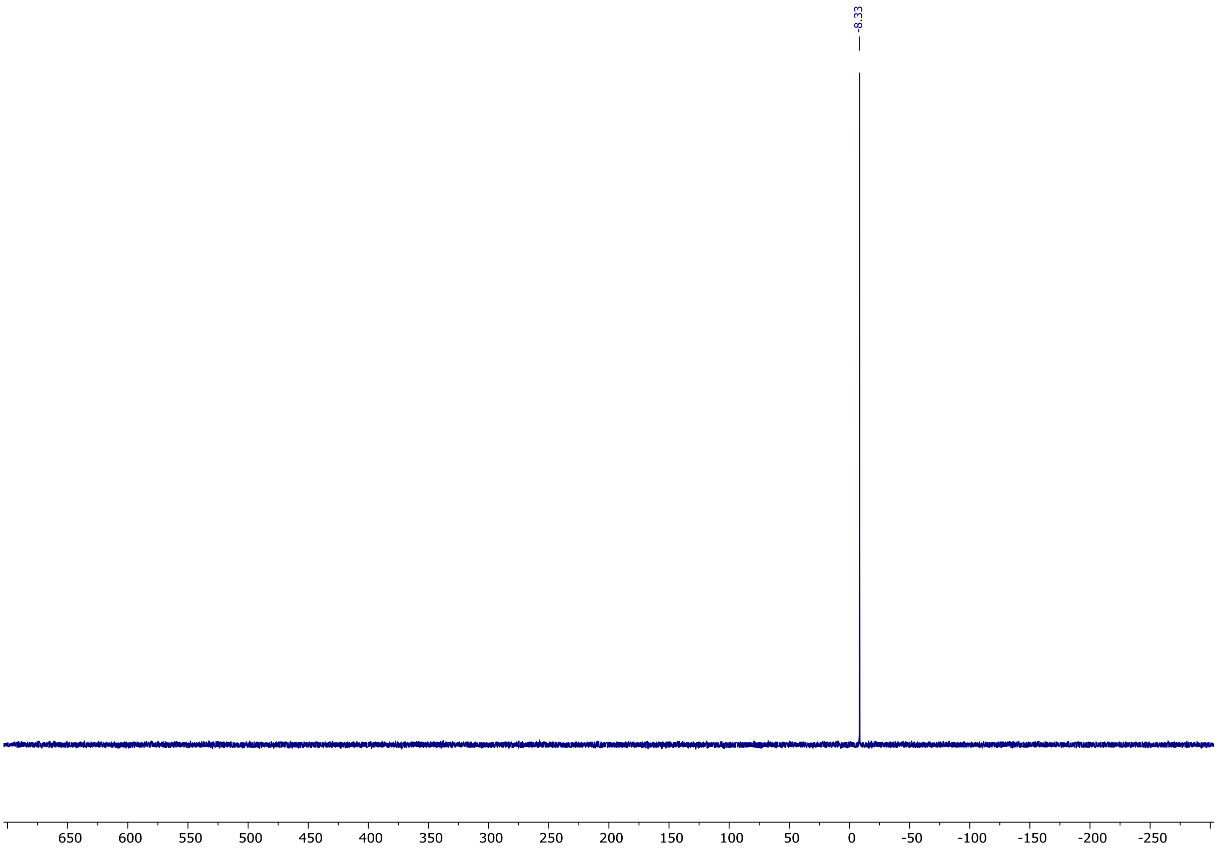

Figure S49: IR (ATR, solid sample, 32 scans, cm^{-1}) and Raman (633 nm, 20 s, 10 scans, cm^{-1}) spectrums of isolated crystals of **[^{Dipp}TerPO$_2$(CN)][Na(15-crown-5)]**.

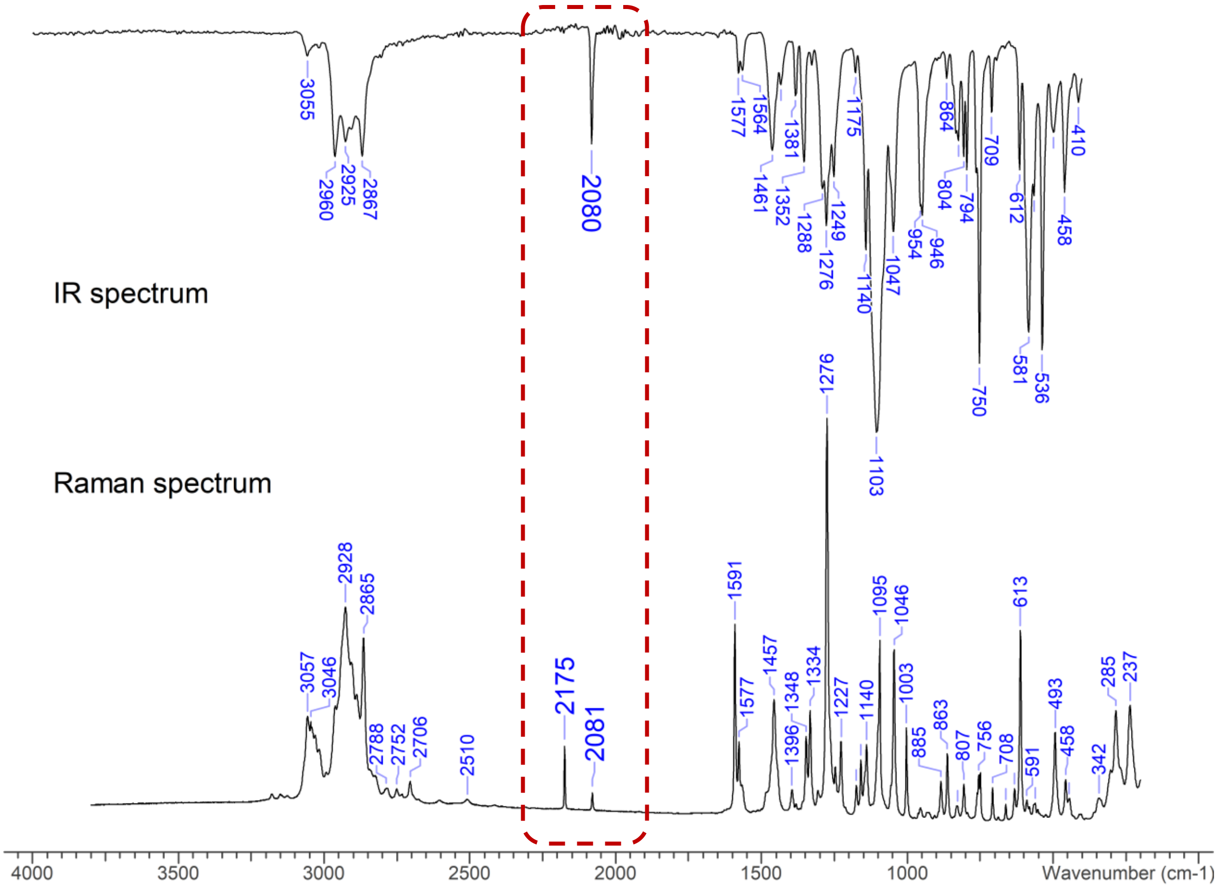

Figure S50: Variable Temperature experiment of $^{31}P\{^{1}H\}$ NMR spectrum of **[$^{Dipp}TerPO_2(CN)$][Na(15-crown-5)]** (162 MHz, toluene-d_8).

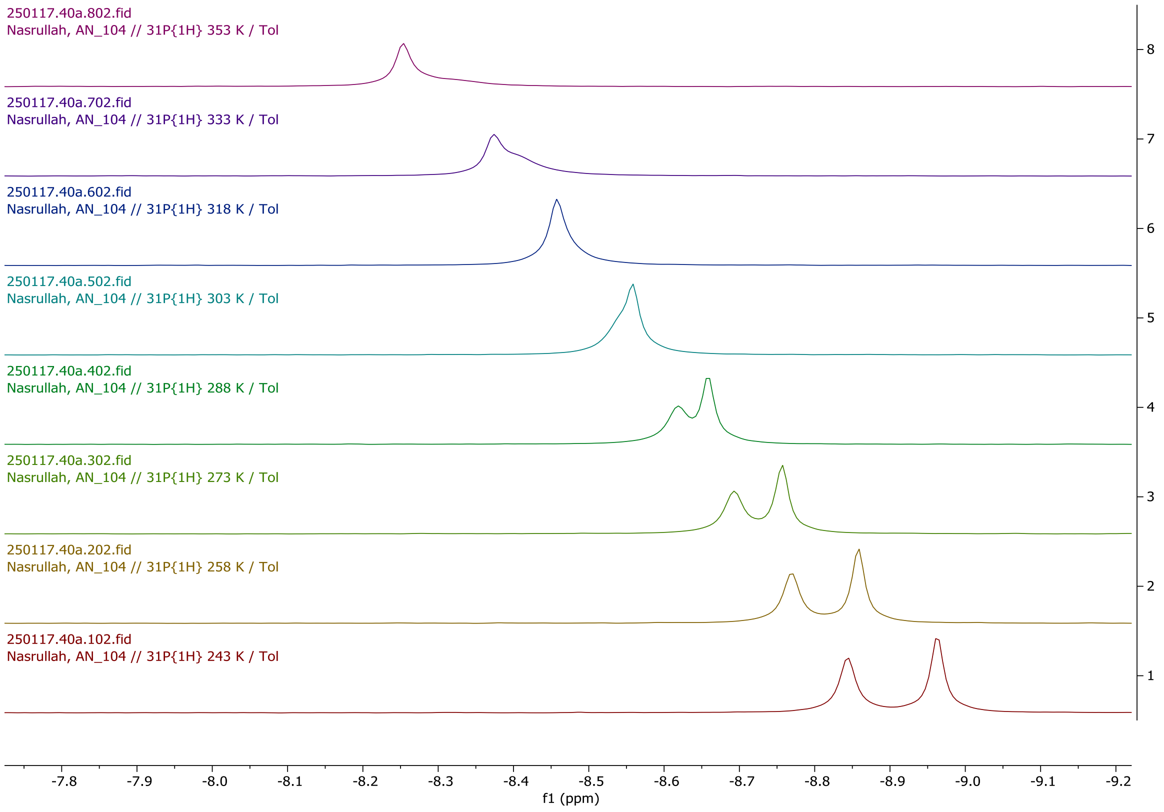

4.8 [^{Dipp}TerPO(CN)][K(18-crown-6)] (2·O)

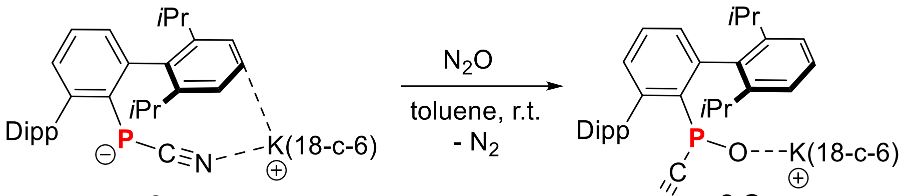

A) In a 10 mL Schlenk flask [^{Dipp}TerPCN][K(18-c-6)] salt (40 mg, 0.05 mmol) was suspended in toluene (2 mL). Then the reaction mixture was degassed via freeze-pump-thaw for 3 times. The evacuated flask was filled with 1 atm of dry N_2O (passed through Sicapent®) and the reaction mixture was stirred for 15 minutes at ambient temperature. The bright yellow suspension faded to pale-yellow and a gas evolution was observed. Then the reaction mixture was cooled down to −78 °C (EtOH/CO_2 cooling bath) and was evacuated (1×10^{-3} mbar) for 1 minute to remove the N_2 that was formed in the reaction. Afterwards the flask was refilled with N_2O gas (1 atm) and the procedure was repeated for 2 times. At this point no obvious colour change can be observed. The suspension remains as pale-yellow solution. Then all volatile components were removed *in vacuo* (1×10^{-3} mbar) and the remaining residue was washed with *n*-hexane (3 mL). The residue was redissolved in toluene (5 mL) and the clear filtrate was concentrated *in vacuo* (1×10^{-3} mbar) and stored at −30 °C, resulting in the deposition of pale-yellow crystals. The supernatant was transferred to another flask and was used to get further fractions of crystalline product. This route gave lower isolated yields compared to route **B**, which is described below.

B) [^{Dipp}TerPO_2(CN)][K(18-c-6)] can be synthesized from ^{Dipp}TerP(H)CN. In a 10 mL Schlenk flask ^{Dipp}TerP(H)CN (40 mg, 0.09 mmol), KH (6 mg, 0.15 mmol), and 18-crown-6 (24 mg, 0.09 mmol) were suspended in toluene (2 mL). The reaction mixture was stirred overnight for the in situ generation of [^{Dipp}TerPCN][K(18-c-6)] and afterwards was degassed via freeze-pump-thaw for 3 times. The evacuated flask was filled with 1 atm of N_2O (passed through Sicapent®) and the reaction mixture was stirred for 15 minutes at ambient temperature. Gas evolution and the colour changes were the

same as observed in route **A**. The work-up was the same according as described for route **A**, *vide supra*. The isolated crystals were dried *in vacuo* (1×10^{-3} mbar) for 2 hours in a water bath (50 °C), yielding colorless block-shaped crystals. Yield: 39 mg (0.05 mmol, 56%, (Route **B**)).

CHN calc. (found) in %: C 66.72 (66.68), H 7.94 (7.68), N 1.81 (1.50). **^{31}P{^{1}H} NMR** (C_6D_6, 121.5 MHz): δ = 81.09. **^{1}H NMR** (C_6D_6, 300.2 MHz): δ = 1.25 (d, $^{3}J(^{1}H,^{1}H)$ = 6.9 Hz, 6 H, CH(CH_3)$_2$), 1.27 (d, $^{3}J(^{1}H,^{1}H)$ = 6.9 Hz, 6 H, CH(CH_3)$_2$), 1.57 (d, $^{3}J(^{1}H,^{1}H)$ = 6.9 Hz, 6 H, CH(CH_3)$_2$), 1.65 (d, $^{3}J(^{1}H,^{1}H)$ = 6.9 Hz, 6 H, CH(CH_3)$_2$), 3.11 (s, 24 H, 18-c-6), 3.24 (sept, $^{3}J(^{1}H,^{1}H)$ = 6.9 Hz, 2 H, CH(CH$_3$)$_2$), 3.51 (sept, $^{3}J(^{1}H,^{1}H)$ = 6.9 Hz, 2 H, CH(CH$_3$)$_2$), 7.17-7.20 (m, 3 H, *m*-CH_{DipTer}, *p*-CH_{DipTer}), 7.21-7.24 (m, 4 H, *m*-CH_{Dip}), 7.25-7.32 (m, 2 H, *p*-CH_{Dip}). **^{13}C{^{1}H} NMR** (C_6D_6, 75.5 MHz): δ = 24.0 (s, 2C, CH(CH$_3$)$_2$), 26.0 (s, 1C, CH(CH$_3$)$_2$), 26.1 (s, 1C, CH(CH$_3$)$_2$), 31.3 (d, J_{CP} = 2.9 Hz, CH(CH$_3$)$_2$), 31.4 (s, CH(CH$_3$)$_2$), 69.9 (s, 18-c-6), 121.7 (s, CH$_{Aryl}$), 121.9 (s, CH$_{Aryl}$), 125.7 (s, CH$_{Aryl}$), 126.3 (s, CH$_{Aryl}$), 127.1 (s, CH$_{Aryl}$), 128.6 (s, CH$_{Aryl}$), 129.3 (s, CH$_{Aryl}$), 130.6 (s, CH$_{Aryl}$), 137.9 (s, $C_{q,Aryl}$), 141.7 (d, $J_{P,C}$ = 4.0 Hz, $C_{q,Aryl}$), 143.2 (d, $J_{P,C}$ = 17.5 Hz, $C_{q,Aryl}$), 146.7 (s, $C_{q,Aryl}$), 147.9 (d, $J_{P,C}$ = 1.9 Hz, $C_{q,Aryl}$), 148.1 (d, $^{1}J_{P,C}$ = 95.1 Hz, $C_{q,}$P). **IR** (ATR, 32 scans, cm^{-1}): $\tilde{\nu}$ = 3043 (w), 2960 (w), 2886 (w), 2865 (w), 2825 (w), 2745 (w), 1577 (w), 1560 (w), 1465 (w), 1430 (w), 1380 (w), 1351 (m), 1325 (w), 1283 (w), 1249 (w), 1177 (w), 1107 (s), 1057 (m), 961 (m), 838 (w), 820 (w), 804 (w), 792 (w), 759 (w), 748 (m), 699 (w), 589 (w), 542 (w), 479 (w), 445 (w). **MS** (ESI-TOF, pos., m/z, rel. int. > 10%): expected: m/z = 303.1205 [(18-crown-6)K]$^{+}$; found: m/z = 303.1206, (ESI-TOF, neg., m/z, rel. int. > 10%): expected: m/z = 470.2618 [^{Dip}TerPO(CN)]$^{-}$; found: m/z = 470.2620.

Single crystals of **2·O** suitable for X-ray diffraction can be grown from saturated toluene solution at −30 °C.

Figure S51: ^{1}H NMR spectrum of **[^{Dipp}TerPO(CN)][K(18-crown-6)]** (300.2 MHz, C_6D_6, rt).

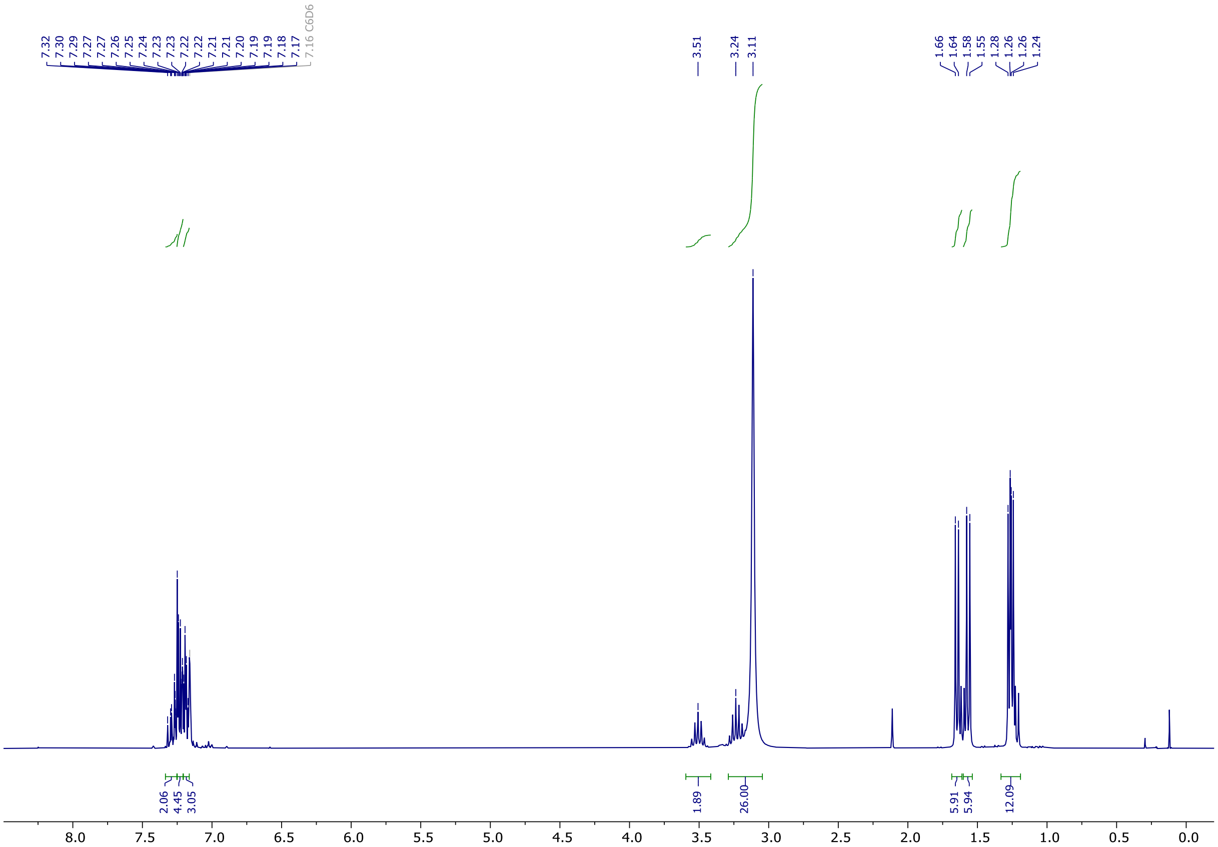

Figure S52: $^{13}C\{^1H\}$ NMR spectrum of **[^{Dipp}TerPO(CN)][K(18-crown-6)]** (75.5 MHz, C_6D_6, rt).

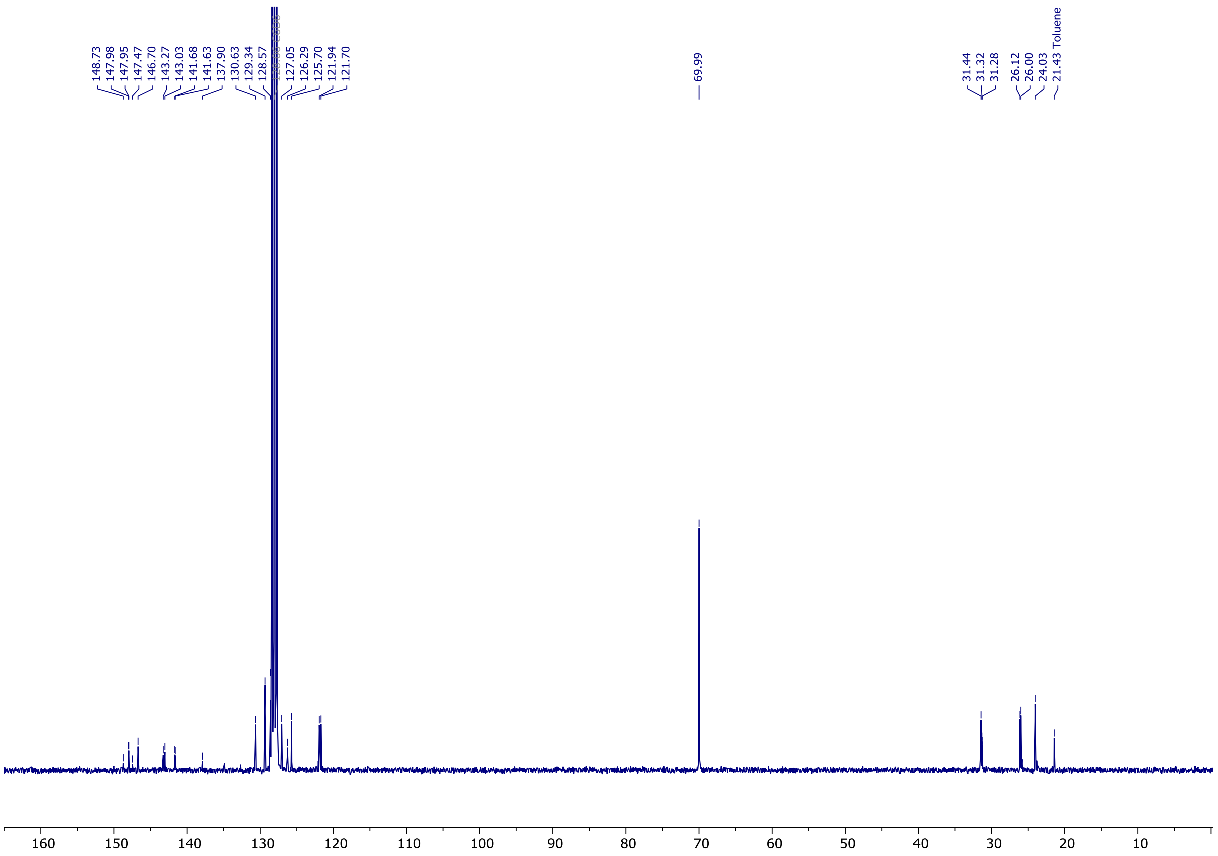

Figure S53: $^{31}P\{^1H\}$ NMR spectrum of **[^{Dipp}TerPO(CN)][K(18-crown-6)]** (121.5 MHz, C_6D_6, rt).

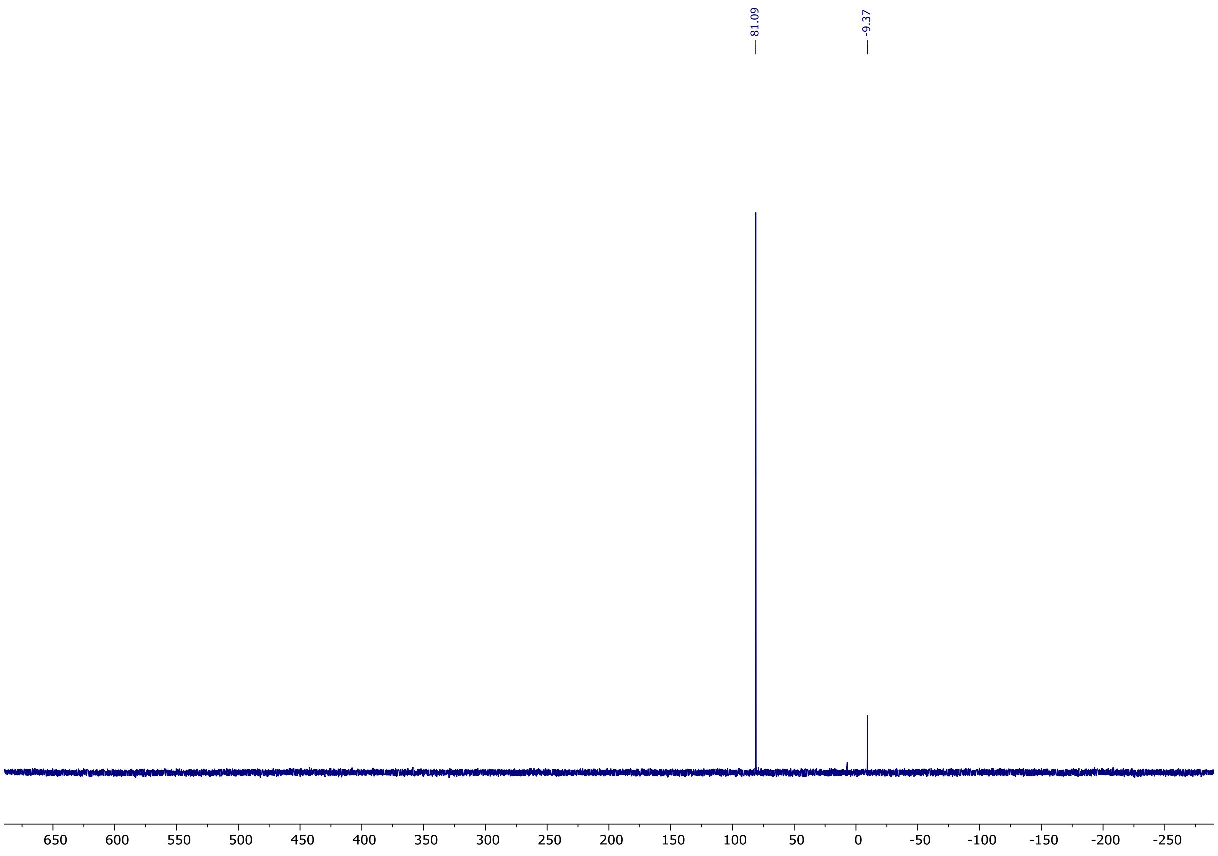

Figure S54: IR spectrum of **[DipTerPO(CN)][K(18-crown-6)]** (ATR, solid sample, 32 scans, cm^{-1}).

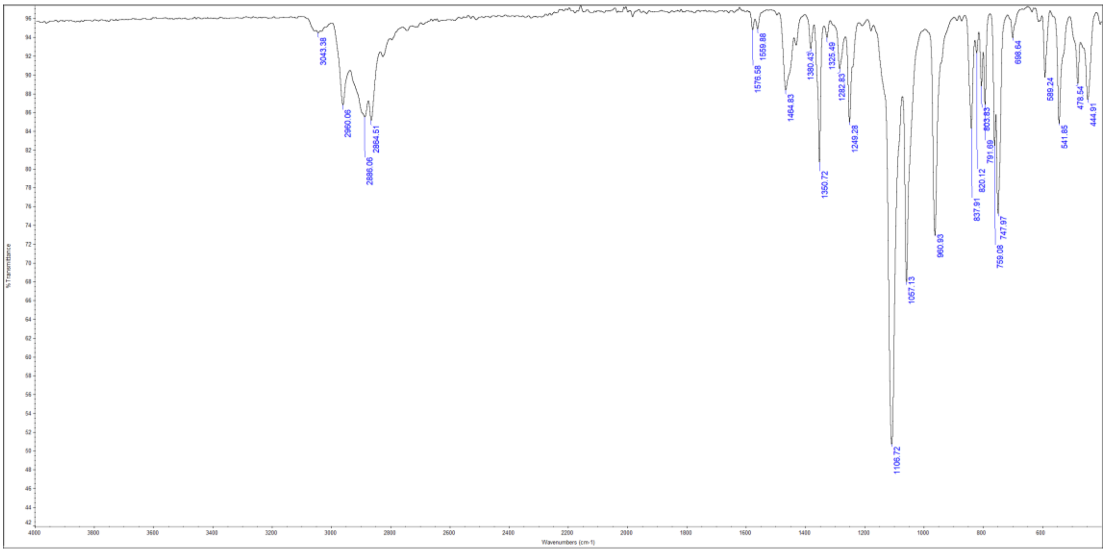

4.9 [^{Dip}TerPO(CN)][Na(15-crown-5)] (4·O)

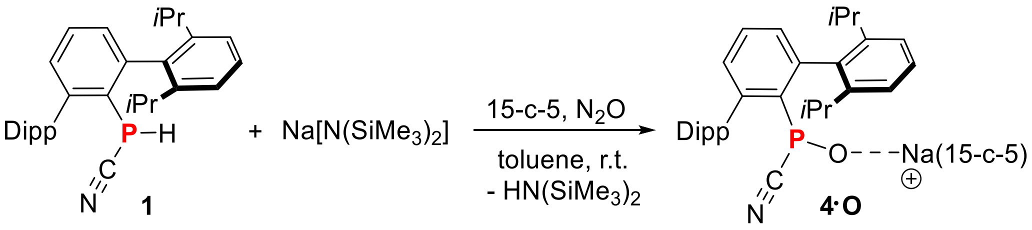

In a 10 mL Schlenk flask 15-crown-5 (20 mg, 0.09 mmol), ^{Dipp}TerP(H)CN (41 mg, 0.09 mmol) and NaHMDS (17 mg, 0.09 mmol) were dissolved in toluene (2 mL). The reaction mixture was stirred overnight at ambient temperature. Then the reaction mixture was degassed via freeze-pump-thaw for 3 times. The evacuated flask was filled with 1 atm of dry N_2O (passed through Sicapent®) and the reaction mixture was stirred for 15 minutes at ambient temperature. The bright yellow suspension faded to pale-yellow solution and gas evolution was observed. Then the reaction mixture was cooled down to −78 °C (EtOH/CO_2 cooling bath) and was evacuated (1×10^{-3} mbar) for 1 minute to remove the N_2 that was formed in the reaction. Afterwards the flask was refilled with N_2O gas (1 atm) and the procedure was repeated for 2 times. At this point no obvious colour change can be observed. The suspension remains as pale-yellow solution. Then all volatile components were removed *in vacuo* (1×10^{-3} mbar) and the remaining residue was washed with *n*-hexane (3 mL). The residue was redissolved in toluene (5 mL) and the clear filtrate was concentrated *in vacuo* (1×10^{-3} mbar) and stored at −30 °C for crystallization, resulting in the deposition of a pale-yellow powder. Then all volatile components were removed *in vacuo* (1×10^{-3} mbar), yielding pale yellow powder. Yield: 35 mg (0.054 mmol, 61%).

CHN calc. (found) in %: C 68.98 (66.08), H 8.05 (7.34), N 1.96 (1.69) were measured with oxidizing agent, vanadium pentoxide (V_2O_5). Deviations most likely result from incomplete combustion. **^{31}P{^{1}H} NMR** (C_6D_6, 121.5 MHz): δ = 82.9. **^{1}H NMR** (C_6D_6, 300.1 MHz): δ = 1.25 (d, $^3J(^1H,^1H)$ = 6.9 Hz, 6 H, CH(C*H*$_3$)$_2$), 1.26 (d, $^3J(^1H,^1H)$ = 6.9 Hz, 6 H, CH(C*H*$_3$)$_2$), 1.57 (d, $^3J(^1H,^1H)$ = 6.9 Hz, 6 H, CH(C*H*$_3$)$_2$), 1.66 (d, $^3J(^1H,^1H)$ = 6.9 Hz, 6 H, CH(C*H*$_3$)$_2$), 2.87 (s, 20 H, 15-c-5)*, 3.22 (sept, $^3J(^1H,^1H)$ = 6.9 Hz, 2 H, C*H*(CH$_3$)$_2$),

3.52 (sept, $^{3}J(^{1}H,^{1}H)$ = 6.9 Hz, 2 H, $CH(CH_3)_2$), 7.17-7.20 (m, 3 H, *m*-CH_{DipTer}, *p*-CH_{DipTer}), 7.21-7.30 (m, 6 H, *m*-CH_{Dip}, *p*-CH_{Dip}). **^{13}C{^{1}H} NMR** (C_6D_6, 75.5 MHz): δ = 23.91 (d, $J_{P,C}$ = 5.5 Hz, $CH(CH_3)_2$), 25.9 (s, $CH(CH_3)_2$), 26.1 (s, $CH(CH_3)_2$), 31.4 (d, $J_{P,C}$ = 7.2 Hz, $CH(CH_3)_2$), 68.8 (s, 15-c-5), 121.8 (s, CH_{Aryl}), 122.1 (s, CH_{Aryl}), 122.9 (d, $^{1}J_{P,C}$ = 131 Hz, PC≡N), 126.4 (s, CH_{Aryl}), 127.1 (s, CH_{Aryl}), 130.7 (s, $C_{q,Aryl}$), 141.5 (d, $J_{P,C}$ = 3.9 Hz, $C_{q,Aryl}$), 142.9 (s, $C_{q,Aryl}$), 143.2 (s, $C_{q,Aryl}$), 146.7 (d, $^{1}J_{P,C}$ = 87.5 Hz, C_{q},P). **IR** (ATR, 32 scans, cm^{-1}): $\tilde{\nu}$ = 3051 (w), 2955 (m), 2919 (w), 2866 (w), 1576 (w), 1558 (w), 1460 (w), 1430 (w), 1380 (w), 1352 (m), 1326 (w), 1292 (w), 1280 (w), 1250 (w), 1176 (w), 1116 (s), 1103 (s), 1061 (s), 947 (m), 862 (w), 831 (w), 822 (w), 804 (w), 792 (w), 753 (s), 691 (w), 587 (w), 544 (m), 524 (w), 482 (w), 450 (w), 439 (w). **MS** (ESI-TOF, pos., m/z, rel. int. > 10%): expected: m/z = 243.1203 [(15-crown-5)Na]$^{+}$; found: m/z = 243.1206, (ESI-TOF, neg., m/z, rel. int. > 10%): expected: m/z = 470.2618 [^{Dip}TerPO(CN)]$^{-}$; found: m/z = 470.2620.

Figure S55: ^{1}H NMR spectrum of **[^{Dip}TerPO(CN)][Na(15-crown-5)]** (300 MHz, C_6D_6, rt).

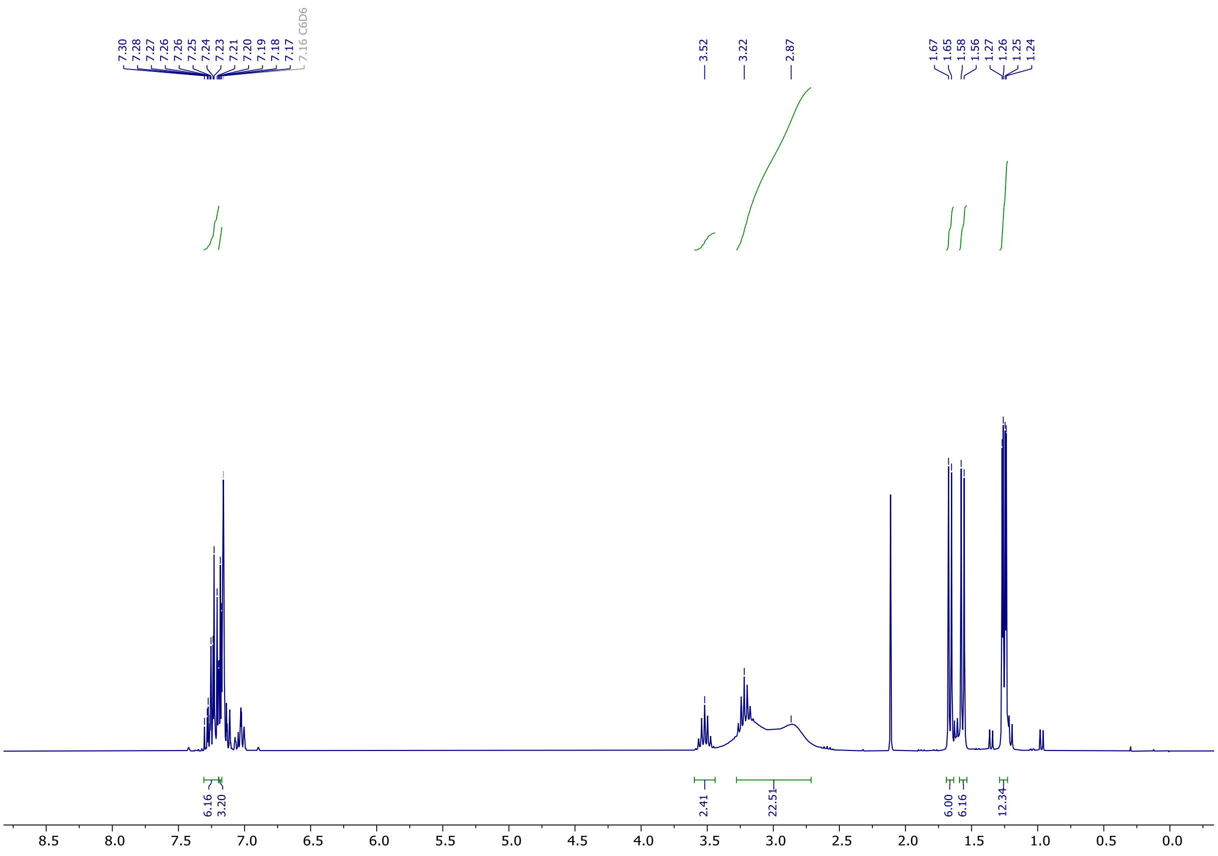

Figure S56: $^{13}C\{^1H\}$ NMR spectrum of **[^{Dip}TerPO$_2$(CN)][Na(15-crown-5)]** (75.5 MHz, C_6D_6, rt).

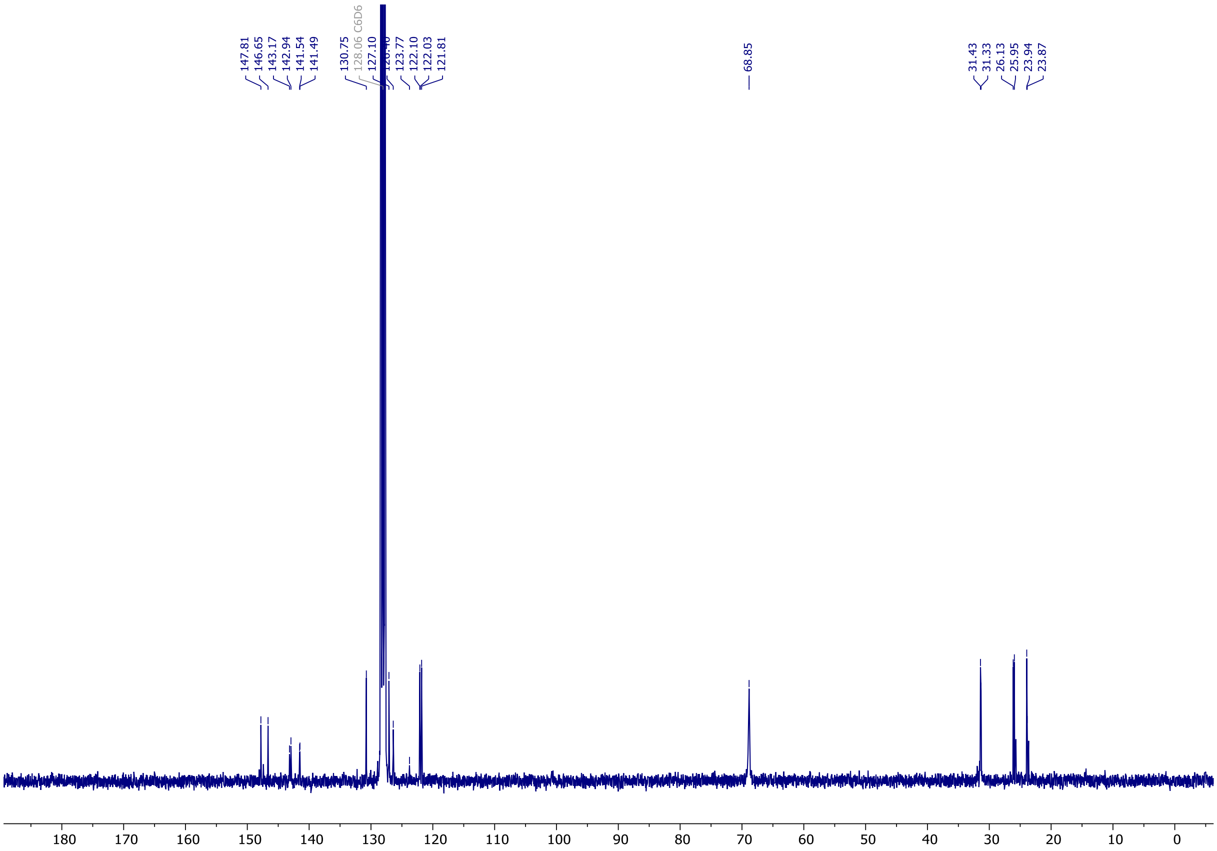

Figure S57: $^{31}P\{^1H\}$ NMR spectrum of **[^{Dip}TerPO$_2$(CN)][K(15-crown-5)]** (121.5 MHz, C_6D_6, rt).

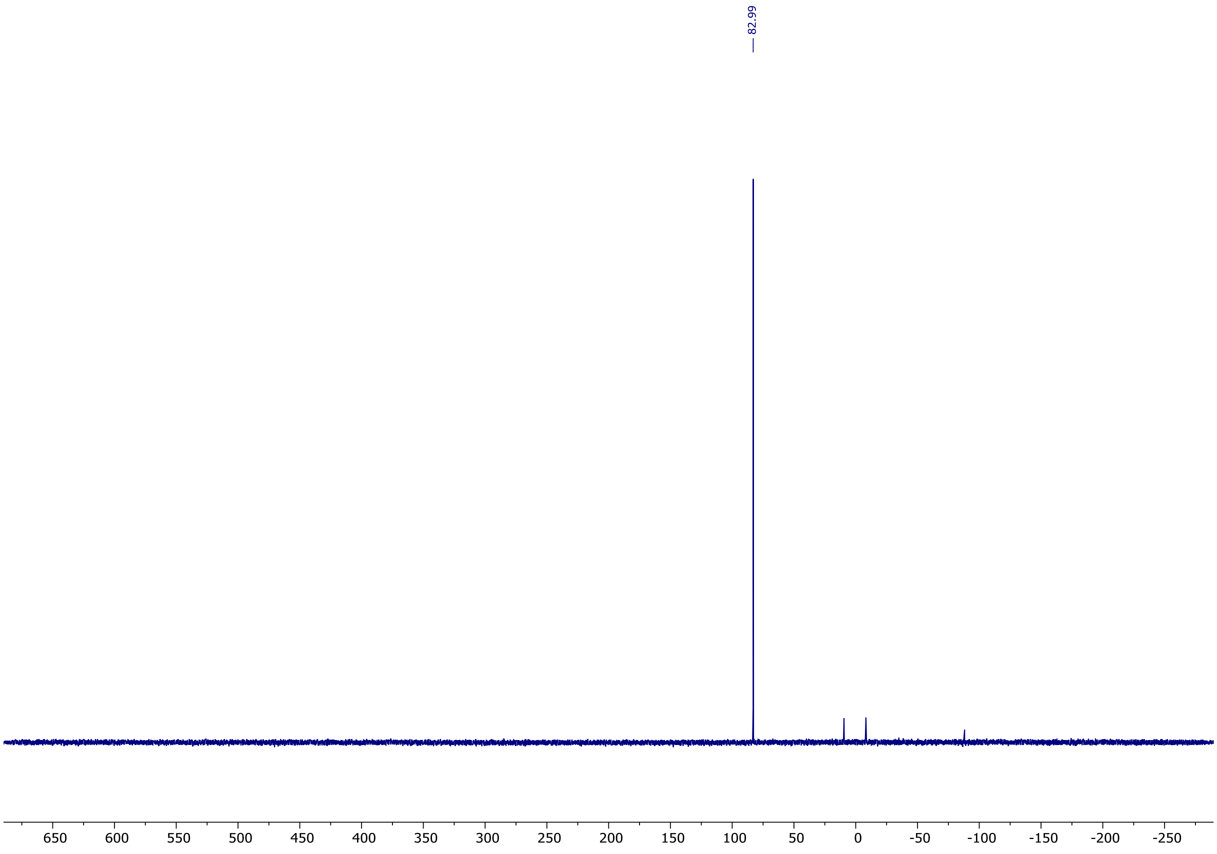

Figure S58: IR spectrum of **[^{Dip}TerPO$_2$(CN)][K(15-crown-5)]** (ATR, solid sample, 32 scans, cm^{-1}).

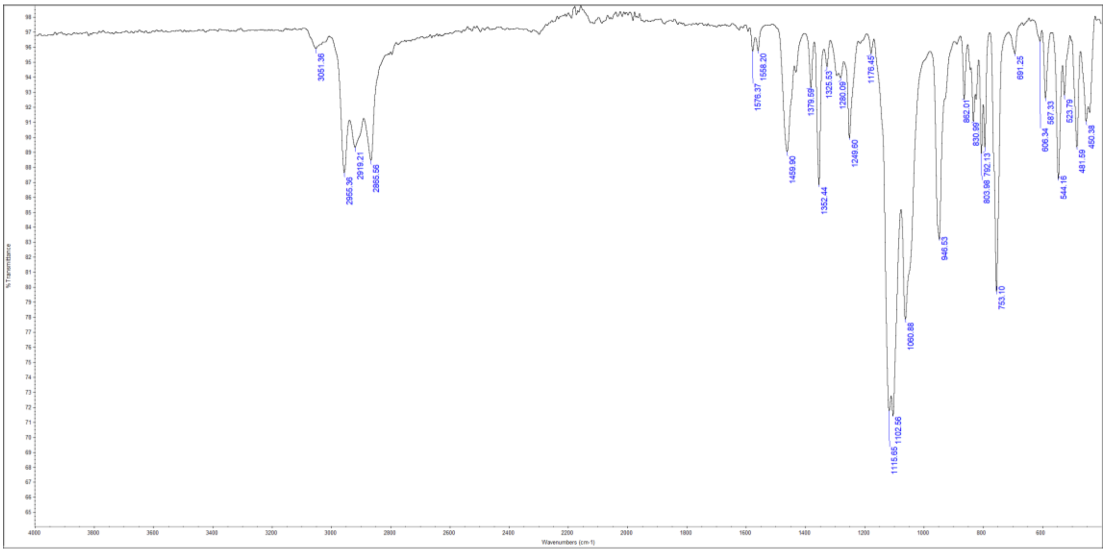

5 Computational details

5.1 General remarks

Computations were carried out using Gaussian09[8], Gaussian16[9] or ORCA 4.2.1[10] and the standalone version of NBO 6.0.[11]

Structure optimizations employed the hybrid DFT functional PBE0[12] in conjunction with Grimme's dispersion correction D3(BJ)[13] and the def2-SVP basis set[14] (notation PBE0-D3/def2-SVP) for the full structures (**2·O$_2$**, **4·O$_2$**, **2·O** and **2·S$_2$**). For thermodynamic and mechanistic considerations the truncated models [PhPO$_2$(CN/NC)]K, [PhPO$_2$(CN/NC)]Na, [PhPS$_2$(CN/NC)]K and [PhPO(CN)]$^-$ were chosen and initial optimizations and frequency analyses were carried out at the PBE0-D3/def2-TZVP level of theory. All structures were fully optimized and confirmed as minima by frequency analyses. Calculated frequencies were scaled by 0.950 (as derived from Truhlar's Reduced Scale Factor Optimization model).[15] Partial charges were determined by Natural Population analysis using NBO 6.0. Chemical shifts were derived by the GIAO method at the PBE0-D3/def2-TZVP//PBE0-D3/def2-SVP level of theory.[16] The calculated absolute shifts ($\sigma_{calc,X}$) were referenced to the experimental absolute shift of 85% H_3PO_4 in the gas phase ($\sigma_{ref,1}$ = 328.35 ppm),[17] using PH_3 ($\sigma_{ref,2}$ = 594.45 ppm) as a secondary standard:[18]

$$\begin{aligned}\delta_{calc,X} &= (\sigma_{ref,1} - \sigma_{ref,2}) - (\sigma_{calc,X} - \sigma_{calc,PH_3}) \\ &= \sigma_{calc,PH_3} - \sigma_{calc,X} - 266.1\ \text{ppm}\end{aligned}$$

At the PBE0-D3/def2-TZVP level of theory, σ_{calc,PH_3} amounts to +567.77 ppm.

Please note that all computations were carried out for single, isolated molecules in the gas phase (ideal gas approximation). There may well be significant differences between gas phase and condensed phase.

5.2 Summary of calculated data

Table S5. Summary of calculated data.

Compd.	PG	N_{imag}	E_{tot}[a]	U_0[a]	U_{298}[a]	H_{298}[a]	G_{298}[a]
**2**[b]	C_1	0	−3118.3034	−3117.3283	−3117.2711	−3117.2701	−3117.4243
**4**[b]	C_1	0	−2527.1911	−2526.2772	−2526.2241	−2526.2232	−2526.3669
**2·O$_2$_CN**[b]	C_1	0	−3268.5765	−3267.5917	−3267.5330	−3267.5321	−3267.6857
**2·O$_2$_NC**[b]	C_1	0	−3268.5649	−3267.5806	−3267.5218	−3267.5208	−3267.6751
**2·S$_2$_CN**[b]	C_1	0	−3914.2413	−3913.2610	−3913.2012	−3913.2002	−3913.3576
**2·S$_2$_NC**[b]	C_1	0	−3914.2226	−3913.2428	−3913.1830	−3913.1820	−3913.3395
**4·O$_2$_CN**[b]	C_1	0	−2677.4664	−2676.5428	−2676.4881	−2676.4872	−2676.6325
**4·O$_2$_NC**[b]	C_1	0	−2677.4536	−2676.5305	−2676.4757	−2676.4747	−2676.6205
**2·O**[b]	C_1	0	−3193.4273	−3192.4478	−3192.3899	−3192.3890	−3192.5406
[PhPO$_2$(CN)]K[c]	C_1	0	−1415.7454	−1415.6357	−1415.6240	−1415.6231	−1415.6760
[PhPO$_2$(CN)]K_TS[c]	C_1	1	−1415.6939	−1415.5866	−1415.5747	−1415.5737	−1415.6268
[PhPO$_2$(NC)]K[c]	C_1	0	−1415.7351	−1415.6259	−1415.6141	−1415.6131	−1415.6662
[PhPO$_2$(CN)]Na[c]	C_1	0	−978.1740	−978.0639	−978.0525	−978.0515	−978.1031
[PhPO$_2$(CN)]Na_TS [c]	C_1	1	−1415.7351	−978.0138	−978.0020	−978.0011	−978.0533
[PhPO$_2$(NC)]Na[c]	C_1	0	−978.1215	−978.0542	−978.0426	−978.0417	−978.0935
[PhPO(CN)]$^-$ [c]	C_1	0	−740.7279	−740.6256	−740.6165	−740.6155	−740.6609

[a] energy in a.u.; [b] PBE0-D3/def2-SVP; [c] PBE0-D3/def2-TZVP

5.3 Calculated NMR and IR/RAMAN data

NMR data were calculated at the PBE0-D3/def2-TZVP level of theory, using the structures optimized at the PBE-D3/def2-SVP or PBE0-D3/def2-TZVP level of theory.

Table S6. Calculated NMR shifts at PBE0-D3/def2-TZVP. Experimental values given in brackets.

Compound	Nucleus	Group	Mult.	δ [ppm]
[^{Dipp}TerPCN][K(18-c-6)] (**2**)	^{31}P	*P*CN	s	−138.9 (−127.2)
[^{Dipp}TerPCN][Na(15-c-5)] (**4**)	^{31}P	*P*CN	s	−137.6 (−129.9)
[^{Dipp}TerPO$_2$(CN)][K(18-c-6)] (**2·O$_2$**)	^{31}P	*P*O$_2$(CN)	s	−4.8 (−9.3)
[^{Dipp}TerPO$_2$(NC)][K(18-c-6)] (**2·O$_2$**)	^{31}P	*P*O$_2$(NC)	s	−0.8 (−9.0)
[^{Dipp}TerPS$_2$(CN)][K(18-c-6)] (**2·S$_2$**)	^{31}P	*P*S$_2$(CN)	s	47.0 (32.3)
[^{Dipp}TerPS$_2$(NC)][K(18-c-6)] (**2·S$_2$**)	^{31}P	*P*S$_2$(NC)	s	83.6 (n.o.)
[^{Dipp}TerPO$_2$(CN)][Na(15-c-5)] (**4·O$_2$**)	^{31}P	*P*O$_2$(CN)	s	0.6 (−8.3)
[^{Dipp}TerPO$_2$(NC)][Na(15-c-6)] (**4·O$_2$**)	^{31}P	*P*O$_2$(NC)	s	2.5 (−8.3)
[^{Dipp}TerPO(CN)][K(18-c-6)] (**2·O**)	^{31}P	*P*O(CN)	s	72.8 (87.9)
[PhPO$_2$(CN)K]	^{31}P	*P*O(CN)	s	−1.5
[PhPO$_2$(NC)K]	^{31}P	*P*O(NC)	s	−0.8
[PhPO$_2$(CN)Na]	^{31}P	*P*O(CN)	s	4.3
[PhPO$_2$(NC)Na]	^{31}P	*P*O(NC)	s	4.3

Table S7. Calculated values for the PCN and PNC stretches $\tilde{\upsilon}$ in [cm^{-1}]. Intensities given in brackets. Calculated frequencies were scaled by 0.950 (as derived from Truhlar's Reduced Scale Factor Optimization model).

Compound	$\tilde{\upsilon}$ (PCN; IR)	$\tilde{\upsilon}$ (PNC; IR)	$\tilde{\upsilon}$ (PCN; RAMAN)	$\tilde{\upsilon}$ (PNC; RAMAN)
[^{Dipp}TerPO$_2$(CN)][K(18-c-6)] (**2·O$_2$**)	2216 (0.3)		2216 (54)	
[^{Dipp}TerPO$_2$(NC)][K(18-c-6)] (**2·O$_2$**)		2102 (148)		2102 (53)
[^{Dipp}TerPS$_2$(CN)][K(18-c-6)] (**2·S$_2$**)	2191 (0.06)		2191 (112)	
[^{Dipp}TerPS$_2$(NC)][K(18-c-6)] (**2·S$_2$**)		2067 (190)		2067 (95)
[^{Dipp}TerPO$_2$(CN)][Na(15-c-5)] (**4·O$_2$**)	2217 (0.1)		2217 (74)	
[^{Dipp}TerPO$_2$(NC)][Na(15-c-6)] (**4·O$_2$**)		2103 (174)		2103 (64)
[^{Dipp}TerPO(CN)][K(18-c-6)] (**2·O**)	2153 (17)		-	-

5.4 Reaction path analysis

Calculations were carried out using the truncated models [PhPO$_2$(CN/NC)]M (M = Na, K), to eliminate potential conformer changes within the bulky ^{Dipp}Ter-substituent and to focus on the PCN-unit. More accurate estimates of the electronic energy were obtained at the DLPNO-CCSD(T)/def2-TZVP[19] using the thermal corrections obtained at the PBE0-D3/def2-TZVP level of theory. For the truncated model solvent effects were not considered. The reaction pathway was elucidated using the Nudged Elastic Band (NEB)[20] method. The validity of the path was checked using IRC calculations in forward and reverse reaction, allowing to determine whether starting materials, transition states and products are connected. The following processes were considered:

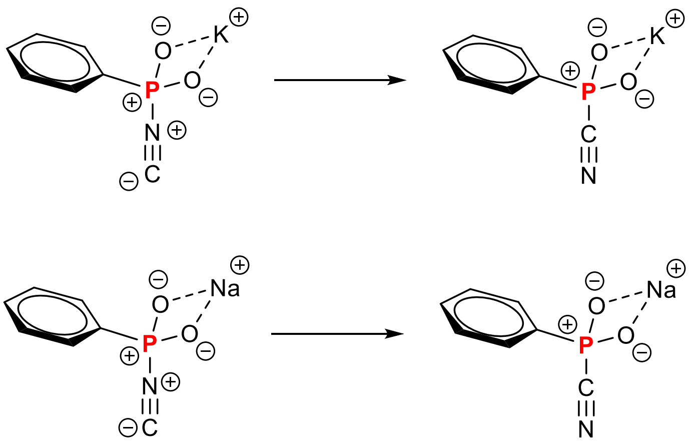

Table S8. Absolute energies (in a.u.) based on coupled cluster methods, E_{CC} (DLPNO-CCSD(T)/def2-TZVP) and and correction to Gibbs free energy (Cor.) at the PBE0-D3/def2-TZVP level of theory.

Compd.	E_{CC}	*Cor.*
[PhPO$_2$(CN)]K	−1415.5634	0.0695
[PhPO$_2$(CN)]K_TS	−1414.5125	0.0672
[PhPO$_2$(NC)]K	−1414.5527	0.0689
[PhPO$_2$(CN)]Na	−9772548	0.0709
[PhPO$_2$(CN)]Na_TS	−977.2025	0.0681
[PhPO$_2$(NC)]Na	−977.2441	0.0704

The results clearly show that the $[PhPO_2(NC)]M$ to $[PhPO_2(CN)]M$ isomerization is a thermodynamically driven process, with the PCN isomer being more stable by −26.5 kJ·mol^{-1} (M = Na) or −26.6 kJ·mol^{-1} (M = K), respectively. The transition states (TS) found for both counter cations show the same orientation of the CN^- in the transition state (TS), with the CN^- bridging between the P atom and M. In the TS C is oriented towards the P atom while N is oriented towards M. This is accompanied by a significant planarization of the $PhPO_2$ unit, in line with the description as CN^- complex of a dioxophosphorane. Both TS are only minimally different in energy $\Delta G^{\ddagger}$ (M=Na)= 103.4 kJ·mol^{-1} and $\Delta G^{\ddagger}$ (M=K)= 100.9 kJ·mol^{-1}. Even though a truncated model is used, both paths are in line with a facile isomerization at 80 °C in benzene solution, as experimentally verified.

Figure S59. Computed thermal reaction pathway for the NC to CN isomerism in $[PhPO_2(NC)]M$ (M = Na, K) (DNLPO-CCSD(T)-/def2TZVP//PBE0-D3/def2-TZVP, $c°$ = 1 mol·L^{-1}).

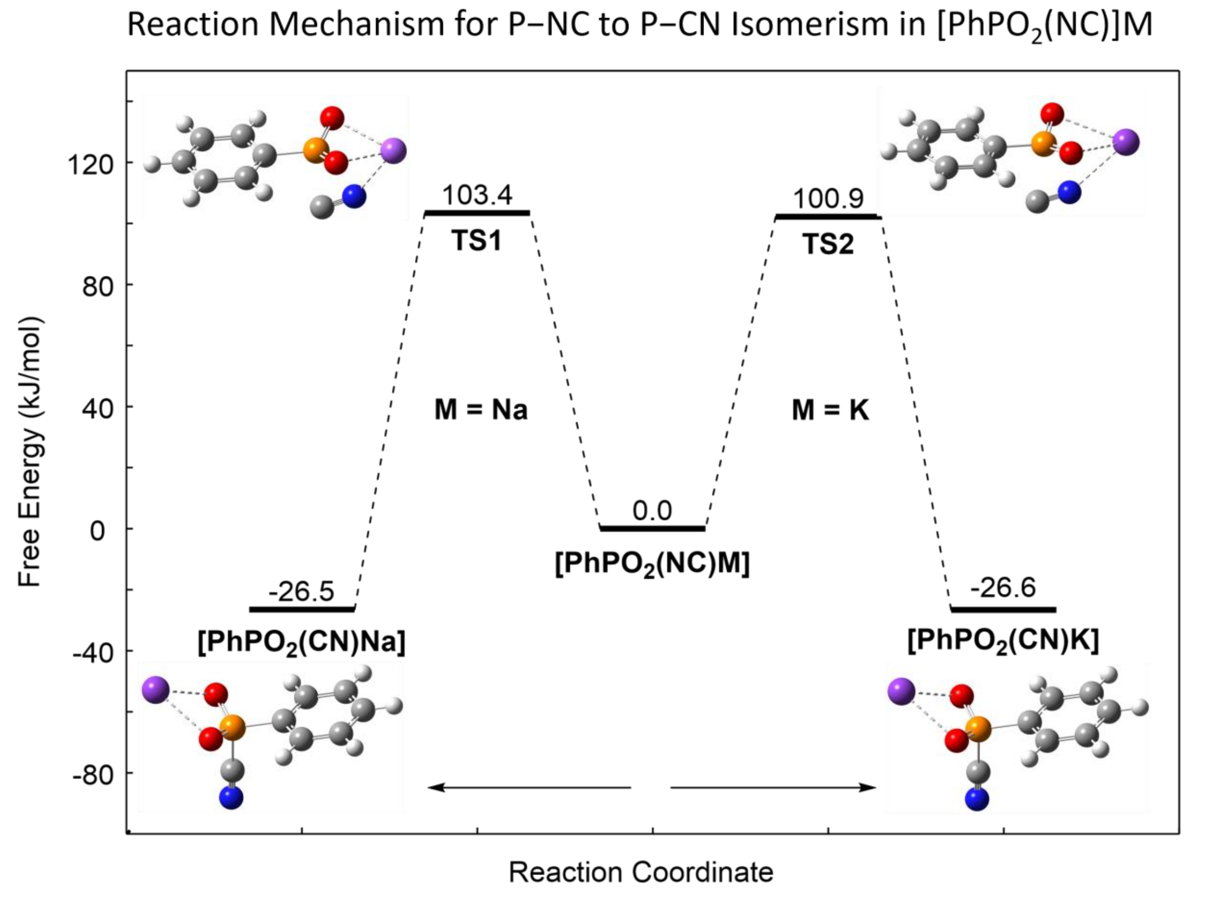

5.5 NBO analyses

NBO analyses were carried out for the CN and NC isomers of the isolated $[PhPO_2(NC/CN)]^-$ anions at the PBE0-D3/def2-TZVP level of theory. The truncated model was chosen to allow Natural Resonance Theory (NRT) calculations, which cannot be performed for the full systems. Inspection of the of the natural charges derived from Natural Population Analysis (NPA) revealed significantly electron-deficient P atoms in both isomers (+2.11 PCN; +2.27 PNC), while significant negative charge is transferred from the $[CN]^-$ to the $PhPO_2$ unit (CT PCN: −0.51 e; PNC: −0.37 e) (Table S7). In both forms the oxygen atoms carry a negative partial charge. NRT analyses show that the leading resonance structures in both isomers is one with a tetravalent phosphorus atom with a covalently attached CN (41.8%) or NC (28.8%) unit, respectively. The Lewis structure with the second highest weight in case of the PCN isomer is an ionic form with no bond between phosphorus and one of the O atoms (12.2%). The phosphorus atom is still tetravalent with one P−O double bond. A third form shows no bond between the P and C_{CN} atom with a tetravalent P atom and a delocalized P=O bond (10.4%). This is also reflected in the Wiberg Bond Indexes (WBI) of the P−O bonds of 1.15. Conversely, the P−C_{CN} bond has a rather low WBI of 0.72, indicative of a polarized (P: 31.0 %; C: 69.0 %) single bond with partial ionic character. In the NC-isomer the P−N_{CN} has a higher ionic character, which is reflected in a lower WBI (0.58) and a stronger polarization of the bond (P: 21.6 %; N: 78.4 %). Moreover, for the PNC isomer the leading covalent resonance structure in the NRT analysis has a lower weight of 28.8 %, while the form with a non-bonding CN unit has a considerably higher weight of 27.8%. Figure S39 ($[PhPO_2(CN)]^-$) and S40 ($PhPO_2(NC)]^-$) summarizes the results from NRT analyses, while Table SX summarizes the NPA charges and WBIs.

To investigate the bonding in phosphinidene monoxide species **2·O** and **4·O** the truncated isolated anion $[PhPO(CN)]^-$ was chosen. As there is no experimental evidence for a CN/NC isomerism in these species only the PCN was investigated. The P atom $[PhPO(CN)]^-$ carries a significant positive charge (1.10 e), while the C_{CN} (−0.06 e) and

N_{CN} (−0.45 e) carry negative natural charges (Table S7). This can be translated to an effective charge transfer −0.49 e to the PhPO unit. The P−C_{CN} bond has a higher WBI (0.82) compared to $[PhPO_2(CN)]^-$, indicating a higher covalent character of the polarized bond (P: 31%, C: 69%). Moreover, the P atom has a lone pair of electrons with high s-character (s: 55.5%; p: 44.5%), which is delocalized (according to 2nd order perturbation theory) into the C−N σ*- (8.38 kcal/mol) and π*-orbitals (8.49 kcal/mol), respectively, resulting in a decrease in the WBI of the C≡N bond (2.86). These findings are in line with the molecular structure of **2·O**, with a rather long P−C bond, and a P−O bond that is significantly longer than in a recently reported free phosphinidene monoxide. NRT analyses show mainly covalent character of the P−CN bond (52.0%), while the minor resonance (7.5%) form represents the ionic character of the bond with a P=O double bond instead (Figure S41).

Figure S60. Natural charges and Wiberg bond indices (bold, arrow) for selected triazabutadienes as derived from NBO calculations on the PBE0-D3/def2TZVP//PBE0-D3/def2SVP level of theory.

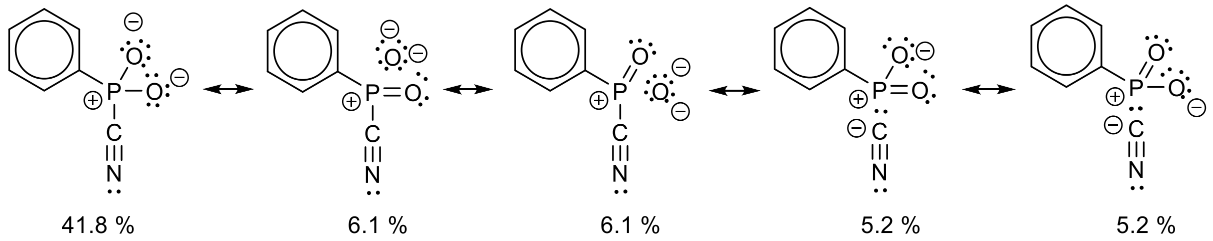

Figure S61. Natural charges and Wiberg bond indices (bold, arrow) for selected triazabutadienes as derived from NBO calculations on the PBE0-D3/def2TZVP//PBE0-D3/def2SVP level of theory.

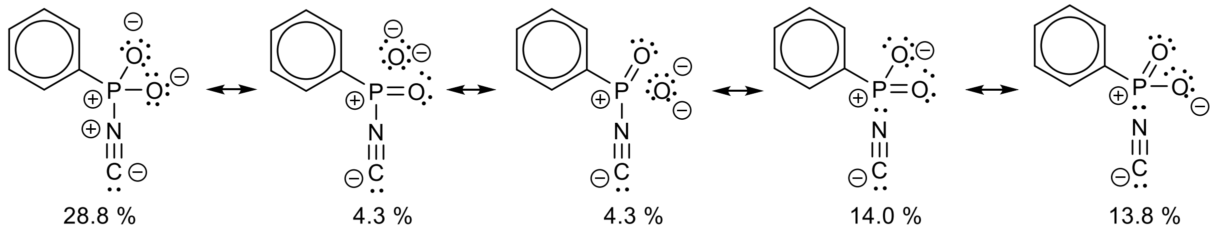

Figure S62. Natural charges and Wiberg bond indices (bold, arrow) for selected triazabutadienes as derived from NBO calculations on the PBE0-D3/def2TZVP//PBE0-D3/def2SVP level of theory.

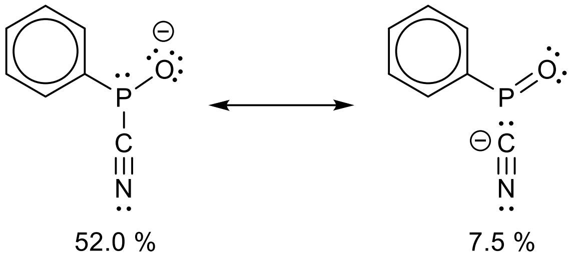

Table S9. Absolute energies (in a.u.) based on coupled cluster methods, E_{CC} (DLPNO-CCSD(T)/def2-TZVP) and and correction to Gibbs free energy (Cor.) at the PBE0-D3/def2-TZVP level of theory.

Compd.	$[PhPO_2(CN)]^-$	$[PhPO_2(NC)]^-$	$[PhPOCN]^-$
q(P)	2.12	2.27	1.10
q(O1)	−1.12	−1.12	−1.13
q(O2)	−1.12	−1.12	-
$q(C_{CN})$	−0.09	−0.84	−0.06
$q(N_{CN})$	−0.40	0.21	−0.45
WBI ($P-C_{CN}$)	0.72	-	0.82
WBI ($P-N_{CN}$)	-	0.58	-
WBI (C≡N)	2.91	2.44	2.86
WBI (P−O1)	1.15	1.17	1.16
WBI (P−O2)	1.15	1.17	-

Figure S63. Selected NLMOs of $[PhPO_2(CN)]^-$ (PBE0−D3/def2−TZVP, isovalue = 0.04).

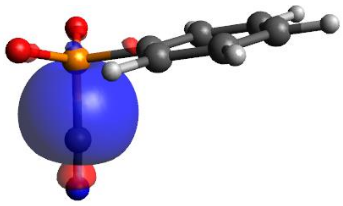

σ(P−C)
−13.6 eV
P: 31%
(s: 29%, p: 69%)
C: 68%
(s: 51%, p: 49%)

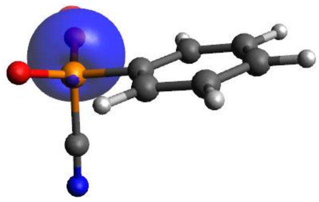

σ(P−O)
−20.8 eV
P: 26%
(s: 35%, p: 63%)
O: 73%
(s: 35%, p: 64%)

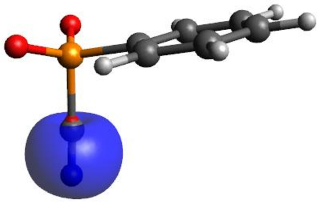

σ(C−N)
−26.0 eV
C: 42%
(s: 47%, p: 53%)
N: 58%
(s: 48%, p: 51%)

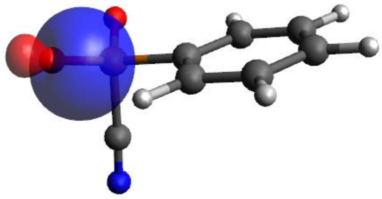

σ(P−O)
−20.8 eV
P: 26%
(s: 35%, p: 63%)
O: 73%
(s: 35%, p: 64%)

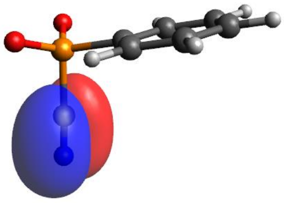

π(C−N)
−5.9 eV
C: 44%
(s: 0%, p: 100%)
N: 56%
(s: 0%, p: 100%)

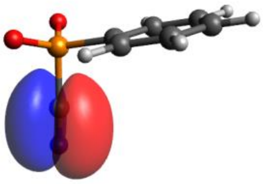

π(C−N)
−6.0 eV
C: 45%
(s: 0%, p: 100%)
N: 55%
(s: 0%, p: 100%)

Figure S64. Selected NLMOs of $[PhPO_2(NC)]^-$ (PBE0−D3/def2−TZVP, isovalue = 0.04).

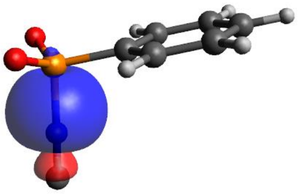

σ(P−N)
−17.6 eV
P: 22%
(s: 27%, p: 72%)
N: 78%
(s: 44%, p: 56%)

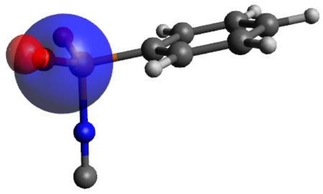

σ(P−O)
−20.8 eV
P: 26%
(s: 36%, p: 62%)
O: 73%
(s: 35%, p: 64%)

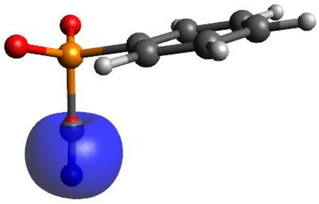

σ(N−C)
−26.0 eV
C: 34%
(s: 33%, p: 67%)
N: 66%
(s: 53%, p: 46%)

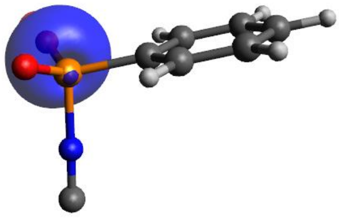

σ(P−O)
−20.8 eV
P: 26%
(s: 36%, p: 62%)
O: 73%
(s: 35%, p: 64%)

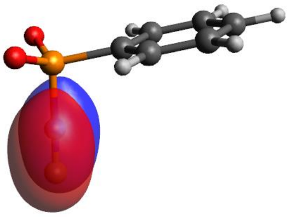

π(N−C)
−6.5 eV
C: 28%
(s: 0%, p: 100%)
N: 72%
(s: 0%, p: 100%)

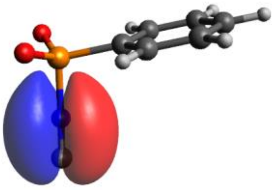

π(N−C)
−6.6 eV
C: 27%
(s: 0%, p: 100%)
N: 72%
(s: 0%, p: 100%)

Figure S65. Selected NLMOs of $[PhPO(CN)]^{-}$ (PBE0−D3/def2−TZVP, isovalue = 0.04).

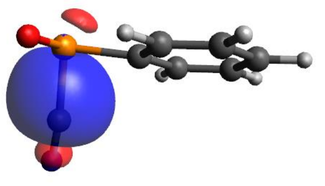

σ(P−C)
−12.0 eV
P: 30%
(s: 15%, p: 84%)
C: 68%
(s: 54%, p: 46%)

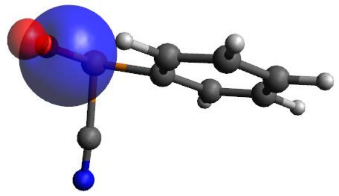

σ(P−O)
−19.5 eV
P: 26%
(s: 26%, p: 73%)
O: 73%
(s: 33%, p: 66%)

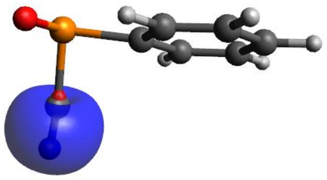

σ(C−N)
−25.4 eV
C: 41%
(s: 47%, p: 53%)
N: 59%
(s: 49%, p: 51%)

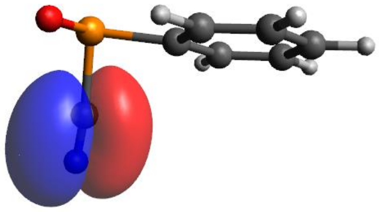

π(C−N)
−5.4 eV
C: 44%
(s: 0%, p: 100%)
N: 55%
(s: 0%, p: 100%)

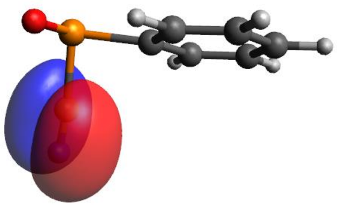

π(C−N)
−5.4 eV
C: 43%
(s: 0%, p: 100%)
N: 57%
(s: 0%, p: 100%)

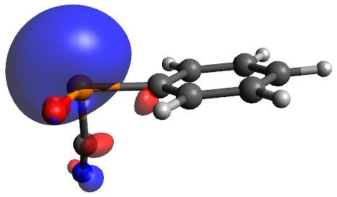

LP(N_{α})
−6.7 eV
P: 93%
(s: 54%, p: 46%)
C_{CN}: 2%
N_{CN}: 2%

5.6 Optimized structures (.xyz-files)

An xyz-file containing the coordinates of all optimized structures accompanies this submission and is available for download as additional material.

6 References

[1] P. Gupta, J.-E. Siewert, T. Wellnitz, M. Fischer, W. Baumann, T. Beweries, C. Hering-Junghans, *Dalton Trans.* **2021**, *50*, 1838-1844.

[2] G. M. Sheldrick, *Acta Cryst. A* **2015**, *71*, 3–8.

[3] G. M. Sheldrick, *Acta Cryst. C* **2015**, *71*, 3–8.

[4] X-AREA and LANA; Stoe & Cie: Darmstadt, Germany, **2020**.

[5] G. M. Sheldrick, *SADABS Version 2*, University of Göttingen, Germany, **2004**.

[6] O. V. Dolomanov, L. J. Bourhis, R. J. Gildea, J. A. K. Howard and H. Puschmann, *J. Appl. Cryst.* **2009**, 42, 339-341.

[7] M. Fischer, C. Hering-Junghans, *Chem. Sci.* **2021**, *12*, 10279-10289.

[8] Gaussian 09, Revision E.01, M. J. Frisch, G. W. Trucks, H. B. Schlegel, G. E. Scuseria, M. A. Robb, J. R. Cheeseman, G. Scalmani, V. Barone, G. A. Petersson, H. Nakatsuji, X. Li, M. Caricato, A. Marenich, J. Bloino, B. G. Janesko, R. Gomperts, B. Mennucci, H. P. Hratchian, J. V. Ortiz, A. F. Izmaylov, J. L. Sonnenberg, D. Williams-Young, F. Ding, F. Lipparini, F. Egidi, J. Goings, B. Peng, A. Petrone, T. Henderson, D. Ranasinghe, V. G. Zakrzewski, J. Gao, N. Rega, G. Zheng, W. Liang, M. Hada, M. Ehara, K. Toyota, R. Fukuda, J. Hasegawa, M. Ishida, T. Nakajima, Y. Honda, O. Kitao, H. Nakai, T. Vreven, K. Throssell, J. A. Montgomery, Jr., J. E. Peralta, F. Ogliaro, M. Bearpark, J. J. Heyd, E. Brothers, K. N. Kudin, V. N. Staroverov, T. Keith, R. Kobayashi, J. Normand, K. Raghavachari, A. Rendell, J. C. Burant, S. S. Iyengar, J. Tomasi, M. Cossi, J. M. Millam, M. Klene, C. Adamo, R. Cammi, J. W. Ochterski, R. L. Martin, K. Morokuma, O. Farkas, J. B. Foresman, and D. J. Fox, Gaussian, Inc., Wallingford CT, **2016**.

[9] Gaussian 16, Revision C.02, M. J. Frisch, G. W. Trucks, H. B. Schlegel, G. E. Scuseria, M. A. Robb, J. R. Cheeseman, G. Scalmani, V. Barone, G. A. Petersson, H. Nakatsuji, X. Li, M. Caricato, A. V. Marenich, J. Bloino, B. G. Janesko, R. Gomperts, B. Mennucci, H. P. Hratchian, J. V. Ortiz, A. F. Izmaylov, J. L. Sonnenberg, D. Williams-Young, F. Ding, F. Lipparini, F. Egidi, J. Goings, B. Peng, A. Petrone, T. Henderson, D. Ranasinghe, V. G. Zakrzewski, J. Gao, N. Rega, G. Zheng, W. Liang, M. Hada, M. Ehara, K. Toyota, R. Fukuda, J. Hasegawa, M. Ishida, T. Nakajima, Y. Honda, O. Kitao, H. Nakai, T. Vreven, K. Throssell, J. A. Montgomery, Jr., J. E. Peralta, F. Ogliaro, M. J. Bearpark, J. J. Heyd, E. N. Brothers, K. N. Kudin, V. N. Staroverov, T. A. Keith, R. Kobayashi, J. Normand, K. Raghavachari, A. P. Rendell, J. C. Burant, S. S. Iyengar, J. Tomasi, M. Cossi, J. M. Millam, M. Klene, C. Adamo, R. Cammi, J. W. Ochterski, R. L. Martin, K. Morokuma, O. Farkas, J. B. Foresman, and D. J. Fox, Gaussian, Inc., Wallingford CT, **2016**.

[10] F. Neese, *WIREs Comput. Mol. Sci.* **2018**, *8*, e1327.

[11] a) E. D. Glendening, J. K. Badenhoop, A. E. Reed, J. E. Carpenter, J. A. Bohmann, C. M. Morales, C. R. Landis, F. Weinhold, **2013**; b) J. E. Carpenter, F. Weinhold, *J. Mol. Struct.:*

THEOCHEM **1988**, *169*, 41–62; c) F. Weinhold, J. E. Carpenter, *The Structure of Small Molecules and Ions*, Plenum Press, **1988**; d) F. Weinhold, C. R. Landis, *Valency and Bonding. A Natural Bond Orbital Donor-Acceptor Perspective*, Cambridge University Press, **2005**.

[12] a) J. P. Perdew, K. Burke, M. Ernzerhof, *Phys. Rev. Lett.* **1996**, *77*, 3865–3868; b) J. P. Perdew, K. Burke, M. Ernzerhof, *Phys. Rev. Lett.* **1997**, *78*, 1396–1396; c) C. Adamo, V. Barone, *J. Chem. Phys.* **1999**, *110*, 6158–6170.

[13] a) S. Grimme, J. Antony, S. Ehrlich, H. Krieg, *J. Chem. Phys.* **2010**, *132*, 154104; b) S. Grimme, S. Ehrlich, L. Goerigk, *J. Comput. Chem.* **2011**, *32*, 1456–1465.

[14] F. Weigend, R. Ahlrichs, *Phys. Chem. Chem. Phys.* **2005**, *7*, 3297–305.

[15] I. M. Alecu, J. Zheng, Y. Zhao, D. G. Truhlar, *J. Chem. Theory Comput.* **2010**, *6*, 2872–2887.

[16] a) F. London, *J. Phys. Radium* **1937**, *8*, 397–409; b) R. McWeeny, *Phys. Rev.* **1962**, *126*, 1028–1034; c) R. Ditchfield, *Mol. Phys.* **1974**, *27*, 789–807; d) K. Wolinski, J. F. Hinton, P. Pulay, *J. Am. Chem. Soc.* **1990**, *112*, 8251–8260; e) J. R. Cheeseman, G. W. Trucks, T. A. Keith, M. J. Frisch, *J. Chem. Phys.* **1996**, *104*, 5497–5509.

[17] C. J. Jameson, A. De Dios, A. Keith Jameson, *Chem. Phys. Lett.* **1990**, *167*, 575–582.

[18] C. van Wüllen, *Phys. Chem. Chem. Phys.* **2000**, *2*, 2137–2144.

[19] a) C. Riplinger, B. Sandhoefer, A. Hansen and F. Neese, J. Chem. Phys., 2013, 139, 134101; b) C. Riplinger and F. Neese, J. Chem. Phys., 2013, 138, 034106; c) C. Adamo and V. Barone, J. Chem. Phys., 1999, 110, 6158-6170.

[20] V. Ásgeirsson, B. O. Birgisson, R. Bjornsson, U. Becker, F. Neese, C. Riplinger and H. Jónsson, *J. Chem. Theory Comput.*, 2021, **17**, 4929-4945.
